# Optimized treatment parameter by computer simulation for high-intensity focused ultrasound treatment of uterine adenomyosis: Short-term and long-term results

**DOI:** 10.1371/journal.pone.0301193

**Published:** 2024-03-28

**Authors:** Jae Seok Bae, Jae Young Lee, Hyun Hoon Chung, Maria Lee, Myung Jae Jeon, Hoon Kim, Hee Seung Kim, Kidong Kim, Chang-Soon Lee, Keonho Son, Joon Koo Han

**Affiliations:** 1 Department of Radiology, Seoul National University Hospital, Jongno-gu, Seoul, Republic of Korea; 2 Department of Radiology, Seoul National University College of Medicine, Jongno-gu, Seoul, Republic of Korea; 3 Institute of Radiation Medicine, Seoul National University Medical Research Center, Jongno-gu, Seoul, Republic of Korea; 4 Department of Obstetrics and Gynecology, Seoul National University Hospital, Jongno-gu, Seoul, Republic of Korea; 5 Department of Obstetrics and Gynecology, Seoul National University Bundang Hospital, Seongnam-si, Gyeonggi-do, Republic of Korea; 6 Department of Anesthesiology and Pain Medicine, Seoul National University Hospital, Jongno-gu, Seoul, Republic of Korea; 7 System Division, IMGT Co., Ltd., Healthcare Innovation Park, Bundang-gu, Seongnam-si, Korea; Kasr Alainy Medical School, Cairo University, EGYPT

## Abstract

This study aimed to investigate the efficacy and safety of using optimized parameters obtained by computer simulation for ultrasound-guided high-intensity focused ultrasound (HIFU) treatment of uterine adenomyosis in comparison with conventional parameters. We retrospectively assessed a single-institution, prospective study that was registered at Clinical Research Information Service (CRiS) of Republic of Korea (KCT0003586). Sixty-six female participants (median age: 44 years) with focal uterine adenomyosis were prospectively enrolled. All participants were treated with a HIFU system by using treatment parameters either for treating uterine fibroids (Group A, first 20 participants) or obtained via computer simulation (Group B, later 46 participants). To assess the treatment efficacy of HIFU, qualitative indices, including the clinically effective dysmenorrhea improvement index (DII), were evaluated up to 3 years after treatment, whereas quantitative indices, such as the nonperfused volume ratio and adenomyosis volume shrinkage ratio (AVSR), on MRI were evaluated up to 3 months after treatment. Quantitative/qualitative indices were compared between Groups A and B by using generalized linear mixed effect model. A safety assessment was also performed. Results showed that clinically effective DII was more frequently observed in Group B than in Group A (odds ratio, 3.69; P = 0.025), and AVSR were higher in Group B than in Group A (least-squares means, 21.61; P = 0.001). However, two participants in Group B developed skin burns at the buttock and sciatic nerve pain and required treatment. In conclusion, parameters obtained by computer simulation were more effective than the conventional parameters for treating uterine adenomyosis by using HIFU in terms of clinically effective DII and AVSR. However, care should be taken because of the risk of adverse events.

## Introduction

Adenomyosis is a common gynecologic disease that affects 20.9–36.2% of women during their reproductive years [1,2]. Approximately two-thirds of those patients diagnosed with adenomyosis have symptoms including dysmenorrhea, menorrhagia, and metrorrhagia [3]. The management options for symptomatic adenomyosis range from surgery to medical treatment. Although hysterectomy is still considered to be a definitive treatment [4], it is unsuitable for women who wish to retain their uterus. Conservative uterine-sparing surgery is also problematic because of the indistinct border between the ectopic foci of endometrial tissue and normal myometrium [5]. Medical treatments, including gonadotropin-releasing hormone agonists, progestogens, and nonsteroidal anti-inflammatory drugs, are frequently used for symptom relief [6]. However, side effects of the drugs may occur, and symptoms tend to recur shortly after the cessation of treatment [7,8]. Therefore, other uterine-sparing treatments, including uterine artery embolization, levonorgestrel-releasing intrauterine devices, and high-intensity focused ultrasound (HIFU), have been recently investigated [9–11].

HIFU is the most recently developed noninvasive technique that induces thermal ablation in the target lesion by focusing beams of ultrasound waves at the desired point with minimal or no damage to the surrounding normal tissue [12,13]. In addition to the brain, prostate gland, bone, or uterine leiomyomas [13–17], HIFU has been successfully applied for the treatment of uterine adenomyosis [18–21]. Moreover, there have been technological advances in the HIFU system that have enabled safer and more effective treatments, which have been demonstrated in preclinical and clinical studies targeting uterine fibroids [22,23]. However, the optimal parameters of HIFU treatment for uterine adenomyosis have not yet been established. Therefore, this study aimed to assess the efficacy and safety of ultrasound-guided HIFU treatment for uterine adenomyosis with an optimized treatment parameter obtained via computer simulation.

## Materials and methods

### 1. Participants

This study is a retrospective analysis of a prospective study. The original prospective study aimed to demonstrate a noninferiority of a portable ultrasound-guided HIFU treatment system compared to a conventional ultrasound-guided HIFU treatment system. During the original study, modification of HIFU treatment parameters via computer simulation was performed to enhance the treatment effect, and in this study, we retrospectively compared the outcome of two HIFU treatment parameters. The original prospective study was approved by the Institutional Review Board of our hospital (H-1506-150-685, Seoul, Korea) on 19 August 2015. Written informed consent was obtained from all of the participants. The original study was registered at Clinical Research Information Service (CRiS) of Republic of Korea (https://cris.nih.go.kr, KCT0003586). Registration of the original study to the CRiS was delayed because it was not common at the beginning of the study to register clinical trials in our department. As the study progressed, awareness of clinical trial registration was raised, and we registered, albeit belatedly. The authors confirm that all ongoing and related trials for this drug/intervention are registered. The data of the original prospective study was lastly accessed on 29 June 2023. The authors had access to information that could identify individual participants after data collection.

**Fig 1:**
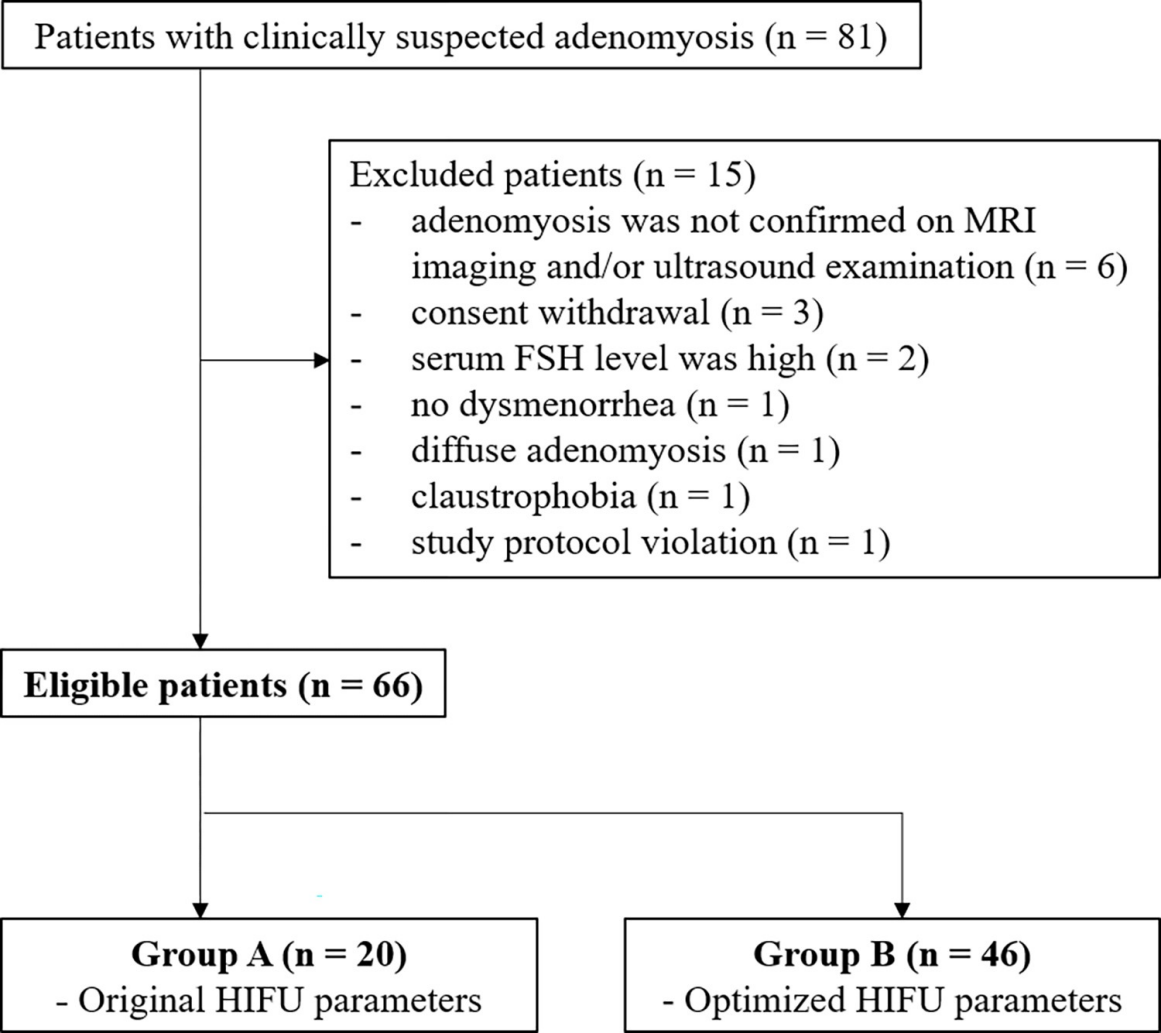
Flow diagram of the study population. MRI = magnetic resonance imaging, FSH = follicular stimulating hormone, HIFU = high-intensity focused ultrasound.

Participants were recruited through the outpatient clinics of the Department of Obstetrics and Gynecology in Seoul National University Hospital and Seoul National University Bundang Hospital between 14 January 2016 and 4 December 2017. Participants with uterine adenomyosis suspected on ultrasound and magnetic resonance imaging (MRI) were enrolled (**Fig 1**) [24,25]. Only focal uterine adenomyosis that was defined as a circumscribed mass within the myometrium was included for the analysis [26]. The inclusion and exclusion criteria, as well as the checklists for the study protocol, are shown in **Tables 1** and **S1**.

**Table 1:** Inclusion and exclusion criteria.

**Inclusion criteria**	≥ 20 years old Premenopausal or perimenopausal (FSH < 40 mIU/ml) Focal uterine adenomyosis was diagnosed on ultrasound and MRI Dysmenorrhea score ≥ 4 No previous treatment for uterine adenomyosis within 3 months
**Exclusion criteria**	Presence of other malignant pelvic tumors, endometriosis, acute pelvic disease, or other pelvic disease. Positive pregnancy test or anticipated pregnancy in the future Severe systemic disease Hematocrit < 25% Extensive scar tissue > 50% of anterior abdominal wall Presence of scar or surgical clips within beam pathway Contraindication for contrast-enhanced MRI Diffuse uterine adenomyosis Judged inappropriate to be enrolled in this study by investigators

FSH = follicle-stimulating hormone, MRI = magnetic resonance imaging.

### 2. Pretreatment and posttreatment imaging

Before treatment, ultrasound examination was performed on the day when the participants visited our hospital for screening MRI to determine if there was scar tissue or an intervening bowel loop in the HIFU beam path that may affect the visibility of the target lesions. MRI was performed by using a 3.0 T machine (Ingenia, Philips) before treatment, immediately after HIFU treatment, and 1 month and 3 months after HIFU treatment. The details of the MRI procedure are described in the **S1 Appendix**.

### 3. HIFU treatment

HIFU treatment was performed in Seoul National University Hospital. All of the participants underwent fasting from midnight until the procedure and received skin preparations. When bladder filling was determined to be needed on ultrasound, a Foley catheter was inserted into the bladder, and degassed normal saline was infused to control the bladder volume for treatment. In addition, the rectum was filled with approximately 100 mL of sonography gel (Supersonic, Sungheung).

All of the HIFU procedures were performed by using the HIFU device (ALPIUS 900, Alpinion Medical Systems) by one radiologist who had 7 years of experience with HIFU treatment. The protocol of HIFU treatment in our institution has been previously reported [23]. Briefly, after placing the treatment head on the abdomen of the participant while they were lying in the supine position, the temperature of the water surrounding the treatment head was reduced to 4°C to prevent burns. The treatment field was determined by using 3D planar images that is automatically created after automatic scanning of imaging transducer to enable visualization of the target tumor on 3D plane, and the location where the actual HIFU beam is being focused was explored by applying a targeted forecasting function, which forecasts the point where HIFU treatment would be performed by using low energy ultrasound waves. We aimed to treat the entirety of the focal adenomyosis volume. To achieve this, our HIFU treatment targeted a minimum of one centimeter above the deepest margin of the adenomyosis. Treatment was initiated after it was confirmed that the HIFU beam was focused on the target area. The detailed functions of HIFU treatment is described in **S2 Appendix**. Only uterine adenomyosis that exhibited contrast enhancement on MRI was treated.

Initially, HIFU treatment was performed using the same parameters for the treatment of uterine fibroids. As the study progressed, however, new parameters were obtained through computer simulation to enhance the treatment effect by considering the difference between uterine adenomyosis and uterine fibroids. For example, in general, on T2-weighted image, the uterine fibroids are as dark as skeletal muscle, while adenomyosis has a brightness similar to or higher than that of the myometrium. The brightness on T2-weighted image may be related to the perfusion [27]. Therefore, the perfusion coefficient for uterine fibroids was set as that of the muscle to solve Penn’s bioheat transfer equation, whereas the perfusion coefficient for adenomyosis was set as that of the uterine myometrium. In the simulation algorithm, the Rayleigh Sommerfeld diffraction Integral and angular spectrum approach were used to calculate the acoustic field generated by the phased array transducer, and the bio-heat transfer equation was used to calculate the temperature field based on the obtained acoustic field [28–30]. Accordingly, two groups were developed: a group consisting of the first 20 participants who were treated by using the same parameters as for the uterine fibroids (Group A) and the other group consisting of the later 46 participants who were treated by using the new parameters (Group B). The details of the computer simulation for optimizing the parameters are described in the **S3 Appendix**. The HIFU parameters were as follows: for Group A, an interval of 4 mm, an intensity of 0.8 kW/cm^2^, a duty cycle of 60%, a center frequency of 1.0 MHz, a pulse repetition frequency of 10 Hz, a per point treatment time of 6 sec, and an interpoint transition time of 3 sec; and for Group B, an interval of 2 mm, an intensity of 1.0 kW/cm^2^, a duty cycle of 70%, a center frequency of 1.0 MHz, a pulse repetition frequency of 10 Hz, a per point treatment time of 7 sec, and an interpoint transition time of 3 sec. In terms of the mode of anesthesia, all of the participants in Group A received monitored anesthesia care (MAC); in Group B, the first 16 participants received MAC, and the later 30 participants received epidural anesthesia (EA). A previous study from our institution summarized the details of MAC and EA and evaluated the association between the mode of anesthesia and the HIFU treatment outcome [31]. The participants were blinded to this allocation based on treatment parameters.

### 4. Efficacy assessment

The efficacy of HIFU treatment was assessed in a qualitative and quantitative manner. For qualitative assessment, a clinically effective relief of dysmenorrhea, as assessed by using the dysmenorrhea improvement index (DII), was used. The DII was used for the evaluation of the improvement of the quality of life related to dysmenorrhea on a five-point scale as follows: 1, complete relief; 2, partial (50–99%) relief; 3, minor (1–49%) relief; 4, ineffective; and 5, exacerbated pain. Clinically effective DII was defined as a score ≤ 3, and was considered the primary outcome. In addition, other secondary outcomes including the dysmenorrhea score, menorrhagia score, uterine fibroid symptom and quality of life questionnaire (UFS-QOL), 36-item short-form health survey version 2 (SF36-v2), and symptom severity score (SSS) were measured [32,33]. The dysmenorrhea score and menorrhagia score were assessed on a five-point scale as follows: 1, not at all; 2, a little bit; 3, somewhat; 4, a great deal; and 5, a very great deal. The UFS-QOL and SF36-v2 were used for the evaluation of the quality of life. These scores were recorded in the outpatient clinic prior to treatment and at 1 and 3 months after treatment. The results of up to 3 months of follow-up were considered as the short-term follow-up, and participants were paid for transportation for 1- and 3-month follow-up visits. For long-term follow-up, telephone interviews were conducted to evaluate the DII, dysmenorrhea score, and menorrhagia score at 1 year and 3 years after treatment. The last date of long-term follow-up was 4 December 2020.

**Fig 2:**
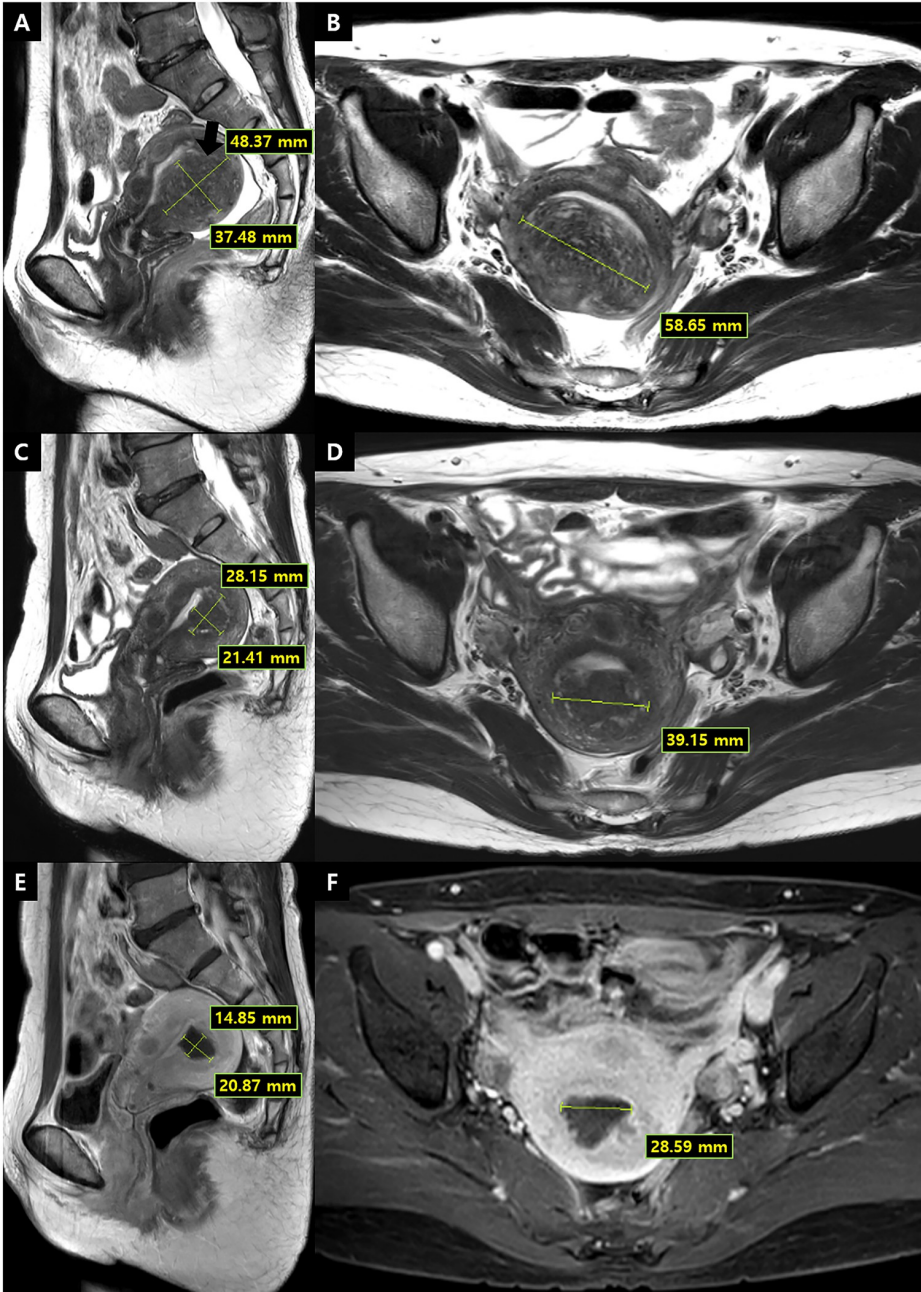
Examples of quantitative assessment of treatment efficacy on MR images of a 50-year-old woman. **(A, B)** Pre-HIFU sagittal and axial T2-weighted images show an adenomyotic lesion at the posterior wall of the uterus. The volume of the adenomyotic lesion was calculated as 0.523 × length × width × height (lines) and was equal to 60.8 cm^3^. **(C–F)** Three-month follow-up images of the same participant. **(C, D)** Sagittal and axial T2-weighted images show shrinkage of the adenomyotic lesion. The adenomyosis volume was calculated as 12.0 cm^3^, and the adenomyosis volume shrinkage rate was 80.3%. **(E, F)** Contrast-enhanced T1-weighted images demonstrate nonperfused volume, which was equal to 8.3 cm^3^. The calculated nonperfused volume ratio in this participant was 69.1%.

For the quantitative assessment, we calculated the nonperfused volume ratio (NPVR; nonperfused volume divided by the original adenomyosis volume that was targeted) on MRI performed immediately after HIFU treatment and adenomyosis volume shrinkage ratio (AVSR) on the follow-up MRI (**Fig 2**). The volume of adenomyosis and nonperfused volume were measured in three orthogonal directions on MRI by using the following equation: 0.523 × length × width × height [34]. AVS was calculated as 1 - (adenomyosis volume after HIFU treatment/original adenomyosis volume before HIFU treatment) at the 1- and 3-month follow-up MRI. The quantitative assessment was performed only for the short-term follow-ups.

### 5. Safety assessment

Safety was another primary outcome of our study. For the safety assessment, we inspected the participants for the presence of skin burns, abdominal wall injury, intra-abdominal organ injury, foot drop or sensory change for sciatic nerve injury, urination difficulty, persistent pain, or internal bleeding. All of the participants were instructed to promptly contact the HIFU team if they experienced adverse events. Adverse events were classified according to the Society of Interventional Radiology (SIR) guidelines [35].

### 6. Statistical analysis

Data are presented as the medians with quartiles 1 and 3 or percentages, as appropriate. The Mann–Whitney U test was used to compare the continuous variables, and the Fisher’s exact test was used to compare the categorical variables. A generalized linear mixed effect model was used to assess the difference between two groups and the modes of anesthesia (i.e. MAC vs. EA) for a longitudinally measured outcome. The cumulative logit link function was applied for the multinomial outcome such as dysmenorrhea score or menorrhagia score. The logit and identity link function was applied for binary and continuous outcome. The fixed effects were group, time at measurement, the interaction between group and time, and baseline value of the outcome. The random effect was a subject. Based on clinical interest in assessing whether the effect of the optimized treatment parameter differs between short-term and long-term outcomes, the interaction between group and time for outcomes with long-term follow-up was tested, yielding non-significant results (P > 0.05). The difference between groups at 3 months and 3 years after treatment was then estimated. Generalized linear mixed effect model were performed to identify variables associated with short-term and long-term HIFU treatment effect. Multivariable analyses were performed by using a backward selection, in which all variables with a *P* value < 0.10 in the univariable analyses. All of the statistical analyses were performed with commercially available statistical software (MedCalc version 18.9, MedCalc Software; PASS 13, NCSS statistics software; SAS version 9.4, SAS institute). A difference with a *P* value < 0.05 was considered to be statistically significant.

## Results

### 1. Participant characteristics

Among the 81 participants with clinically suspected uterine adenomyosis, 15 participants were excluded for the following reasons: adenomyosis was not confirmed on MRI and/or ultrasound examination (n = 6), consent withdrawal after HIFU treatment for personal reasons (n = 3), serum follicular stimulating hormone level was high (n = 2), no dysmenorrhea (n = 1), diffuse adenomyosis (n = 1), claustrophobia (n = 1), or a study protocol violation (intake of steroids because of underlying systemic lupus erythematosus) (n = 1) (**Fig 1**). Finally, 66 participants were included in the study (**Table 2**). Detailed clinical information of each patient including symptom before HIFU treatment, size and location of uterine adenomyosis is presented in **S1 File** and **S2 Table**.

**Table 2:** Characteristics of the participants.

	Total (n = 66)	Group A (n = 20)	Group B (n = 46)	*P* value
**Baseline variables**				
Age, y	44.0 (41.0–46.0)	44.5 (41.5–46.0)	43.5 (41.0–46.0)	0.711
Body mass index, kg/m^2^	22.9 (20.8–24.4)	22.1 (20.9–25.6)	23.0 (20.7–24.1)	0.681
Volume of uterus, cm^3^	258.1 (182.9–361.5)	250.0 (214.3–352.4)	258.1 (172.9–393.8)	0.840
Volume of adenomyosis, cm^3^	68.6 (37.2–149.1)	75.9 (42.5–125.3)	66.6 (35.6–150.7)	0.939
Coexisting uterine myoma (yes/no)	10/56	2/18	8/38	0.711
Abdominal surgical scar (yes/no)	16/50	4/16	12/34	0.758
**Inter-/Post-treatment variables**				
Treatment time, min	91.5 (75.0–118.0)	93.5 (74.5–118.5)	88.5 (75.0–118.0)	0.845
Sonication time, min	40.2 (26.9–49.9)	27.4 (21.8–44.5)	43.1 (30.8–53.9)	0.015*
Acoustic power, W	178.3 (153.8–212.9)	171.7 (146.2–199.4)	181.7 (154.9–215.3)	0.286
Pain related to HIFU treatment	4 (3–5)	5 (4–5)	3 (3–5)	0.020*

Values are presented as medians (interquartile ranges), otherwise indicated. HIFU = high-intensity focused ultrasound.

*P < 0.050.

### 2. Qualitative assessment

The clinically effective DII, the primary endpoint, was highly associated with Group B than in Group A (odds ratio, 3.69; P = 0.025) (**Table 3**). Meanwhile, the dysmenorrhea score and menorrhagia score did not demonstrate significant difference between Group A and Group B (odd ratio, 1.89 and 2.52, respectively) (P = 0.218 and 0.073, respectively). The results of other qualitative indices, such as UFS-QOL, SF-36v2, and SSS, are presented in **Table 3**. The factors independently impacting qualitative HIFU treatment outcomes were as follows: BMI and parameter group for DII, acoustic power and pain related to HIFU treatment for menorrhagia score, and volume of uterus for SSS (**S3 Table**).

**Table 3:** Qualitative measurement of treatment efficacy between groups.

		Group A (n = 20)	Group B (n = 46)	Odds ratio or Ls means^†^	95% CI	*P* value
Clinically effective DII^††^	1 m F/U	75.0 (15/20)	87.0 (40/46)	3.69	1.19, 11.45	0.025*
	3 m F/U	80.0 (16/20)	100.0 (46/46)			
	1 y F/U	63.2 (12/19)	89.7 (35/39)			
	3 y F/U	66.7 (10/15)	74.1 (20/27)			
Dysmenorrhea score	Screening	4.5 (4–5)	5 (4–5)	1.89	0.68, 5.26	0.218
	1 m F/U	3 (1.5–4)	3 (2–3)			
	3 m F/U	3 (2–4)	2 (1–2)			
	1 y F/U	2 (2–3.8)	2 (2–3)			
	3 y F/U	3 (1–3)	2 (2–3)			
Menorrhagia score	Screening	4 (4–5)	4.5 (4–5)	2.52	0.92, 6.96	0.073
	1 m F/U	3 (1.5–3)	2 (1–3)			
	3 m F/U	2.5 (2–4)	2 (1–3)			
	1 y F/U	2 (2–3.8)	1 (1–2)			
	3 y F/U	2 (1–3)	2 (1–3)			
UFS-QOL	Screening	117.5 (92.5–131.5)	125.5 (101–138)	-1.65	-12.61, 9.32	0.765
	1 m F/U	84 (70–95.5)	85 (69–115)			
	3 m F/U	77.5 (58.5–93.5)	70.5 (53–88)			
SF-36v2	Screening	114 (103.5–119)	111 (105–117)	-1.90	-5.74, 1.95	0.328
	1 m F/U	116 (113–118.5)	113 (106–120)			
	3 m F/U	121 (113–124)	116 (112–122)			
SSS	Screening	57.5 (42.5–73.5)	57.5 (44–72)	-1.54	-8.50, 5.42	0.660
	1 m F/U	31 (23.5–47)	32.5 (25–50)			
	3 m F/U	34 (22–47)	25 (19–34)			

Values are presented as medians (quartiles 1–3), otherwise indicated. CI = confidence interval.

^†^Odds ratios were obtained by using generalized linear mixed models for clinically effective DII, dysmenorrhea score, and menorrhagia score. Ls means were obtained by using mixed models for UFS-QOL, SF-36v2, and SSS.

^††^Number are percentages with proportions in parentheses.

*P < 0.050.

In terms of the modes of anesthesia, there was no significant difference between MAC and EA (**S4 Table**).

### 3. Quantitative assessment

**Fig 3:**
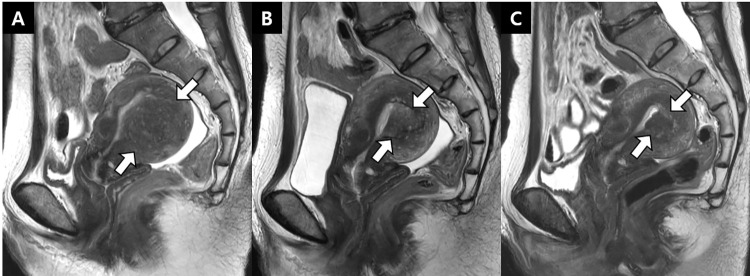
A representative case of adenomyosis in a 50-year-old woman in group B. **(A)** Sagittal T2-weighted image obtained at screening show a uterine adenomyosis (arrow) (5.7 cm × 5.1 cm × 4.0 cm) at the uterine posterior body. **(B, C)** One-month and three-month follow-up sagittal T2-weighted images, respectively. The volume of the adenomyosis (arrowheads) further shrunken, and the adenomyosis volume shrinkage ratios were 67.0% and 80.3%, respectively.

NPVR and AVSR were significantly higher in Group B than in Group A (Ls means, 26.92 and 21.61, respectively) (*P* = 0.001 for both) (**Table 4** and **Fig 3**). On the contrary, volume of adenomyosis and nonperfused volume were not significantly different between Group A and B (Ls means, -7.95 and 7.71, respectively) (P = 0.315 and 0.166, respectively) (**Table 4** and **Fig 3**). The factors independently impacting quantitative HIFU treatment outcomes were as follows: acoustic power for the volume of adenomyosis, the volume of uterus and the volume of adenomyosis for NPV, the volume of uterus and epidural anesthesia for NPVR, and volume of adenomyosis and parameter group for AVSR (**S5 Table**).

**Table 4:** Quantitative measurement of treatment efficacy between groups.

		Group A (n = 20)	Group B (n = 46)	Ls means	95% CI	*P* value
Volume of adenomyosis, cm^3^	Immediate	75.9 (42.5–125.3)	66.6 (35.6–150.7)	-3.38	-14.12, 7.35	0.532
	1 m F/U	71.2 (32.4–139.4)	60.7 (28.5–120.8)			
	3 m F/U	66.1 (33.4–119.0)	31.3 (12.7–97.9)			
Nonperfused volume, cm^3^	Immediate	20.7 (10.0–80.1)	51.0 (17.6–110.2)	22.20	-19.87, 64.27	0.296
	1 m F/U	6.9 (0.3–73.3)	28.1 (7.5–99.9)			
	3 m F/U	0.8 (0.0–31.1)	11.1 (2.1–32.6)			
NPVR, %	Immediate	34.1 (12.1–79.4)	77.1 (46.8–87.5)	26.92	11.15, 42.69	0.001*
	1 m F/U	10.0 (0.5–78.4)	69.2 (38.2–86.8)			
	3 m F/U	1.3 (0.0–46.1)	50.2 (15.7–83.1)			
AVSR, %	1 m F/U	21.5 (-2.7–31.7)	21.0 (7.4–42.8)	21.61	9.23, 33.99	0.001*
	3 m F/U	27.6 (3.4–40.8)	51.0 (35.0–68.8)			

Values are presented as medians (quartile 1–3). CI = confidence interval, NPVR = nonperfused volume ratio, AVSR = adenomyosis volume shrinkage ratio.

*P < 0.050.

In terms of the modes of anesthesia, NPVR was significantly higher in patients with EA than in patients with MAC (Ls mean, 34.17; *P* < 0.001) (**S6 Table**).

### 4. Safety assessment

There were 74 adverse events in 74.2% (49/66) of the participants, including mild (n = 71), moderate (n = 1), and severe (n = 2) events (**Table 5**). Among the mild adverse events, abdominal wall heat injury (defined as the contrast enhancement of the rectus muscle or subcutaneous fat on MRI) was noted in 34 participants. These participants complained of abdominal wall discomfort immediately after HIFU treatment, which was improved within one week without any treatment (n = 25) or with oral/intravenous analgesics (n = 9). In all of the 34 participants, the abdominal wall heat injury was completely resolved on MRI at the 3-month follow-up visit.

**Table 5:** SIR classification and distribution of adverse events.

SIR classification	Adverse events	Total (n = 66)	Group A (n = 20)	Group B (n = 46)
Mild	Lower abdominal pain	34	13	21
	Vaginal discharge	13	6	7
	Hip pain	10	1	9
	Nausea or vomiting	8	4	4
	Lower limb paresthesia	5	1	4
	Dysuria	1		1
Moderate	Burn injury	1		1
Severe	Lumbosacral plexus injury	1		1
	Rectal wall injury	1		1
Total		74	25	49

SIR = Society of Interventional Radiology.

**Fig 4:**
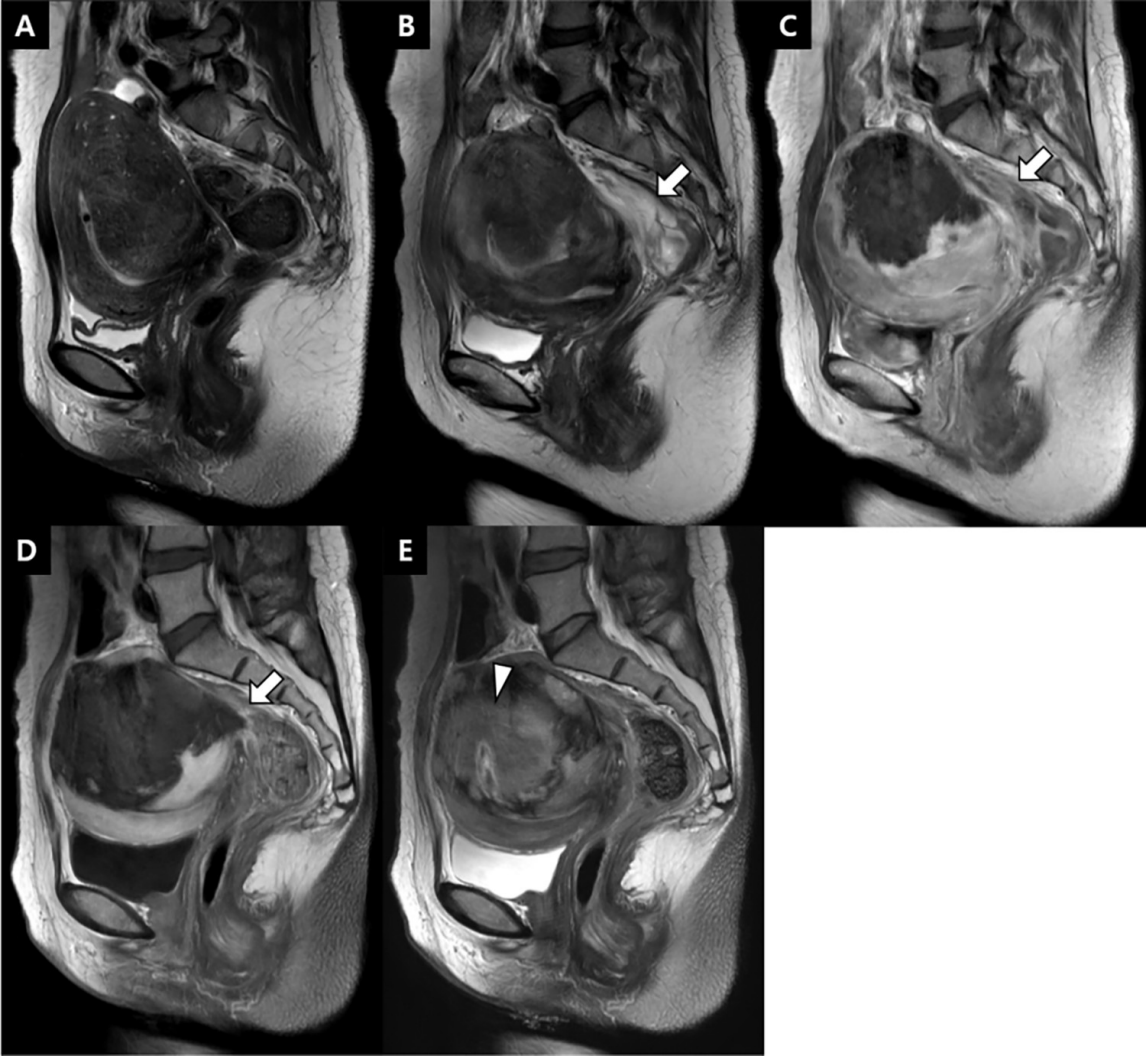
An adverse event after high-intensity focused ultrasound treatment. Sagittal T2-weighted images (A, B, E) and contrast-enhanced T1-weighted images (C, D) of a 46-year-old woman with adenomyosis (arrowhead). **(A)** Before HIFU treatment. **(B, C)** Increased signal intensity and enhancement along the anterior wall of the upper rectum adjacent to the adenomyosis was detected in the immediate posttreatment images (arrows). Her symptoms improved after conservative management, and she was discharged. **(D, E)** At the 1-month follow-up, the signal intensity of the rectal wall was normalized (arrow). Note the shrinkage of the treated adenomyosis (arrowhead).

All three participants with moderate or severe adverse events were in Group B. For moderate adverse events, a participant suffered a second-degree burn injury with bulla measured as 2 cm around the coccyx immediately after HIFU treatment, and a burn dressing was applied. She underwent EA and IV analgesics (remifentanyl and fentanyl) during HIFU treatment. Upon being checked after treatment, it was judged that she was too deeply sedated, given that the participant stated that she was able to tolerate pain, although she felt it. The participant was discharged on that day but was referred to the department of plastic surgery for the management of the burn injury. Debridement followed by dressing and antibiotic ointment application was performed. Regarding severe adverse events, a participant who underwent EA and IV analgesics (fentanyl) complained of lateral calf pain, and a unilateral sciatic nerve injury was confirmed in the nerve conduction study. Gabapentin was administered, and the pain was controlled after three months. The other participant who underwent MAC during treatment had lower abdominal pain after treatment, and rectal wall swelling was discovered in the immediate post-HIFU MRI (**Fig 4**). The participant was hospitalized and observed for five days without any eventful history and received conservative management. Rectal wall swelling resolved on the 1-month follow-up MRI.

The distribution of adverse events according to the modes of anesthesia is presented in **S7 Table**. Among the mild adverse events, lower abdominal pain and vaginal discharge were more frequently observed in MAC group (Ps ≤ 0.004), whereas hip pain was more common in EA group (P = 0.035). However, the occurrence of moderate or severe adverse events were not significantly different between MAC and EA groups (Ps ≥ 0.455).

## Discussion

In our study, HIFU treatment using the optimized parameters that were obtained via computer simulation (Group B) demonstrated a significantly better result than HIFU treatment using the conventional parameters that are used for the treatment of uterine fibroids (Group A) in terms of clinically effective DII (odds ratio, 3.69; P = 0.025), NPVR and AVSR (Ls mean, 26.92 and 21.61, respectively; P = 0.001 for both). The better result of the optimized parameters could be attributed to the rapid increase and sustainment of temperature by slightly increasing acoustic intensity, duty cycle, and per point treatment time. The optimized parameters, along with the acoustic power associated with the treatment parameter group, were independently linked to variable qualitative or quantitative outcomes. While a previous study reported that the mode of anesthesia (MAC vs. EA) was associated with NPVR in the treatment of uterine adenomyosis, their assessment was limited to immediate post-HIFU MRI [31]. In contrast, our study extended the follow-up period to 3 months or 3 years and employed appropriate statistical methods (specifically, generalized linear mixed model) to analyze the impact of various variables. Our results indicate that treatment parameters for HIFU, rather than the mode of anesthesia, are associated with treatment efficacy in uterine adenomyosis. Our findings suggest that optimizing parameters specifically for uterine adenomyosis may result in superior treatment outcomes compared to the conventional parameters commonly used for uterine fibroid treatment.

From the viewpoint of safety, the use of parameters that are advantageous for rapidly increasing tissue temperature to which thermal ablation occurs can lead to an increase in heat-related adverse events. This risk is even greater by depriving patients of the opportunity to complain of discomfort or heat sensations when it is combined with improper EA or an excessive in-depth sedation. The two cases of moderate and severe adverse events that required management, such as wound debridement or long-term medication, exclusively occurred in the participants who received the combination of EA and IV analgesics. EA requires professional knowledge and experiences to only anesthetize the desired dermatome. Therefore, to prevent clinically significant adverse events, it is essential that EA or sedation is not significant enough to result in complaints of heat-related discomfort. To do this, securing an anesthesiologist with extensive experience in EA and sedation is of paramount importance. Furthermore, it should be noted that the majority (96% [71/74]) of adverse events in this study were mild and required no or minimal therapy, which was similar to a recent study that reported 89.8–92.3% of intra- or postprocedural adverse events [36]. In terms of adverse events, we acknowledge the potential influence of a learning curve, particularly concerning deep sedation. Both the expertise of the radiologist performing the HIFU treatment and the anesthesiologist are pivotal factors in mitigating adverse events. While it’s challenging to specify an exact number of cases needed to overcome this learning curve, we estimate that approximately 60 cases within our study would be necessary to minimize the occurrence of severe adverse events.

There were a few limitations in our study. First, there was a sample size imbalance between the group A and group B, and participants were not randomly but sequentially assigned to each group, which could result in chronological bias. Secondly, this constitutes a secondary analysis of the original study; therefore, the sample size is insufficient to attain adequate statistical power for comparing between groups or assessing the interaction between groups and time. Third, there was a significant follow-up loss during the 3-year follow-up (from 66 participants to 42 participants). Fourth, at the 1- and 3-year follow-ups, only some qualitative analyses via telephone interviews were performed, not quantitative evaluations using MRI. However, the clinical significance of uterine adenomyosis is more often determined by the participants’ symptoms and not by quantitative measurements. Due to the fact that telephone interviews included major symptoms such as dysmenorrhea and menorrhagia, the results of this study may not have been significantly undermined. Furthermore, in the context of real-world clinical practice, a notable challenge arises from the variable wherein patients may opt to undergo additional treatments for uterine adenomyosis during the three-year follow-up period after a three-month hospital visit. This aspect complicates the determination of the treatment effect.

## Conclusion

### 1. Treatment efficacy

The parameters optimized by computer simulation were more effective than the conventional parameters for treating uterine adenomyosis.

### 2. Safety

Special care should be taken because the risk of heat-related adverse reactions also increases.

## Supporting information

S1 Checklist STROBE statement—checklist of items that should be included in reports of observational studies. (DOCX)

S1 Appendix MRI protocol. (DOCX)

S2 Appendix Ultrasound techniques in the HIFU machine. (DOCX)

S3 Appendix Computer simulation for optimizing HIFU parameters. (DOCX)

S1 Table Screening and monitoring check-up lists. (DOCX)

S2 Table Detailed clinical information of each patient. (DOCX)

S3 Table Factors impacting on the qualitative HIFU treatment outcomes. (DOCX)

S4 Table Qualitative measurement of treatment efficacy between the modes of anesthesia. (DOCX)

S5 Table Factors impacting on the quantitative HIFU treatment outcomes. (DOCX)

S6 Table Quantitative measurement of treatment efficacy between the modes of anesthesia. (DOCX)

S7 Table SIR classification and distribution of adverse events according to the modes of anesthesia. (DOCX)

S1 File Data of the participants. (XLSX)

S2 File (DOC)

S3 File (PDF)
