# Supplementary material for: Optimized treatment parameter by computer simulation for high-intensity focused ultrasound treatment of uterine adenomyosis: Short-term and long-term results
Source: PLoS One. 2024 Mar 28;19(3):e0301193. doi: 10.1371/journal.pone.0301193 (PMC10977802; doi:10.1371/journal.pone.0301193)
Supplement: S1 Appendix — (DOCX) [file pone.0301193.s002.docx]

**S1 Appendix. MRI protocol**

MRI protocol included axial T1-weighted and axial and sagittal T2-weighted images before contrast injection and axial and sagittal T1-weighted images after contrast agent injection. Gadoterate meglumine (Dotarem, Guerbet) was used at a dose of 0.1 mmol/kg as a contrast agent. The MRI parameters were as follows: T2 turbo-spin echo (TSE) sagittal (a field of view (FOV) of 250 × 250 mm^2^, an echo train (ET) of 20, a flip angle (FA) of 90.0°, a repetition time (TR)/echo time (TE) of 2835.1 ms/90.0 ms, 5.0 mm thickness/1.0 mm intersection gap, 1 number of excitation (NEX), and a matrix size of 512 × 367 mm^2^), T2 TSE axial (an FOV of 250×250 mm2, an ET of 20, an FA of 90.0°, a TR/TE of 3218.4–3475.7 ms/90.0 ms, 5.0 mm thickness/1.0 mm intersection gap, a NEX of 1, a matrix size of 512 × 355 mm^2^), precontrast fat-saturated T1 axial (an FOV of 250 × 250 mm^2^, an ET of 45, an FA of 10.0°, a TR/TE of 3.4 ms/1.7 ms, 3.0 mm thickness without intersection gap, a NEX of 1, and a matrix size of 252 × 190 mm^2^), postcontrast T1 axial (a FOV of 250 × 250 mm^2^, an ET of 45, an FA of 10.0°, a TR/TE of 3.4/1.7, 3.0 mm thickness without intersection gap, a NEX of 1, and a matrix size 252 × 190 mm^2^), and postcontrast T1 sagittal (an FOV 250 × 250 mm^2^, an ET of 6, an FA of 90.0°, a TR/TE of 511.0–549.7 ms/9.0 ms, 5.0 mm thickness/1.0 mm intersection gap, a NEX of 1, and a matrix size of 512 × 324 mm^2^) images.
