# Supplementary material for: Optimized treatment parameter by computer simulation for high-intensity focused ultrasound treatment of uterine adenomyosis: Short-term and long-term results
Source: PLoS One. 2024 Mar 28;19(3):e0301193. doi: 10.1371/journal.pone.0301193 (PMC10977802; doi:10.1371/journal.pone.0301193)
Supplement: S2 Appendix — (DOCX) [file pone.0301193.s003.docx]

**S2 Appendix. Ultrasound techniques in the HIFU machine**

*Targeted forecasting function*–The targeted forecasting function enables forecasting the point where the targeted HIFU treatment would be performed by using low energy ultrasound waves. It is achieved by utilizing a kind of passive cavitation mapping technique. A short pulse of ultrasound energy is emitted from all HIFU transducer elements simultaneously, which are used to create a sufficient peak negative pressure in the focus to induce non-inertial cavitation. The resulting bubble cloud in the focal area represents a spatially confined cloud of point scatterer. As a result, consecutive ultrasonic waves are then reflected by the cavitation bubbles back onto the image probe located at the center of the HIFU transducer. Finally, the cavitation map is acquired and displayed in order to visualize the HIFU focus through processing the reflected signals.

*3D Beam steering*–This machine has a 256-element transducer array that there is no need to move the whole transducer assembly in order to place lesions side by side when the clinically relevant volume should be treated. Electronic phasing of the signal to individual elements allows some dynamic control of a focal position, both axially and trans-axially without moving the transducer assembly. Based on the geometric focus, the focal position range resembles an egg shape, with an axial (Z axis) range of ± 25 mm and an X/Y range of ± 13 mm.

*Cavitation monitoring*–This feature enables operators to monitor the cavitation activity in real-time during HIFU exposure. When the acoustic pressure of ultrasound propagating in a medium is higher than a threshold value, one or more gas pockets, or bubbles can be generated. During HIFU treatment, these gas bubbles may grow and shrink in a stable fashion with the changing ultrasound pressure, but ultimately they might collapse, causing local energy release and temperature increase at the microscopic level. Although cavitation can be beneficial, the violent collapsing of bubbles may cause undesired tissue damage. Therefore, it is very important to monitor cavitation activity during HIFU treatments.

*Interleaved image*–For providing users real-time ultrasound monitoring images during HIFU exposure, it is important to remove HIFU-induced noise in the ultrasound image. Through the use of a sequence control between imaging and HIFU sonication control, noise-free ultrasound images can be acquired. If 70 % duty cycle of HIFU exposure is set, a pair of 0.07 sec HIFU exposure and 0.03 sec image acquisition continues to repeat until HIFU exposure terminates. Since image acquisition pauses in a very short time during HIFU exposure, almost real-time ultrasound images without HIFU induced noise can be generated.
