# Supplementary material for: Optimized treatment parameter by computer simulation for high-intensity focused ultrasound treatment of uterine adenomyosis: Short-term and long-term results
Source: PLoS One. 2024 Mar 28;19(3):e0301193. doi: 10.1371/journal.pone.0301193 (PMC10977802; doi:10.1371/journal.pone.0301193)
Supplement: S3 Appendix — (DOCX) [file pone.0301193.s004.docx]

**S3 Appendix. Computer simulation for optimizing HIFU parameters**

A computer simulation was performed to estimate the treatment efficacy according to the focal spot distribution. In the simulation algorithm, the Rayleigh Sommerfeld diffraction Integral [1] and Angular spectrum approach [2] were used to calculate the acoustic field generated by the phased array transducer, and the Bio-heat transfer equation [3] was used to calculate the temperature field based on the obtained acoustic field. Parameters were optimized from the simulation by predicting treatment efficiency from the relationship between input energy and thermal lesion volume generated by changes in sonication time per intensity/duty/point. To be specific, the following parameters were used: in situ intensity (Isppa): 1.0 kW/cm2, duty 60%, *attenuation: 8.1157 neper/mMHz, *heat capacity: 3676 J/kg/℃, *perfusion rate: 458 ml/min/kg, and *blood heat capacity: 3617 J/kg/℃. The parameters with asterisks were are the tissue parameters related to the uterus provided by IT’IS [4].

The HIFU transducer is a concave phased array consisting of 256 circular elements and has a focal length 11 cm and an aperture radius of 13 cm. The wave propagation path consists of a layer of 4 cm water, 3 cm abdomen wall layer, 1 cm uterine tissue and 6cm adenomyosis.

The three dimensional pressure field generated by the HIFU transducer was obtained as follows: the two-dimensional complex pressure field at the perpendicular plane to the z axis generated by each circular element was calculated with Rayleigh Sommerfeld diffraction integral. The pressure field for the phased array transducer was computed through the superposition of complex pressure produced by the array transducers after multiplying the complex pressure with the phase calculated according to electronic beam steering. The three-dimensional acoustic field was constructed by propagating the pressure field from depth z = 0 to depth z = 14 cm with 0.05 cm interval using the angular spectrum approach. The three dimensional acoustic field was calculated for every focal spot.

In order to calculate the temperature distribution in tissue, Bio-heat transfer equation was solved using finite difference time difference scheme based on the acoustic field. Thermal dose, an equivalent exposure time at 43 °C, was used to characterize the ablation outcome.

**REFERENCES**

1. Williams EG. Fourier Acoustics: Sound Radiation and Nearfield Acoustical Holography: Academic Press; 1999.

2. Zeng X, McGough RJ. Optimal simulations of ultrasonic fields produced by large thermal therapy arrays using the angular spectrum approach. The Journal of the Acoustical Society of America. 2009;125(5):2967-77.

3. Pennes HH. Analysis of tissue and arterial blood temperatures in the resting human forearm. Journal of applied physiology. 1948;1(2):93-122.

4. Society TFfRoITi. TISSUE PROPERTIES [cited 2024 February 21]. Available from: <https://itis.swiss/virtual-population/tissue-properties/database/>.
