# Supplementary material for: Optimized treatment parameter by computer simulation for high-intensity focused ultrasound treatment of uterine adenomyosis: Short-term and long-term results
Source: PLoS One. 2024 Mar 28;19(3):e0301193. doi: 10.1371/journal.pone.0301193 (PMC10977802; doi:10.1371/journal.pone.0301193)
Supplement: S1 Table — (DOCX) [file pone.0301193.s005.docx]

**S1 Table. Screening and monitoring check-up lists**

|  | Visit 1 | Visit 2 | Visit 3 | Visit 4 | Survey 5 |
| --- | --- | --- | --- | --- | --- |
|  | Day -30~0 | Day 0 | +1 month | + 3 months | + 1 year |
|  | (screening) | (HIFU treatment) | (follow-up) | (follow-up) | (follow-up) |
| Vital signs | √ | √ |  |  |  |
| Physical examination | √ |  |  |  |  |
| Past medical history | √ |  |  |  |  |
| Blood test^*^ | √ |  | √ |  |  |
| FSH | √ |  | √ |  |  |
| Pregnancy test | √ |  |  |  |  |
| MRI | √ | √ | √ | √ |  |
| Ultrasound | √ | √ | √ | √ |  |
| Dysmenorrhea improvement index | √ |  | √ | √ | √ |
| Dysmenorrhea score | √ |  | √ | √ | √ |
| Menorrhagia Score | √ |  | √ | √ | √ |
| SF-36v2 | √ |  | √ | √ |  |
| UFS-QOL (including SSS) | √ |  | √ | √ |  |
| Patients’ level of satisfaction with HIFU treatment |  | √ |  |  |  |
| Adverse events |  | √ | √ | √ | √ |

HIFU = high-intensity focused ultrasound, FSH = follicle-stimulating hormone, MRI = magnetic resonance imaging, SF-36v2 = 36-item short-form health survey version 2, UFS-QOL = uterine fibroid symptom and quality of life questionnaire, SSS = Symptom severity score.

*Complete blood count (Hemoglobin, Hematocrit, red blood cell count, white blood cell count with differential, platelet count) and electrolyte test (alkaline phosphatase, blood urea nitrogen, creatinine, alkaline aminotransferase, aspartate aminotransferase, albumin, total protein, total bilirubin, uric acid, glucose, cholesterol, lactate dehydrogenase, Na, K, Cl, and Ca). A pregnancy test was performed with a urine human chorionic gonadotropin test within 3 days before treatment, except in patients who had undergone an infertility operation or menopause.
