# Supplementary material for: Optimized treatment parameter by computer simulation for high-intensity focused ultrasound treatment of uterine adenomyosis: Short-term and long-term results
Source: PLoS One. 2024 Mar 28;19(3):e0301193. doi: 10.1371/journal.pone.0301193 (PMC10977802; doi:10.1371/journal.pone.0301193)
Supplement: S2 Table — (DOCX) [file pone.0301193.s006.docx]

**S2 Table. Detailed clinical information of each patient**

| **Patient number** | **HIFU parameter group** | **Main symptom before HIFU treatment** | **Location of uterine adenomyosis*** | **Volume of uterine adenomyosis, cm^3^*** |
| --- | --- | --- | --- | --- |
| 1 | A | Dysmenorrhea | Uterine body | 33.3 |
| 2 | A | Menorrhagia | Uterine body and fundus | 208.4 |
| 3 | A | Dysmenorrhea | Posterior wall of uterine body | 52.1 |
| 4 | A | Dysmenorrhea | Anterior and posterior wall of uterine body | 2.7 |
| 5 | A | Dysmenorrhea | Fundus | 110.4 |
| 6 | A | Irregular menstruation | Anterior and posterior wall of uterine body | 84.4 |
| 7 | A | Menorrhagia | Anterior and posterior wall of uterine body | 504.9 |
| 8 | A | Dysmenorrhea | Right lateral wall of uterine body | 70.4 |
| 9 | A | Dysmenorrhea | Anterior wall of uterine body and fundus | 140.2 |
| 10 | A | Menorrhagia | Uterine body and fundus | 214.2 |
| 11 | A | Dysmenorrhea | Anterior wall of uterine body | 60.1 |
| 12 | A | Dysmenorrhea | Uterine body and fundus | 100.8 |
| 13 | A | Dysmenorrhea | Fundus | 81.5 |
| 14 | A | Dysmenorrhea | Posterior wall of uterine body and fundus | 298.1 |
| 15 | A | Menorrhagia | Posterior wall of uterine body | 42.3 |
| 16 | A | Dysmenorrhea | posterior wall of uterine body and fundus | 60.6 |
| 17 | A | Menorrhagia | Anterior wall of uterine body | 103.5 |
| 18 | A | Dysmenorrhea | Posterior wall of uterine body | 7.0 |
| 19 | A | Dysmenorrhea | Posterior wall of uterine body | 42.7 |
| 20 | A | Menorrhagia | Posterior wall of uterine body | 23.7 |
| 21 | B | Dysmenorrhea | Fundus | 251.2 |
| 22 | B | Dysmenorrhea | Anterior wall of uterine body | 60.1 |
| 23 | B | Menorrhagia | Posterior wall of uterine body | 6.8 |
| 24 | B | Menorrhagia | Posterior wall of uterine body | 60.8 |
| 25 | B | Dysmenorrhea | Anterior and posterior wall of uterine body | 550.7 |
| 26 | B | Dysmenorrhea | Anterior wall of uterine body | 70.8 |
| 27 | B | Dysmenorrhea | Posterior wall of uterine body | 66.4 |
| 28 | B | Dysmenorrhea | Posterior wall of uterine body | 59.9 |
| 29 | B | Dysmenorrhea | Posterior wall of uterine body | 23.3 |
| 30 | B | Dysmenorrhea | Posterior wall of uterine body | 106.3 |
| 31 | B | Menorrhagia | Posterior wall of uterine body | 475.2 |
| 32 | B | Dysmenorrhea | Anterior wall of uterine body | 16.1 |
| 33 | B | Menorrhagia | Posterior wall of uterine body | 1.4 |
| 34 | B | Menorrhagia | Anterior wall of uterine body | 304.0 |
| 35 | B | Dysmenorrhea | Posterior wall of uterine body | 65.8 |
| 36 | B | Dysmenorrhea | Posterior wall of uterine body | 0.6 |
| 37 | B | Dysmenorrhea | Uterine body and fundus | 150.7 |
| 38 | B | Dysmenorrhea | Uterine body | 152.6 |
| 39 | B | Menorrhagia | Posterior wall of uterine body | 37.2 |
| 40 | B | Menorrhagia | Posterior wall of uterine body | 11.3 |
| 41 | B | Dysmenorrhea | Posterior wall of uterine body | 232.1 |
| 42 | B | Menorrhagia | Posterior wall of uterine body and fundus | 16.0 |
| 43 | B | Menorrhagia | Posterior wall of uterine body and fundus | 110.2 |
| 44 | B | Dysmenorrhea | Posterior wall of uterine body | 38.6 |
| 45 | B | Dysmenorrhea | Uterine body and fundus | 92.9 |
| 46 | B | Dysmenorrhea | Fundus | 5.8 |
| 47 | B | Dysmenorrhea | Posterior wall of uterine body and fundus | 102.5 |
| 48 | B | Menorrhagia | posterior wall of uterine body and fundus | 100.9 |
| 49 | B | Dysmenorrhea | Anterior wall of uterine body | 117.1 |
| 50 | B | Dysmenorrhea | Anterior wall of uterine body | 53.0 |
| 51 | B | Menorrhagia | Posterior wall of uterine body | 151.6 |
| 52 | B | Dysmenorrhea | Uterine body and fundus | 136.1 |
| 53 | B | Dysmenorrhea | Posterior wall of uterine body | 34.3 |
| 54 | B | Dysmenorrhea | Fundus | 35.6 |
| 55 | B | Menorrhagia | Fundus | 71.3 |
| 56 | B | Dysmenorrhea | Posterior wall of uterine body | 306.6 |
| 57 | B | Dysmenorrhea | Anterior wall of uterine body | 237.5 |
| 58 | B | Dysmenorrhea | Posterior wall of uterine body | 149.1 |
| 59 | B | Menorrhagia | Fundus | 277.1 |
| 60 | B | Dysmenorrhea | Uterine body | 295.1 |
| 61 | B | Dysmenorrhea | Posterior wall of uterine body | 66.8 |
| 62 | B | Dysmenorrhea | Anterior wall of uterine body | 16.3 |
| 63 | B | Dysmenorrhea | Posterior wall of uterine body | 60.7 |
| 64 | B | Dysmenorrhea | Posterior wall of uterine body | 13.5 |
| 65 | B | Dysmenorrhea | Posterior wall of uterine body | 45.6 |
| 66 | B | Dysmenorrhea | Posterior wall of uterine body | 39.7 |

HIFU = high-intensity focused ultrasound

*Location and volume of uterine adenomyosis were assessed by using magnetic resonance imaging.
