# Supplementary material for: Optimized treatment parameter by computer simulation for high-intensity focused ultrasound treatment of uterine adenomyosis: Short-term and long-term results
Source: PLoS One. 2024 Mar 28;19(3):e0301193. doi: 10.1371/journal.pone.0301193 (PMC10977802; doi:10.1371/journal.pone.0301193)
Supplement: S3 Table — (DOCX) [file pone.0301193.s007.docx]

**S3 Table. Factors impacting on the qualitative HIFU treatment outcomes.**

|  | **Univariable analysis** | | | | **Multivariable analysis** | | | |
| --- | --- | --- | --- | --- | --- | --- | --- | --- |
| **DII** | **Odds ratio** | **95% CI** | | ***P value*** | **Odds ratio** | **95% CI** | | ***P value*** |
| Age, y | 1.114 | 0.964 | 1.288 | 0.1406 |  |  |  |  |
| Body mass index, kg/m^2^ | 1.202 | 0.977 | 1.480 | 0.0816* | 1.243 | 1.010 | 1.528 | 0.0398** |
| Volume of uterus, cm^3^ | 1.003 | 0.999 | 1.007 | 0.1622 |  |  |  |  |
| Volume of adenomyosis, cm^3^ | 1.002 | 0.997 | 1.007 | 0.4398 |  |  |  |  |
| Treatment time, min | 1.012 | 0.994 | 1.031 | 0.1946 |  |  |  |  |
| Sonication time, min | 1.027 | 0.993 | 1.063 | 0.1220 |  |  |  |  |
| Acoustic power, W | 1.008 | 0.996 | 1.020 | 0.2070 |  |  |  |  |
| Coexisting uterine myoma (yes/no) | 1.179 | 0.238 | 5.833 | 0.8385 |  |  |  |  |
| Abdominal surgical scar  (yes/no) | 1.152 | 0.318 | 4.181 | 0.8269 |  |  |  |  |
| Pain related to HIFU treatment (yes/no) | 0.793 | 0.521 | 1.209 | 0.2781 |  |  |  |  |
| HIFU treatment parameter group | 3.689 | 1.189 | 11.447 | 0.0246** | 4.418 | 1.402 | 13.922 | 0.0121** |
| Epidural anesthesia  (yes/no) | 2.534 | 0.798 | 8.049 | 0.1134 |  |  |  |  |
| **Dysmenorrhea score** | **Odds ratio** | **95% CI** |  | ***P value*** | **Odds ratio** | **95% CI** |  | ***P value*** |
| Age, y | 1.113 | 0.990 | 1.252 | 0.0734* |  |  |  |  |
| Body mass index, kg/m^2^ | 1.126 | 0.959 | 1.321 | 0.1450 |  |  |  |  |
| Volume of uterus, cm^3^ | 1.002 | 0.999 | 1.005 | 0.1752 |  |  |  |  |
| Volume of adenomyosis, cm^3^ | 1.000 | 0.996 | 1.004 | 0.8178 |  |  |  |  |
| Treatment time, min | 0.994 | 0.980 | 1.009 | 0.4242 |  |  |  |  |
| Sonication time, min | 0.993 | 0.967 | 1.021 | 0.6227 |  |  |  |  |
| Acoustic power, W | 1.003 | 0.994 | 1.013 | 0.4807 |  |  |  |  |
| Coexisting uterine myoma (yes/no) | 2.104 | 0.549 | 8.065 | 0.2714 |  |  |  |  |
| Abdominal surgical scar  (yes/no) | 0.963 | 0.314 | 2.950 | 0.9462 |  |  |  |  |
| Pain related to HIFU treatment (yes/no) | 0.859 | 0.614 | 1.203 | 0.3692 |  |  |  |  |
| HIFU treatment parameter group | 1.887 | 0.678 | 5.257 | 0.2184 |  |  |  |  |
| Epidural anesthesia  (yes/no) | 2.426 | 0.931 | 6.321 | 0.0691* |  |  |  |  |
| **Menorrhagia score** | **Odds ratio** | **95% CI** |  | ***P value*** | **Odds ratio** | **95% CI** |  | ***P value*** |
| Age, y | 1.019 | 0.905 | 1.147 | 0.7506 |  |  |  |  |
| Body mass index, kg/m^2^ | 1.143 | 0.971 | 1.346 | 0.1062 |  |  |  |  |
| Volume of uterus, cm^3^ | 1.001 | 0.998 | 1.004 | 0.3930 |  |  |  |  |
| Volume of adenomyosis, cm^3^ | 1.001 | 0.997 | 1.005 | 0.5060 |  |  |  |  |
| Treatment time, min | 0.996 | 0.981 | 1.011 | 0.5891 |  |  |  |  |
| Sonication time, min | 1.004 | 0.977 | 1.031 | 0.7839 |  |  |  |  |
| Acoustic power, W | 1.01 | 1.001 | 1.02 | 0.0387** | 1.012 | 1.003 | 1.022 | 0.0119** |
| Coexisting uterine myoma (yes/no) | 1.483 | 0.389 | 5.650 | 0.5566 |  |  |  |  |
| Abdominal surgical scar  (yes/no) | 0.855 | 0.282 | 2.597 | 0.7780 |  |  |  |  |
| Pain related to HIFU treatment (yes/no) | 0.631 | 0.453 | 0.879 | 0.0074** | 0.601 | 0.435 | 0.830 | 0.0025** |
| HIFU treatment parameter group | 2.524 | 0.916 | 6.960 | 0.0725* |  |  |  |  |
| Epidural anesthesia  (yes/no) | 2.348 | 0.883 | 6.243 | 0.0856* |  |  |  |  |
| **UFS-QOL** | **Odds ratio** | **95% CI** |  | ***P value*** | **Odds ratio** | **95% CI** |  | ***P value*** |
| Age, y | -0.388 | -1.653 | 0.877 | 0.5419 |  |  |  |  |
| Body mass index, kg/m^2^ | -1.032 | -2.736 | 0.673 | 0.2311 |  |  |  |  |
| Volume of uterus, cm^3^ | -0.024 | -0.054 | 0.007 | 0.1277 |  |  |  |  |
| Volume of adenomyosis, cm^3^ | -0.016 | -0.058 | 0.025 | 0.4363 |  |  |  |  |
| Treatment time, min | 0.037 | -0.119 | 0.194 | 0.6358 |  |  |  |  |
| Sonication time, min | 0.031 | -0.261 | 0.323 | 0.8321 |  |  |  |  |
| Acoustic power, W | -0.033 | -0.136 | 0.069 | 0.5190 |  |  |  |  |
| Coexisting uterine myoma (yes/no) | -8.634 | -22.892 | 5.624 | 0.2307 |  |  |  |  |
| Abdominal surgical scar  (yes/no) | -3.454 | -15.194 | 8.286 | 0.5587 |  |  |  |  |
| Pain related to HIFU treatment (yes/no) | 1.255 | -2.358 | 4.868 | 0.4900 |  |  |  |  |
| HIFU treatment parameter group | -1.645 | -12.607 | 9.317 | 0.7652 |  |  |  |  |
| Epidural anesthesia  (yes/no) | -4.858 | -14.896 | 5.181 | 0.3372 |  |  |  |  |
| **SF-36v2** | **Odds ratio** | **95% CI** |  | ***P value*** | **Odds ratio** | **95% CI** |  | ***P value*** |
| Age, y | -0.002 | -0.446 | 0.442 | 0.9927 |  |  |  |  |
| Body mass index, kg/m^2^ | 0.189 | -0.420 | 0.797 | 0.5381 |  |  |  |  |
| Volume of uterus, cm^3^ | 0.006 | -0.004 | 0.017 | 0.2417 |  |  |  |  |
| Volume of adenomyosis, cm^3^ | 0.007 | -0.008 | 0.022 | 0.3377 |  |  |  |  |
| Treatment time, min | 0.027 | -0.028 | 0.081 | 0.3293 |  |  |  |  |
| Sonication time, min | 0.055 | -0.046 | 0.155 | 0.2814 |  |  |  |  |
| Acoustic power, W | 0.018 | -0.018 | 0.054 | 0.3262 |  |  |  |  |
| Coexisting uterine myoma (yes/no) | 3.137 | -1.754 | 8.029 | 0.2047 |  |  |  |  |
| Abdominal surgical scar  (yes/no) | 1.771 | -2.351 | 5.893 | 0.3938 |  |  |  |  |
| Pain related to HIFU treatment (yes/no) | 0.341 | -0.907 | 1.589 | 0.5865 |  |  |  |  |
| HIFU treatment parameter group | -1.897 | -5.740 | 1.947 | 0.3279 |  |  |  |  |
| Epidural anesthesia  (yes/no) | -3.144 | -6.638 | 0.349 | 0.0768* |  |  |  |  |
| **SSS** | **Odds ratio** | **95% CI** |  | ***P value*** | **Odds ratio** | **95% CI** |  | ***P value*** |
| Age, y | -0.330 | -1.122 | 0.462 | 0.4082 |  |  |  |  |
| Body mass index, kg/m^2^ | -1.019 | -2.090 | 0.051 | 0.0616* |  |  |  |  |
| Volume of uterus, cm^3^ | -0.020 | -0.039 | -0.001 | 0.0372** | -0.020 | -0.039 | -0.001 | 0.0372** |
| Volume of adenomyosis, cm^3^ | -0.020 | -0.047 | 0.006 | 0.1238 |  |  |  |  |
| Treatment time, min | 0.057 | -0.043 | 0.157 | 0.2615 |  |  |  |  |
| Sonication time, min | 0.077 | -0.113 | 0.267 | 0.4211 |  |  |  |  |
| Acoustic power, W | -0.032 | -0.097 | 0.033 | 0.3231 |  |  |  |  |
| Coexisting uterine myoma (yes/no) | -3.239 | -12.173 | 5.695 | 0.4714 |  |  |  |  |
| Abdominal surgical scar  (yes/no) | -1.555 | -9.012 | 5.901 | 0.6782 |  |  |  |  |
| Pain related to HIFU treatment (yes/no) | 1.437 | -0.782 | 3.657 | 0.2004 |  |  |  |  |
| HIFU treatment parameter group | -1.540 | -8.498 | 5.418 | 0.6598 |  |  |  |  |
| Epidural anesthesia  (yes/no) | -2.926 | -9.312 | 3.460 | 0.3633 |  |  |  |  |

Values are presented as medians (interquartile ranges), otherwise indicated. HIFU = high-intensity focused ultrasound, CI = confidence interval, DII = dysmenorrhea improvement index, UFS-QOL = uterine fibroid symptom and quality of life questionnaire, SF-36v2 = 36-item short-form health survey version 2, SSS = symptom severity score.

**P* < 0.050, ***P* < 0.050
