# Supplementary material for: Optimized treatment parameter by computer simulation for high-intensity focused ultrasound treatment of uterine adenomyosis: Short-term and long-term results
Source: PLoS One. 2024 Mar 28;19(3):e0301193. doi: 10.1371/journal.pone.0301193 (PMC10977802; doi:10.1371/journal.pone.0301193)
Supplement: S4 Table — (DOCX) [file pone.0301193.s008.docx]

**S4 Table. Qualitative measurement of treatment efficacy between the modes of anesthesia**

|  |  | **MAC**  **(n = 36)** | **EA**  **(n = 30)** | **Odds ratio or Ls means**^†^ | **95% CI** | ***P* value** |
| --- | --- | --- | --- | --- | --- | --- |
| Clinically effective DII^††^ | 1 m F/U | 83.3 (30/36) | 83.3 (25/30) | 1.23 | 0.28, 5.44 | 0.785 |
|  | 3 m F/U | 88.9 (32/36) | 100.0 (30/30) |  |  |  |
|  | 1 y F/U | 72.7 (24/33) | 92.0 (23/25) |  |  |  |
|  | 3 y F/U | 66.7 (16/24) | 77.8 (14/18) |  |  |  |
| Dysmenorrhea score | Screening | 4 (4–5) | 5 (4–5) | 2.29 | 0.68, 7.67 | 0.175 |
|  | 1 m F/U | 3 (2–4) | 2.5 (2–3) |  |  |  |
|  | 3 m F/U | 2 (2–3) | 2 (1–2) |  |  |  |
|  | 1 y F/U | 2 (2–3) | 2 (2–3) |  |  |  |
|  | 3 y F/U | 2.5 (1–3) | 2.5 (2–4) |  |  |  |
| Menorrhagia score | Screening | 4 (4–5) | 4.5 (3–5) | 1.66 | 0.49, 5.60 | 0.407 |
|  | 1 m F/U | 3 (1–3) | 1 (1–3) |  |  |  |
|  | 3 m F/U | 2 (1.5–3) | 1.5 (1–3) |  |  |  |
|  | 1 y F/U | 2 (1–3) | 1 (1–2) |  |  |  |
|  | 3 y F/U | 2 (1–3) | 2.5 (1–3) |  |  |  |
| UFS-QOL | Screening | 122.5 (105–135) | 119 (90–138) | -6.28 | -19.06, 6.50 | 0.330 |
|  | 1 m F/U | 85 (71–112) | 82 (63–111) |  |  |  |
|  | 3 m F/U | 78.5 (58.5–93) | 65 (53–83) |  |  |  |
| SF-36v2 | Screening | 113 (105–118) | 110.5 (104–117) | -3.27 | -7.67, 1.13 | 0.143 |
|  | 1 m F/U | 115.5 (111–118.5) | 112.5 (106–120) |  |  |  |
|  | 3 m F/U | 118.5 (113–124.5) | 114 (109–120) |  |  |  |
| SSS | Screening | 56 (44–70.5) | 59 (47–72) | -3.25 | -11.30, 4.81 | 0.424 |
|  | 1 m F/U | 31 (23.5–50) | 32.5 (25–47) |  |  |  |
|  | 3 m F/U | 29.5 (22–41) | 25 (16–34) |  |  |  |

Values are presented as medians (quartiles 1–3), otherwise indicated. MAC = monitored anesthesia care, EA = epidural anesthesia, CI = confidence interval, DII = dysmenorrhea improvement index, UFS-QOL = uterine fibroid symptom and quality of life questionnaire, SF-36v2 = 36-item short-form health survey version 2, SSS = symptom severity score.

^†^Odds ratios were obtained by using generalized linear mixed models for clinically effective DII, dysmenorrhea score, and menorrhagia score. Ls means were obtained by using mixed models for UFS-QOL, SF-36v2, and SSS.

^††^Number are percentages with proportions in parentheses.

**P* < 0.050
