# Supplementary material for: Optimized treatment parameter by computer simulation for high-intensity focused ultrasound treatment of uterine adenomyosis: Short-term and long-term results
Source: PLoS One. 2024 Mar 28;19(3):e0301193. doi: 10.1371/journal.pone.0301193 (PMC10977802; doi:10.1371/journal.pone.0301193)
Supplement: S5 Table — (DOCX) [file pone.0301193.s009.docx]

**S5 Table. Factors impacting on the quantitative HIFU treatment outcomes.**

|  | **Univariable analysis** | | | | **Multivariable analysis** | | | |
| --- | --- | --- | --- | --- | --- | --- | --- | --- |
| **Volume of adenomyosis** | **Odds ratio** | **95% CI** | | ***P value*** | **Odds ratio** | **95% CI** | | ***P value*** |
| Age, y | 0.526 | -0.742 | 1.795 | 0.410 |  |  |  |  |
| Body mass index, kg/m^2^ | -0.896 | -2.681 | 0.889 | 0.320 |  |  |  |  |
| Volume of uterus, cm^3^ | -0.008 | -0.069 | 0.052 | 0.784 |  |  |  |  |
| Volume of adenomyosis, cm^3^ |  |  |  |  |  |  |  |  |
| Treatment time, min | -0.073 | -0.225 | 0.079 | 0.340 |  |  |  |  |
| Sonication time, min | -0.035 | -0.320 | 0.249 | 0.805 |  |  |  |  |
| Acoustic power, W | -0.091 | -0.195 | 0.014 | 0.087 |  |  |  |  |
| Coexisting uterine myoma (yes/no) | 3.411 | -10.567 | 17.389 | 0.628 |  |  |  |  |
| Abdominal surgical scar  (yes/no) | 0.121 | -11.632 | 11.874 | 0.984 |  |  |  |  |
| Pain related to HIFU treatment (yes/no) | 0.001 | -3.490 | 3.491 | 1.000 |  |  |  |  |
| HIFU treatment parameter group | -3.380 | -14.115 | 7.355 | 0.532 |  |  |  |  |
| Epidural anesthesia  (yes/no) | 0.234 | -9.707 | 10.175 | 0.963 |  |  |  |  |
| **NPV** | **Odds ratio** | **95% CI** |  | ***P value*** | **Odds ratio** | **95% CI** |  | ***P value*** |
| Age, y | 5.355 | 0.691 | 10.019 | 0.0251 |  |  |  |  |
| Body mass index, kg/m^2^ | 7.999 | 1.651 | 14.346 | 0.0143 |  |  |  |  |
| Volume of uterus, cm^3^ | 0.413 | 0.351 | 0.475 | <0.0001** | 0.106 | 0.019 | 0.192 | 0.0175** |
| Volume of adenomyosis, cm^3^ | 0.605 | 0.544 | 0.667 | <0.0001** | 0.481 | 0.364 | 0.599 | <0.0001** |
| Treatment time, min | -0.141 | -0.742 | 0.461 | 0.6414 |  |  |  |  |
| Sonication time, min | -0.524 | -1.631 | 0.583 | 0.3479 |  |  |  |  |
| Acoustic power, W | 0.425 | 0.043 | 0.807 | 0.0300 |  |  |  |  |
| Coexisting uterine myoma (yes/no) | -26.383 | -80.372 | 27.605 | 0.3326 |  |  |  |  |
| Abdominal surgical scar  (yes/no) | -24.065 | -69.171 | 21.042 | 0.2905 |  |  |  |  |
| Pain related to HIFU treatment (yes/no) | 4.512 | -8.978 | 18.001 | 0.5064 |  |  |  |  |
| HIFU treatment parameter group | 22.200 | -19.870 | 64.270 | 0.2958 |  |  |  |  |
| Epidural anesthesia  (yes/no) | 19.552 | -19.307 | 58.411 | 0.3186 |  |  |  |  |
| **NPVR** | **Odds ratio** | **95% CI** |  | ***P value*** | **Odds ratio** | **95% CI** |  | ***P value*** |
| Age, y | 1.484 | -0.441 | 3.409 | 0.1284 |  |  |  |  |
| Body mass index, kg/m^2^ | 0.398 | -2.289 | 3.084 | 0.7685 |  |  |  |  |
| Volume of uterus, cm^3^ | 0.075 | 0.030 | 0.120 | 0.0014** | 0.082 | 0.047 | 0.116 | <0.0001** |
| Volume of adenomyosis, cm^3^ | 0.083 | 0.021 | 0.146 | 0.0096** |  |  |  |  |
| Treatment time, min | -0.184 | -0.423 | 0.055 | 0.1289 |  |  |  |  |
| Sonication time, min | -0.027 | -0.477 | 0.424 | 0.9061 |  |  |  |  |
| Acoustic power, W | 0.013 | -0.147 | 0.173 | 0.8715 |  |  |  |  |
| Coexisting uterine myoma (yes/no) | -8.196 | -30.077 | 13.685 | 0.4570 |  |  |  |  |
| Abdominal surgical scar  (yes/no) | -5.567 | -23.900 | 12.767 | 0.5463 |  |  |  |  |
| Pain related to HIFU treatment (yes/no) | -3.196 | -8.607 | 2.214 | 0.2423 |  |  |  |  |
| HIFU treatment parameter group | 26.920 | 11.148 | 42.693 | 0.0011** |  |  |  |  |
| Epidural anesthesia  (yes/no) | 36.746 | 23.853 | 49.638 | <0.0001** | 38.305 | 27.140 | 49.471 | <0.0001** |
| **AVSR** | **Odds ratio** | **95% CI** |  | ***P value*** | **Odds ratio** | **95% CI** |  | ***P value*** |
| Age, y | -0.208 | -1.751 | 1.336 | 0.7889 |  |  |  |  |
| Body mass index, kg/m^2^ | -0.740 | -2.849 | 1.370 | 0.4861 |  |  |  |  |
| Volume of uterus, cm^3^ | -0.011 | -0.049 | 0.028 | 0.5846 |  |  |  |  |
| Volume of adenomyosis, cm^3^ | -0.046 | -0.096 | 0.005 | 0.0754* | -0.047 | -0.093 | 0.000 | 0.0493** |
| Treatment time, min | 0.098 | -0.092 | 0.289 | 0.3054 |  |  |  |  |
| Sonication time, min | 0.221 | -0.130 | 0.571 | 0.2134 |  |  |  |  |
| Acoustic power, W | 0.003 | -0.123 | 0.129 | 0.9640 |  |  |  |  |
| Coexisting uterine myoma (yes/no) | 1.323 | -15.982 | 18.628 | 0.8791 |  |  |  |  |
| Abdominal surgical scar  (yes/no) | 9.317 | -4.976 | 23.610 | 0.1975 |  |  |  |  |
| Pain related to HIFU treatment (yes/no) | -1.203 | -5.500 | 3.095 | 0.5781 |  |  |  |  |
| HIFU treatment parameter group | 21.611 | 9.233 | 33.989 | 0.0009** | 21.735 | 9.634 | 33.835 | 0.0006** |
| Epidural anesthesia  (yes/no) | 13.843 | 1.869 | 25.817 | 0.0242 |  |  |  |  |

Values are presented as medians (interquartile ranges), otherwise indicated. HIFU = high-intensity focused ultrasound, CI = confidence interval, NPV = nonperfused volume, NPVR = nonperfused volume ratio, AVSR = adenomyosis volume shrinkage ratio.

**P* < 0.050, ***P* < 0.050
