# Supplementary material for: Optimized treatment parameter by computer simulation for high-intensity focused ultrasound treatment of uterine adenomyosis: Short-term and long-term results
Source: PLoS One. 2024 Mar 28;19(3):e0301193. doi: 10.1371/journal.pone.0301193 (PMC10977802; doi:10.1371/journal.pone.0301193)
Supplement: S6 Table — (DOCX) [file pone.0301193.s010.docx]

**S6 Table. Quantitative measurement of treatment efficacy between the modes of anesthesia**

|  |  | **MAC**  **(n = 36)** | **EA**  **(n = 30)** | **Ls means** | **95% CI** | ***P* value** |
| --- | --- | --- | --- | --- | --- | --- |
| Volume of adenomyosis, cm^3^ | Immediate | 68.1 (37.9–138.1) | 82.5 (39.7–165.6) | 3.32 | -9.17, 15.81 | 0.597 |
|  | 1 m F/U | 66.1 (29.0–124.6) | 70.2 (24.7–154.9) |  |  |  |
|  | 3 m F/U | 55.9 (23.0–103.6) | 35 (12.0–149.1) |  |  |  |
| Nonperfused volume, cm^3^ | Immediate | 22.2 (10.0–57.3) | 64.8 (23.3–149.1) | 11.32 | -37.62, 60.26 | 0.646 |
|  | 1 m F/U | 9.2 (0.8–50.7) | 43.4 (14.3–105.9) |  |  |  |
|  | 3 m F/U | 2.8 (0.0–16.6) | 16.2 (3.2–44.6) |  |  |  |
| NPVR, % | Immediate | 41.4 (19.2–74.9) | 85.4 (63.4–91.8) | 34.17 | 17.93, 50.41 | <0.001* |
|  | 1 m F/U | 23.2 (1.8–73.5) | 76.7 (64.2–96.2) |  |  |  |
|  | 3 m F/U | 6.4 (0.0–46.1) | 67.9 (28.6–86.7) |  |  |  |
| AVSR, % | 1 m F/U | 23.9 (1.3–37.7) | 18.5 (6.4–42.2) | 2.88 | -11.53, 17.29 | 0.691 |
|  | 3 m F/U | 35.4 (19.8–47.6) | 58.5 (38.1–73.4) |  |  |  |

Values are presented as medians (quartile 1–3). MAC = monitored anesthesia care, EA = epidural anesthesia, CI = confidence interval, NPVR = nonperfused volume ratio, AVSR = adenomyosis volume shrinkage ratio
