# Supplementary material for: Optimized treatment parameter by computer simulation for high-intensity focused ultrasound treatment of uterine adenomyosis: Short-term and long-term results
Source: PLoS One. 2024 Mar 28;19(3):e0301193. doi: 10.1371/journal.pone.0301193 (PMC10977802; doi:10.1371/journal.pone.0301193)
Supplement: S7 Table — (DOCX) [file pone.0301193.s011.docx]

**S7 Table. SIR classification and distribution of adverse events**

| **SIR classification** | **Adverse events** | **MAC**  **(n = 36)** | **EA**  **(n = 30)** | ***P value*** |
| --- | --- | --- | --- | --- |
| Mild | Lower abdominal pain | 25 | 9 | 0.003 |
|  | Vaginal discharge | 12 | 1 | 0.004 |
|  | Hip pain | 2 | 8 | 0.035 |
|  | Nausea or vomiting | 7 | 1 | 0.063 |
|  | Lower limb paresthesia | 1 | 4 | 0.169 |
|  | Dysuria |  | 1 | 0.455 |
| Moderate | Burn injury |  | 1 | 0.455 |
| Severe | Lumbosacral plexus injury |  | 1 | 0.455 |
|  | Rectal wall injury | 1 |  | > 0.999 |

SIR = Society of Interventional Radiology, MAC = monitored anesthesia care, EA = epidural anesthesia.
