# Supplementary material for: Optimized treatment parameter by computer simulation for high-intensity focused ultrasound treatment of uterine adenomyosis: Short-term and long-term results
Source: PLoS One. 2024 Mar 28;19(3):e0301193. doi: 10.1371/journal.pone.0301193 (PMC10977802; doi:10.1371/journal.pone.0301193)
Supplement: S2 File — (DOC) [file pone.0301193.s013.doc]

Protocol No. APM-03 Version 1.10/Date 20180103


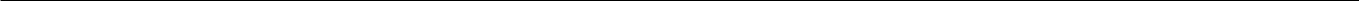


A prospective, multicenter, single-arm confirmatory clinical trial to evaluate the safety and efficacy of ‘ALPIUS 900’ (US-guided HIFU System, ultrasound-guided, high-intensity focused ultrasound surgery device) in patients with adenomyosis

Protocol No.: APM-03

Version No.: 1.10

Date: January 3, 2018

ALPINION Medical Systems Co., Ltd.

ALPINION Medical Systems Co., Ltd. 1/45


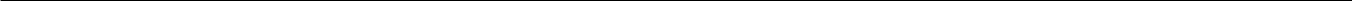

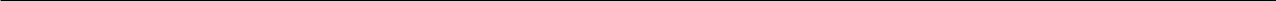


Protocol No. APM-03 Version 1.10/Date 20180103


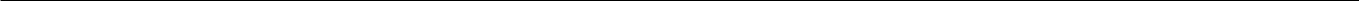


**Signature**

**Principal Investigator**

By signing below, I hereby acknowledge that I have read, reviewed and understood all information on this investigational medical device. In addition, I have read and reviewed this clinical trial protocol, and hereby agree to proceed with the clinical trial accordingly. I will conduct this clinical trial in accordance with the ICH Good Clinical Practice (GCP) standards and all applicable regulations, and fulfill my main duties as a researcher in accordance with the Declaration of Helsinki and the ethical standards of the Institutional Review Board (IRB).

Name of Clinical Trial Site


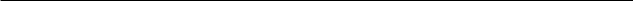


Principal Investigator Signature Date


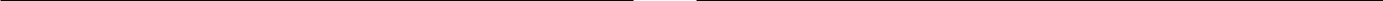


ALPINION Medical Systems Co., Ltd. 2/45


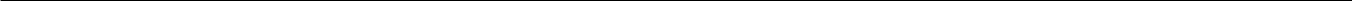

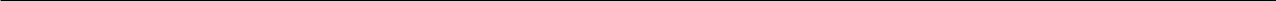


Protocol No. APM-03 Version 1.10/Date 20180103


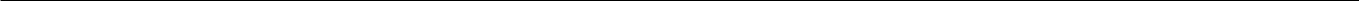


***< Protocol Synopsis >***

***
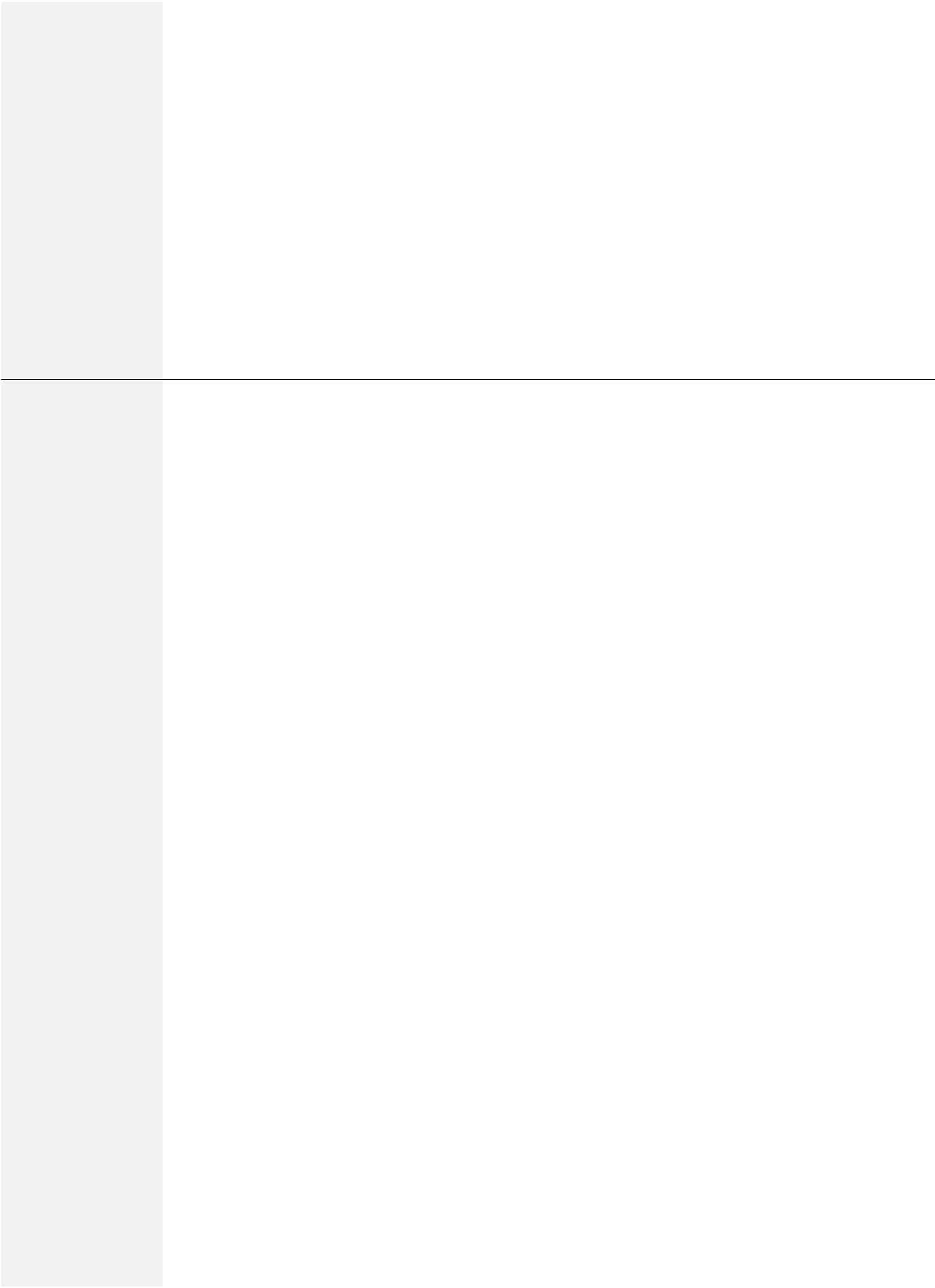
***

| Title | A prospective, multicenter, single-arm confirmatory clinical trial to evaluate the safety and efficacy of ‘ALPIUS 900’ (US-guided HIFU System, ultrasound-guided, high-intensity focused ultrasound surgery device) in patients with adenomyosis |
| --- | --- |
|  |  |
|  |  |
|  |  |
|  | This is a clinical trial for confirming the efficacy and safety of non-invasive adenomyosis ablation in symptomatic adenomyosis using ‘ALPIUS 900 (US-guided HIFU System)’, an ultrasound-guided high-intensity focused ultrasound surgical device by ALPINION Medical Systems Co., Ltd., which has been approved for manufacturing for the indication uterine fibroids. |
| Objective |  |
|  |  |
|  | (For MFDS approval) |
|  |  |
|  |  |
| Study Site | Seoul National University Hospital / Prof. Jae Yeong Lee (Department of Radiology) |
| /Principal Investigator | Konyang University Hospital / Prof. Cheol Joong Kim (Department of Obstetrics and Gynecology) |
| Investigational | Seoul National University Hospital / Dong Hyeok Park, Radiologist, Department of Radiology |
| Medical Device | Konyang University Hospital / Da Mi Choi, Study Coordinator (Department of Obstetrics and Gynecology) |
| Manager |  |
| Sponsor | ALPINION Medical Systems Co., Ltd., CEO: Yeong Choon Choi |
|  | 1st, 6th FL., Verdi Tower, Guro Digital-ro 26 gil 72, Guro-gu, Seoul |
|  |  |
| Monitor | Synex Co., Ltd., CEO Yeong Kim |
|  | 10th FL., ASEA TOWER, 430 Nonhyeon-ro, Gangnam-gu, Seoul (06223) |
|  |  |
| Target Disease | Patients requiring cauterization of adenomyosis |
| /Subjects |  |
|  |  |
| Number of Subjects | 80 subjects (taking 10% dropout rate into account) |
|  |  |
| Investigational Medical Device | ‘ALPIUS 900’ (Manufacturing Approval No. 14-3227) |
|  | Ultrasound-guided high-intensity focused ultrasound surgery device (US-guided HIFU) |
|  | This clinical trial was planned to evaluate the safety and efficacy of 'ALPIUS 900', a high-intensity focused ultrasound surgical device guided by ultrasound images, in symptomatic patients with adenomyosis. |
| Study Design |  |
|  | As a prospective, multicenter, single-arm clinical trial, it will be conducted with a total of 80 patients at two domestic institutions. |
|  |  |
|  |  |
|  | 1. Patients who are clinically diagnosed with adenomyosis after visiting the Department of Obstetrics and Gynecology will be referred to this clinical trial. |
|  |  |
|  | ☞ Patients who have received CT, MRI, or ultrasound images from the hospital where adenomyosis was first diagnosed, or a diagnostic image or report from the hospital where they are currently being treated can also be enrolled. |
| Study Method |  |
|  |  |
|  | 2. Those who voluntarily sign the consent form and satisfy all inclusion/exclusion criteria will be enrolled in this study and assigned a subject number. |
|  |  |
|  | 3. Subjects enrolled in the study schedule the procedure and receive the scheduled procedure on the day of the procedure. |
|  | Pre-procedure preparations and procedures follow the standard procedures of the institution. |

ALPINION Medical Systems Co., Ltd. 3/45


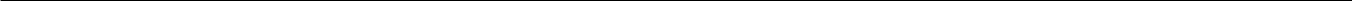

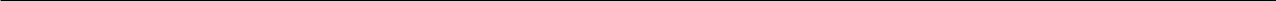


Protocol No. APM-03 Version 1.10/Date 20180103


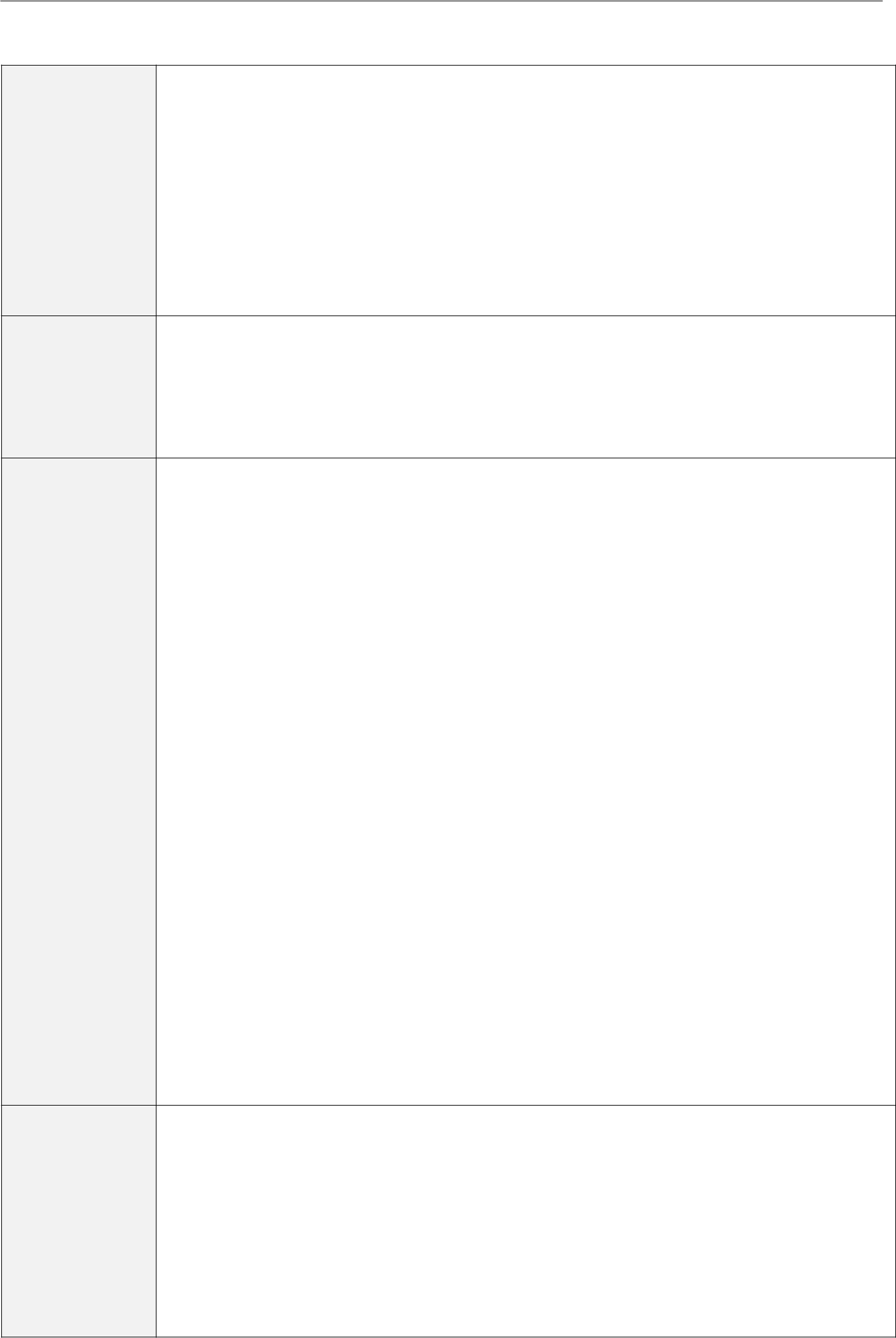


1. After the procedure, the operator acquires an image of the treatment area through ultrasound and MRI to check the planned volume and the range of the treatment volume, and observes for any adverse reactions including changes in the skin.
2. After 1 to 2 hours of monitoring in the recovery room, if there are no abnormalities, the patient can be discharged on the same day or the following day.
3. Efficacy and safety are evaluated at one month and three months after the procedure, and the clinical trial is terminated if there are no abnormalities in the subject's body.

The study is expected to take approximately 28 months, including approximately 25 months of subject enrollment after obtaining clinical trial protocol approval from the Ministry of Food and Drug Safety (MFDS) and 3 months of follow-up.

Clinical Trial Period

Even after the clinical trial is completed, it is expected to take approximately 3 months in addition for data processing, statistical analysis, clinical study report preparation and IRB approval.

Subjects can be enrolled in this clinical trial only if they meet all of the following inclusion criteria.

1. Adult female of at least 20 years of age
2. Pre-menopausal or peri-menopausal (FSH<40mIU/ml)
3. Adenomyosis is clinically diagnosed through MR or US imaging
4. Pain score on the Dysmenorrhea Score is at least 4 points

- Dysmenorrhea Score

1 = Not at all (no symptoms)

2 = A little bit (slight symptoms)

3 = Somewhat (few symptoms)

Inclusion Criteria  4 = A great deal (considerable symptoms)

5 = A very great deal (a lot of symptoms)

1. Have not received any other treatment for adenomyosis within 3 months before HIFU procedure
   - *In the case of hormone therapy, even within 3 months, subjects can be enrolled if five times the half-life of the drug has elapsed since the last administration.*
2. Those who voluntarily consent to the clinical trial and are willing to comply with the protocol
3. Those who agree to use a medically accepted method of contraception for the duration of the clinical trial.

** Medically accepted method of contraception: Physical contraceptive devices other than contraceptives through hormonal control such as condoms or installation of intrauterine contraceptive devices*

Subjects cannot be enrolled in this clinical trial if they meet any of the following exclusion criteria.

1. Presence of other pelvic diseases such as other malignant tumors, endometriosis, ovarian tumors, or acute pelvic disease

| Exclusion Criteria |  |
| --- | --- |
| 2. | Sarcoma is clinically suspected |
| 3. | Diffuse adenomyosis, in which adenomyosis has spread throughout the uterus, is clinically suspected |
|  |  |

ALPINION Medical Systems Co., Ltd. 4/45


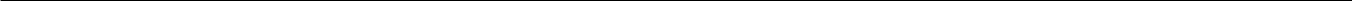

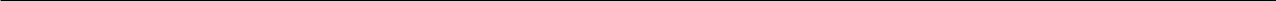


Protocol No. APM-03 Version 1.10/Date 20180103


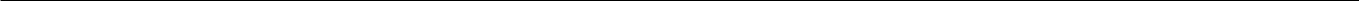


1. Positive result in pregnancy test or plans to get pregnant
2. Presence of a serious systemic disease
3. If hematocrit is less than 25%
4. There is an extensive abdominal scar in the area where the ultrasound light passes
   - - *However, if the investigator decides that HIFU treatment can be performed by applying a scar patch even if there is an extensive abdominal scar in the area where the ultrasound light passes, this exclusion criterion will not exclude the subject from the clinical trial.*
5. There is a scar or surgical clip in the passageway through which the high-intensity focused ultrasound passes
6. If the subject cannot lie down in a comfortable position
7. Contraindications to MRI (including those with claustrophobia)
8. Contraindications to MRI contrast agents
9. Contraindications to ultrasound contrast agents
10. When GFR (Glomerular filtration rate) is 30ml/min or less
11. In case communication is difficult
12. Patients who have participated in other clinical trials within the last 1 month
13. Other subjects deemed ineligible to participate in this clinical trial according to the judgment of the investigator
    - Record specific reasons in the case report form


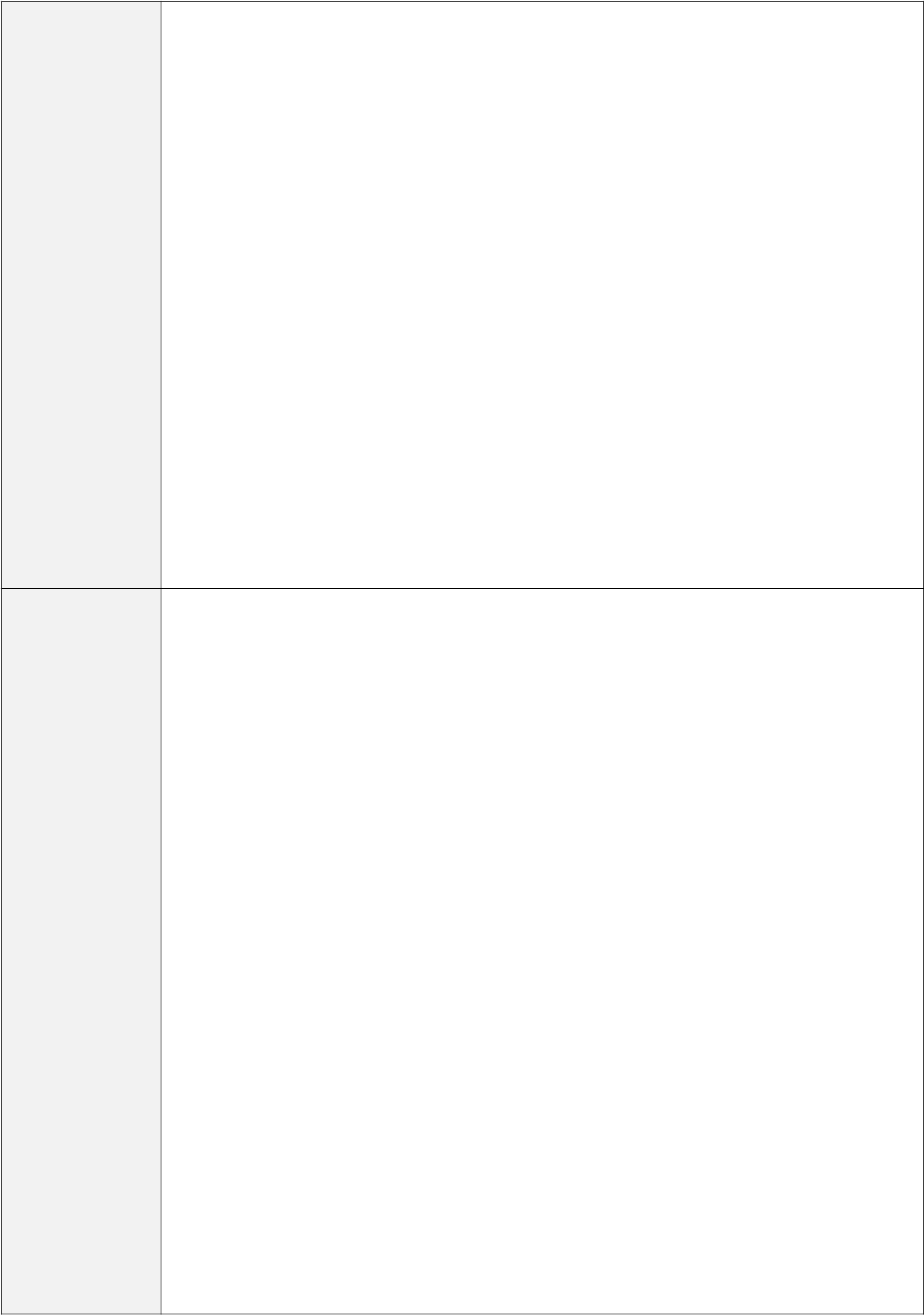


<Primary Efficacy Endpoint>

|  | ■ Percentage Improvement in Dysmenorrhea Relief (%) - 3 months after treatment | | |
| --- | --- | --- | --- |
|  | <Secondary Efficacy Endpoint> | | |
|  | ■ Percentage Improvement in Dysmenorrhea Relief (%) - 1 month after treatment | | |
| Efficacy | ■ Dysmenorrhea Score | | |
| Endpoint | ■ Menorrhagia Score | | |
|  | ■ Measurement of Quality of Life | | |
|  | - SF36v2 | | |
|  | - UFS-QoL | | |
|  | - SSS | | |
|  | ■ Uterus size(cm^3^) | | |
|  | ■ Satisfaction with the procedure | | |
| Safety | All adverse events that occurred to subjects during the clinical trial period | | |
| Endpoint |
|  |  |  |
|  | <Primary Efficacy Endpoint> | | |
|  | ■ Percentage Improvement in Dysmenorrhea Relief (%) | | |
| Efficacy | ● Definition of dysmenorrhea relief | | |
| Assessment Criteria |  | If the degree of menstrual pain relief corresponds to Minor, Partial, or Complete on the Dysmenorrhea Relief Score | |
|  |  |  | |
|  |  | Dysmenorrhea Relief Score |  |

ALPINION Medical Systems Co., Ltd. 5/45


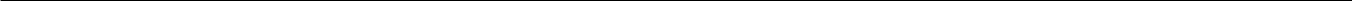

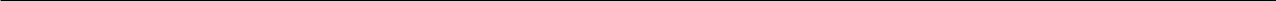


Protocol No. APM-03 Version 1.10/Date 20180103


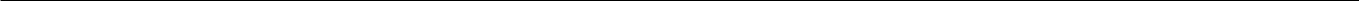


- Complete relief

② Partial relief

③ Minor relief

④ Ineffective


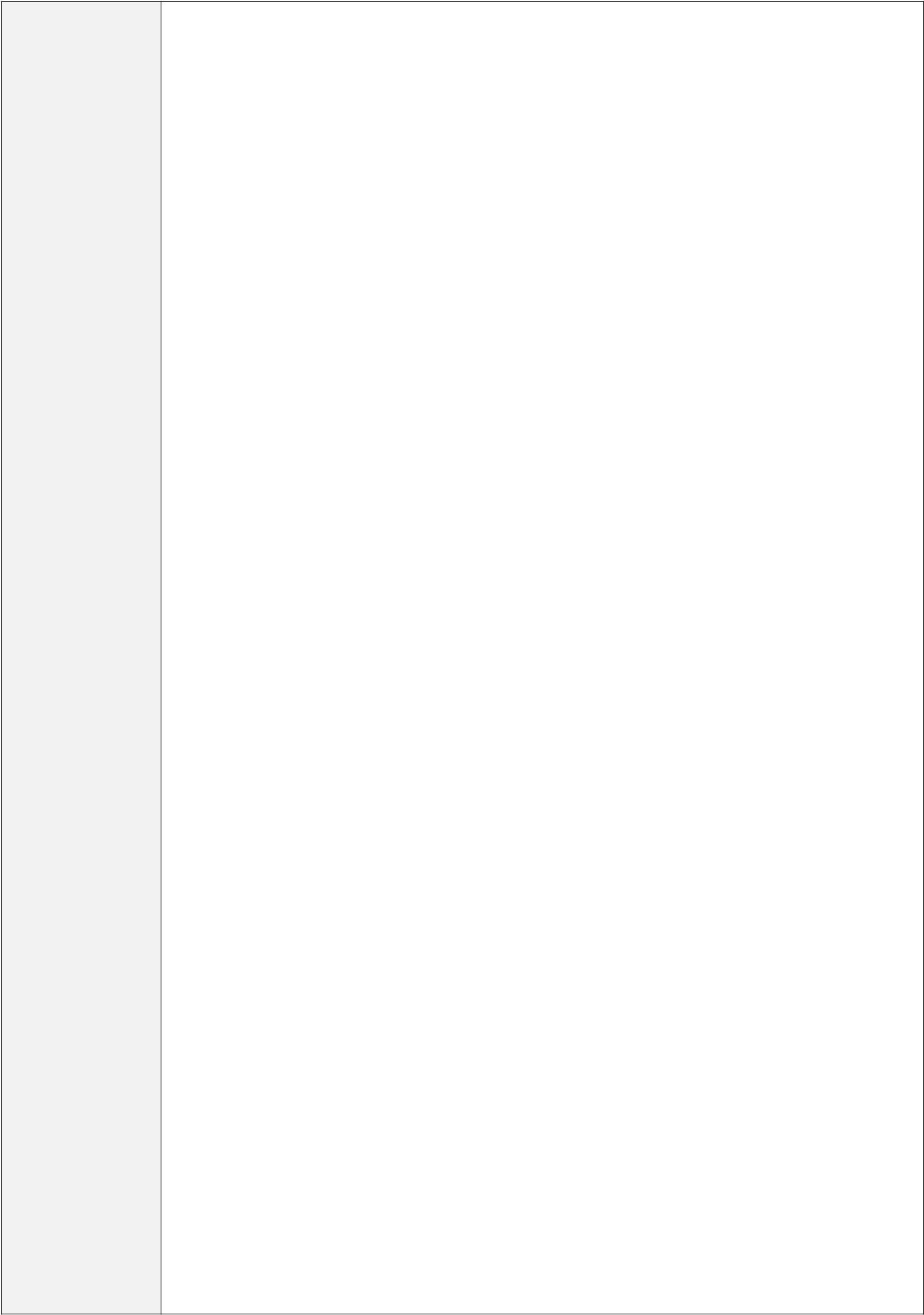


- - Exacerbated pain
- Definition of Percentage Improvement in Dysmenorrhea Relief

Proportion of subjects who met the definition of improvement in dysmenorrhea relief above at 3 months after the procedure

<Secondary Efficacy Endpoint>

■ Percentage Improvement in Dysmenorrhea Relief (%) - 1 month after treatment

Evaluate the Percentage Improvement in Dysmenorrhea Relief (%) 1 month after the procedure using the same method as that for the primary efficacy endpoint.

■ Dysmenorrhea Score

Subjects directly evaluate dysmenorrhea symptoms on a 5-point scale as shown below before and at 1 and 3 months after the procedure.

Dysmenorrhea Score

1 = Not at all (no symptoms)

1. = A little bit (slight symptoms)
2. = Somewhat (few symptoms)
3. = A great deal (considerable symptoms)
4. = A very great deal (a lot of symptoms)

■ Menorrhagia Score

Subjects directly evaluate menorrhagia symptoms on a 5-point scale as shown below before and at 1 and 3 months after the procedure.

Menorrhagia Score

1 = Not at all (no symptoms)

1. = A little bit (slight symptoms)
2. = Somewhat (few symptoms)
3. = A great deal (considerable symptoms)
4. = A very great deal (a lot of symptoms)

■ Measurement of Quality of Life

Using the questionnaire filled out by the subject, the changes below before and at 1 and 3 months after the procedure are compared and evaluated.

- SF36-v2

This is a questionnaire to evaluate the overall quality of life, and consists of 36 questions in 8 domains including physical functioning, role limitation-physical, bodily pain, general health, vitality, social functioning, role limitation-emotional, mental health, and other changes in health condition, etc.

ALPINION Medical Systems Co., Ltd. 6/45


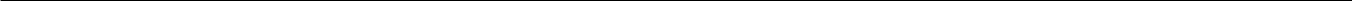

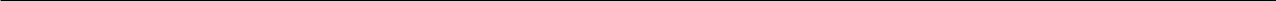


Protocol No. APM-03 Version 1.10/Date 20180103


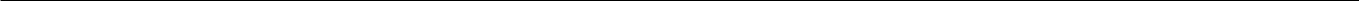


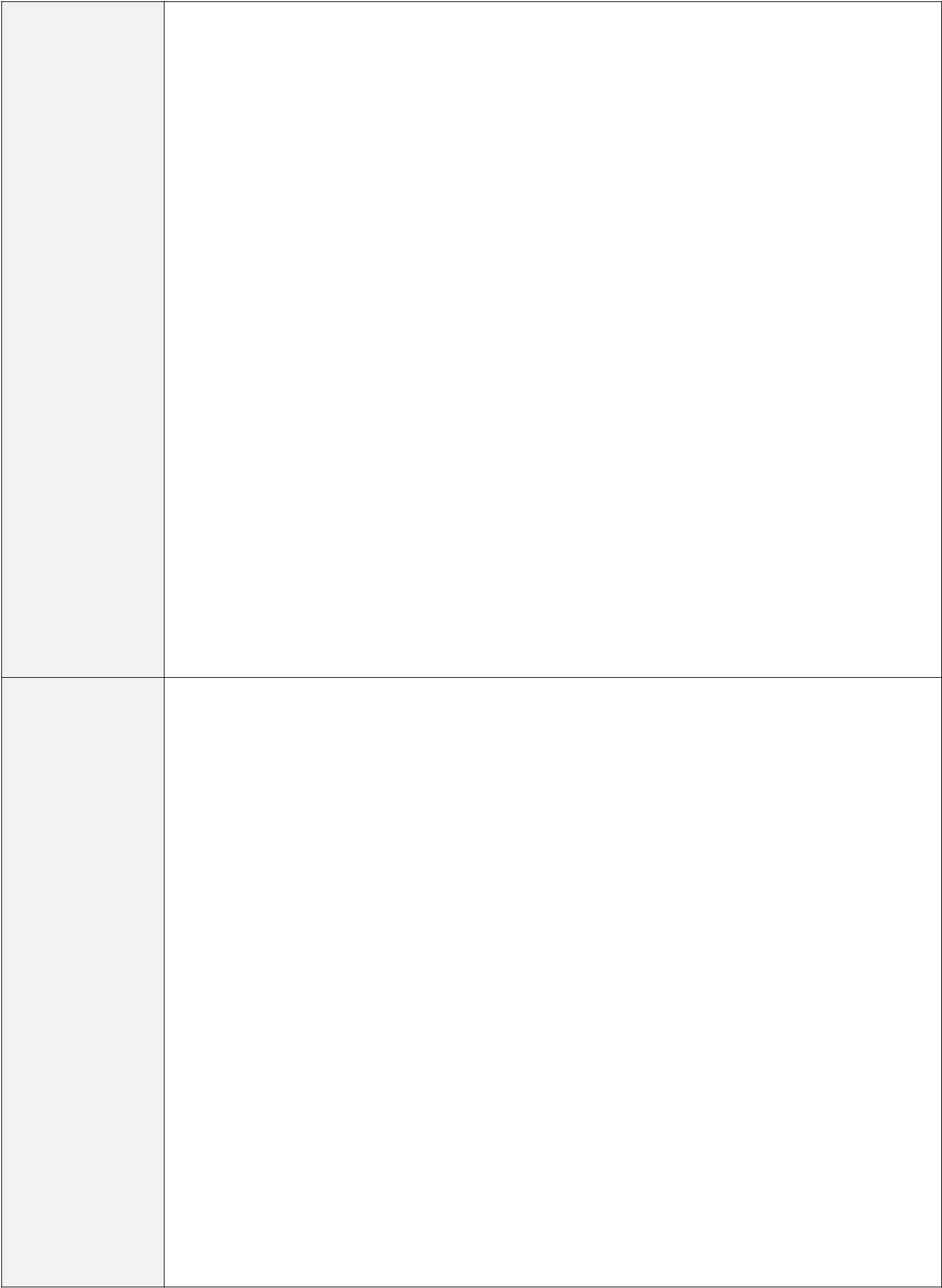


- UFS-QOL

As a questionnaire to evaluate the quality of life related to the symptoms of uterine fibroids, it consists of an 8-item Symptom Severity Score and a 29-item Quality of Life questionnaire.

- SSS (Symptom Severity Score)

This is included in the UFS-QOL questionnaire but the severity of symptoms due to uterine fibroids will be evaluated separately.

- Uterus size (cm^3^)

Compare the overall uterus size immediately after the procedure, at 1 month and 3 months after the procedure.

- Satisfaction with the procedure

|  |  | The pain felt by the subject during the procedure and willingness to reuse depending on the satisfaction on the procedure are evaluated through a questionnaire on a 5-point scale. | |
| --- | --- | --- | --- |
|  |  |  |  |
| Safety | Assessed according to the definition of adverse events and assessment criteria outlined in Section 15. of the main text. | |  |
| Assessment Criteria |  |  |  |
|  |  |  |  |
| Observation Items | See 10.5. <Clinical Trial Schedule Table> | |  |
|  | • | Pain |  |
|  | • | Nausea |  |
|  | • | Vomiting |  |
|  | • | Abdominal tenderness |  |
|  | • | Edema |  |
|  | • | Abdominal cramping |  |
|  | • | 1-3 degree burns |  |
|  | • | Internal tissue thermal damage |  |
| Expected | • | Leg and hip pain |  |
|  | • | Vaginal bleeding more than before treatment |  |
| Adverse events | • | Sciatic nerve injury |  |
|  | • | Abdominal and pelvic organ damage (bladder, uterus, intestine, etc.) |  |
|  | • | Pain that does not respond to drugs |  |
|  | • | Urinary tract infection |  |
|  | • | Urination disorders |  |
|  | • | Fever due to infection | urticaria, burning sensation, blood pressure drop, heartbeat abnormality, dyspnea, kidney disease, acute renal failure, etc.) |
|  | • | Hypersensitivity reactions to the use of contrast agents (dizziness, nausea, vomiting, itching, |  |
|  |  |  | |
|  | • | Congestive changes in the abdominal muscles on MR examination |  |

(Limited to cases where there is a scar on the abdomen and the procedure is performed after applying a scar patch.)

ALPINION Medical Systems Co., Ltd. 7/45


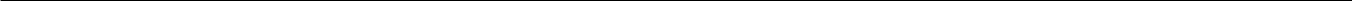

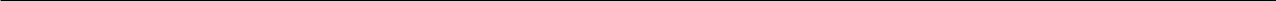


Protocol No. APM-03 Version 1.10/Date 20180103


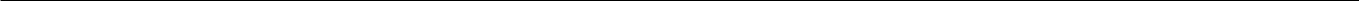


***<Clinical Trial Schedule Table>***

***
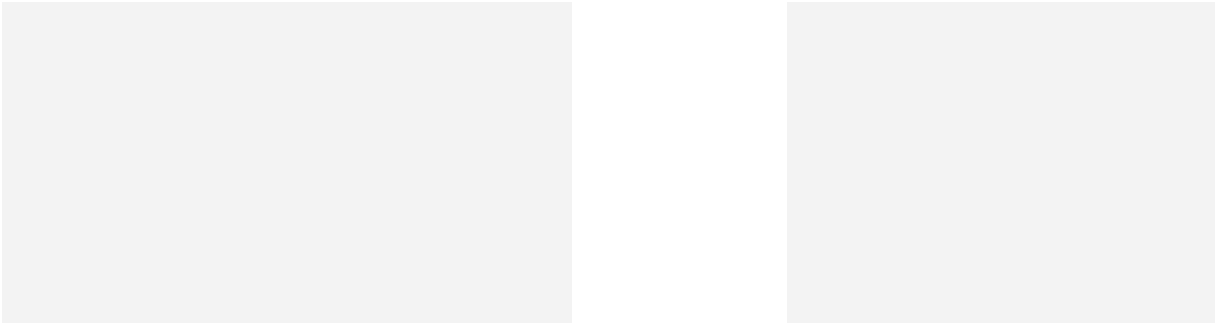
***

|  | Screening | Procedure day | Follow-up period | |
| --- | --- | --- | --- | --- |
| Visit day | Visit 1 | Visit 2* | Visit 3 | Visit 4 |
| Days passed | Day -30 to 0 | Day 0 | Month 1 | Month 3 |
| Visit window | - | - | ± 2 weeks | ± 2 weeks |
| Observation type | Clinic visit | Day surgery | Clinic visit | Clinic visit |
|  |  | Hospitalization |  |  |
|  |  |  |  |  |
| Obtain consent | √ |  |  |  |
| Inclusion/Exclusion criteria | √ |  |  |  |
| Demographic survey | √ |  |  |  |
| Vital signs | √ | √ |  |  |
| Physical examination | √ |  |  |  |
| Medical history survey | √ |  |  |  |
| ECG test | √ |  |  |  |
| Laboratory test | √ |  | √ |  |
| FSH | √ |  | √ |  |
| Pregnancy test | √ |  |  | √ |
| MRI | √ | √ | √ | √ |
| U/S | √ | √ | √ | √ |
| Dysmenorrhea Relief Score |  |  | √ | √ |
| Dysmenorrhea Score | √ |  | √ | √ |
| Menorrhagia Score |  |  |  |  |
| SF36-v2 | √ |  | √ | √ |
| UFS-QoL (including SSS) | √ |  | √ | √ |
| US-guided HIFU procedure |  | √ |  |  |
| Subject satisfaction with procedure |  | √ |  |  |
| Adverse event/Serious |  | √ | √ | √ |
| adverse event |  |  |  |  |
| Concomitant drugs | √ | √ | √ | √ |

ALPINION Medical Systems Co., Ltd. 8/45


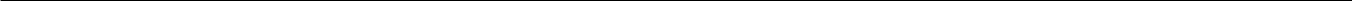

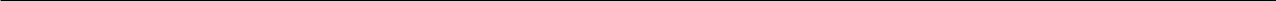


Protocol No. APM-03 Version 1.10/Date 20180103


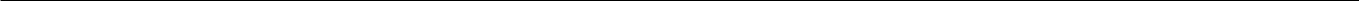


**< Table of Contents >**

Protocol Synopsis ··········································································································································· 3 Terminology ···································································································································································· 12

1. Name of Clinical Trial ··········································································································································· 13
2. Name and Address of Clinical Trial Site ····································································································· 13

3. Name and Title of Principal Investigator, Coordinators and Sub-investigators ······························································ 13

1. Name and Title of Investigational Medical Device Manager ········································································· 13
2. Name and Address of Clinical Trial Sponsor ············································································································· 14
3. Contract Research Organization (CRO) ······································································································································· 14
4. Background ································································································································································· 15

7.1. Background·························································································································································· 15

1. Purpose of Use of Investigational Medical Device············································································································· 16

8.1. Components of Investigational Medical Device·························································································································· 16

8.2. Mechanism of Action and Purpose of Use·························································································································· 16

1. Objectives of Clinical Trial ··········································································································································· 16
2. Expected Study Period ············································································································································· 17
3. Method and Procedures of Clinical Trial ······························································································································· 17 11.1. Study Design ················································································································································ 17

11.2. Target Disease and Indication ···························································································································· 17

11.3. Inclusion and Exclusion Criteria ························································································································ 17

11.3.1. Inclusion Criteria ···································································································································· 17

11.3.2. Exclusion Criteria···································································································································· 18

11.4. Calculation of Sample Size and Rationale···················································································································· 18

11.5. Observation Items, Clinical Laboratory Tests and Observation Method ···················································································· 20

11.5.1. Subject Number ······························································································································ 22

11.6. Clinical Trial Method········································································································································ 22

11.6.1. Instructions for Use for the Investigational Medical Device···························································································· 22

11.6.2. Clinical Trial Procedures ·························································································································· 24

11.6.3. Concomitant Therapy···································································································································· 27

11.6.4. Study Budget········································································································································ 27

1. Assessment of Study ················································································································································· 28 12.1. Efficacy Assessment·········································································································································· 28 12.1.1. Primary Efficacy Endpoint············································································································ 28

12.1.2. Secondary Efficacy Endpoint············································································································ 28

12.2. Assessment Criteria and Assessment Method ························································································································ 28

ALPINION Medical Systems Co., Ltd. 9/45


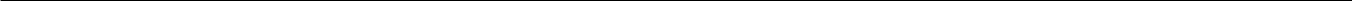

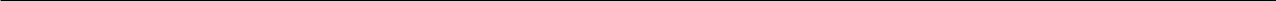


Protocol No. APM-03 Version 1.10/Date 20180103


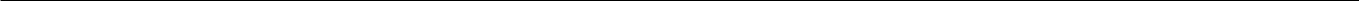


12.3. Safety Assessment············································································································································ 30

12.3.1. Safety Endpoints······················································································································ 30

12.3.2. Assessment Criteria and Assessment Method ············································································································ 30

1. Data Collection and Statistical Analysis························································································································· 30 13.1. General Considerations·································································································································· 30

13.2. Definition of Analysis Sets······························································································································ 30

13.2.1. Efficacy Analysis Set ·················································································································· 30

13.2.2. Safety Analysis Set·················································································································· 31

13.2.3. Handling Missing Data (missing values)········································································································ 31 13.3. Statistical Analysis Method········································································································································ 31 13.3.1. Analysis of General Items······································································································ 31

13.3.2. Primary Efficacy Analysis···················································································································· 31

13.3.3. Secondary Efficacy Analysis···················································································································· 31

13.3.4. Safety Analysis······························································································································ 32

1. Clinical Trial Discontinuation and Dropout Criteria····················································································································· 32 14.1. Discontinuation Criteria················································································································································ 33

14.2. Dropout Criteria················································································································································ 33

14.3. Handling Discontinuation·········································································································································· 33

14.4. Handling Dropouts·········································································································································· 33

1. Assessment Criteria, Assessment Method and Reporting of Safety Including Side Effects·························································· 33 15.1. Definition of Adverse Event·································································································································· 33

15.2. Definition of Serious Adverse Event/Medical Device Reactions················································································ 34

15.3. Assessment of Adverse Events·································································································································· 34

15.3.1. Severity Assessment································································································································ 34

15.3.2. Assessment of Causality with Investigational Medical Device·········································································· 34

15.4. Adverse Events Assessment Criteria ·························································································································· 35

15.5. Expected Adverse Events and Precautions······································································································ 35

15.5.1. Expected Adverse Events·················································································································· 35

15.5.2. Precautions for Use···················································································································· 35

15.6. Reporting Adverse Events······························································································································ 36

15.6.1. Adverse Event Training···························································································································· 36

15.6.2. Recording Expected Adverse Events···································································································· 36

15.6.3. Reporting Serious Adverse Events/Medical Device Reactions··········································································· 37

1. Measures for Subject Safety Protection················································································································· 38 16.1. Korean Good Clinical Practice (KGCP) for Medical Device and Declaration of Helsinki···································································· 38

16.2. Institutional Review Board (IRB) ·················································································································· 38

ALPINION Medical Systems Co., Ltd. 10/45


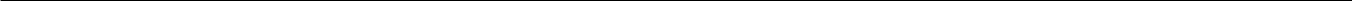

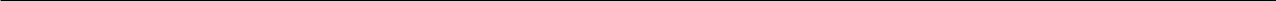


Protocol No. APM-03 Version 1.10/Date 20180103


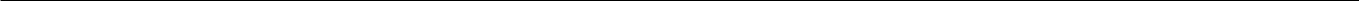


16.3. Subject Informed Consent Form······································································································································ 38

16.4. Indemnification Policy·················································································································· 39

16.5. Measures for Subject Safety Protection······································································································ 39

16.6. Care and Treatment Criteria of Subjects After Clinical Trial······················································································ 39

16.7. Clinical Trial Sites································································································································ 39

16.8. Investigators···················································································································································· 39

16.9. Sponsor···················································································································································· 40

1. Other Matters Necessary for Safe and Scientific Clinical Trial ········································· 40 17.1. ity················································································································································ 40 17.1.1. Data········································································································································ 40

17.1.2. Anonymity of Subjects······················································································································ 40

17.2. Protocol Compliance and Protocol Amendment························································································ 40

17.3. Clinical Trial Monitoring······························································································································ 41

17.4. Recording and Use of Study Results·················································································································· 42

17.4.1. Case Report Forms and Source Documents·········································································································· 42

17.4.2. Retention of Clinical Study Data·············································································································· 42

17.4.3. Use of Study Results······················································································································ 43

17.5. Agreement Between Sponsor and Head of Clinical Trial Site ·························································································· 43

17.6. CV of Principal Investigator ······················································································································ 43

17.7. Use and Management of Investigational Medical Device·························································································· 43

17.8. Supply and Handling of Investigational Medical Device······························································································ 43

1. References ······················································································································································· 44

ALPINION Medical Systems Co., Ltd. 11/45


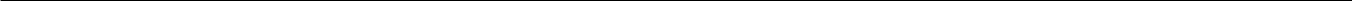

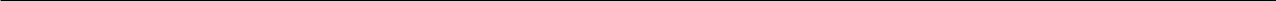


Protocol No. APM-03 Version 1.10/Date 20180103


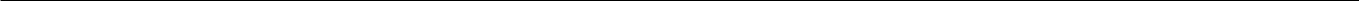


***< Terminology >***

- Ablation
- Acoustic Power
- ADE (Adverse Device Effect)
- AE (Adverse Event)
- CRA (Clinical Research Associate)
- DVT (Deep Vein Thrombosis)
- FAS (Full Analysis Set)
- FSH (Follicle-Stimulating Hormone)
- HIFU (High Intensity Focused Ultrasound)

⑩ IRB (Institutional Review Board)

- KGCP (Korea Good Clinical Practice)
- MRgHIFU (Magnetic Resonance Imaging-Guided High Intensity Focused Ultrasound)
- MRI (Magnetic Resonance Imaging)

⑭ PP (Per-Protocol)

- SAE (Serious Adverse Event)
- T1 weighted image
- T2 weighted image
- USgHIFU (Ultrasound-Guided High Intensity Focused Ultrasound)
- NRS (Numeric Rating Scale)

ALPINION Medical Systems Co., Ltd. 12/45


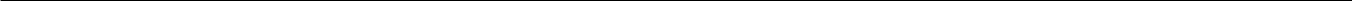

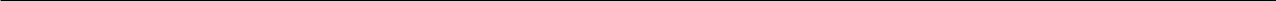


Protocol No. APM-03 Version 1.10/Date 20180103


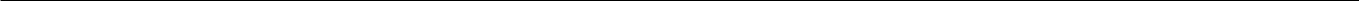


1. **Name of Clinical Trial**

A prospective, multicenter, single-arm confirmatory clinical trial to evaluate the safety and efficacy of ‘ALPIUS 900’ (US-guided HIFU System, ultrasound-guided, high-intensity focused ultrasound surgery device) in patients with adenomyosis

1. **Name and Address of Clinical Trial Site**
   - Seoul National University Hospital

101 Daehang-ro (Yeongeon-dong 28), Jongno-gu, Seoul (03080), Telephone: 02-2072-2114

- - Konyang University Hospital

158 Gwanjeodong-ro, Seo-gu, Daejeon (35365), Telephone: 1577-3330

1. **Name and Title of Principal Investigator, Coordinators, and Sub-investigators**

- Seoul National University Hospital

Principal Investigator:

Sub-investigator:

Sub-investigator:

Sub-investigator:

Sub-investigator:

Sub-investigator:

Clinical Research Coordinator:

Study Nurse:

Medical Device Manager:

| Prof. Jae Yeong Lee | Department of Radiology |  |
| --- | --- | --- |
| Prof. Maria Lee | Department of Obstetrics and Gynecology |  |
| Prof. Myeong Jae Jeon | Department of Obstetrics and Gynecology |  |
| Prof. Ki Dong Kim | Department of Obstetrics and Gynecology |  |
| Prof. Hoon Kim | Department of Obstetrics and Gynecology |  |
| Prof. Chang Soon Lee | Department of Anesthesiology and Pain Medicine |  |
| Nurse Hye Shin Yoon | Department of Radiology |  |
| Nurse Soo Yeon Kang | Department of Radiology |  |
| Radiologist Dong Hyeok Park | Department of Radiology |  |

- Konyang University Hospital

Principal Investigator:

Sub-investigator:

Sub-investigator:

Sub-investigator:

Study Nurse:

| Prof. Cheol Joong Kim | Department of Obstetrics and Gynecology |  |
| --- | --- | --- |
| Prof. Tae Hyeon Kim | Department of Obstetrics and Gynecology |  |
| Prof. Seong Ki Lee | Department of Obstetrics and Gynecology |  |
| Prof. Seong Eun Heo | Department of Obstetrics and Gynecology |  |
| Nurse Da Mi Choi | Department of Obstetrics and Gynecology |  |

ALPINION Medical Systems Co., Ltd. 13/45


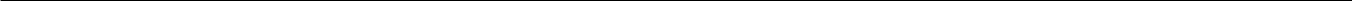

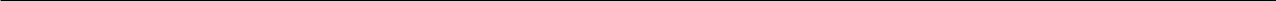


Protocol No. APM-03 Version 1.10/Date 20180103


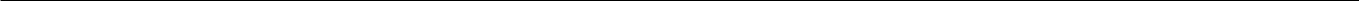


1. **Name and Title of Investigational Medical Device Manager**

■ Seoul National University Hospital /Dong Hyeok Park, Radiology technician, Department of Radiology

■ Konyang University Hospital / Da Mi Choi, Clinical Research Coordinator

1. **Name and Address of Clinical Trial Sponsor**

■ ALPINION Medical Systems Co., Ltd., CEO: Yeong Choon Choi

1st, 6th FL., Verdi Tower, Guro Digital-ro 26 gil 72, Guro-gu, Seoul (02-3282-0903)

1. **Contract Research Organization**

Synex Co., Ltd., CEO Yeong Kim

10th FL., ASEA TOWER, 430 Nonhyeon-ro, Gangnam-gu, Seoul (06223) Monitor: Ga Yeong Park CRA (02-6202-3376)

ALPINION Medical Systems Co., Ltd. 14/45


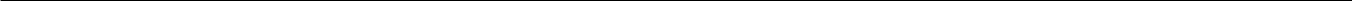

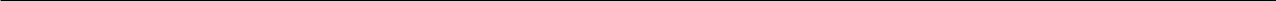


Protocol No. APM-03 Version 1.10/Date 20180103


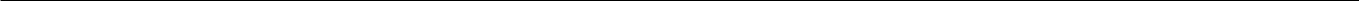


1. **Background**

**7.1. Background**

Adeomyosis is a benign disease that occurs in the uterus and is one of the common gynecological diseases. It has a wide range of prevalence of 1 to 70% depending on race, and is usually reported in 20 to 30% of women of reproductive age.^1^ Although the exact cause of adenomyosis is not known, it appears to be caused by the penetration of endometrial tissue into the myometrium,^2^ which causes the size of the uterus to resemble that of the enlarged uterus during pregnancy. Typical symptoms of adenomyosis include menorrhagia and severe dysmenorrhea, and other symptoms such as pelvic pain and frequent urination may occur.^1^ These symptoms are very severe in patients with adenomyosis, so the goal of treatment for adenomyosis is to control symptoms including relieving dysmenorrhea.

Adenomyosis can be treated in several ways depending on the severity of the symptoms, but the most reliable method is known as hysterectomy. However, hysterectomy is not suitable for women who want to become pregnant in the future, and there are disadvantages such as bleeding, blood transfusion, organ adhesion, and the risk of complications due to general anesthesia. In addition, it can be treated through myomectomy, which can preserve the uterus, but there are also disadvantages in that there may be complications from surgery and risk of uterine rupture during pregnancy or childbirth. In addition to these, uterine artery embolization and hormone therapy can be applied in consideration of the patient's lifestyle, cost, and degree of invasion. Uterine artery embolization is a method of killing the adenomyoma cells by inducing embolization in the uterine artery that supplies blood to the uterus to block the blood flow. Although it does not require general anesthesia and has the advantage of rare complications due to the low possibility of bleeding, there is a risk of side effects such as menopause or requiring hysterectomy due to necrosis and infection. In addition, hormone therapy is a treatment using gonadotropin, which reduces the size of adenomyoma by suppressing the production of estrogen associated with the size of adenomyoma; although a non-invasive method, it is used for the purpose of alleviating symptoms for a short period of time or reducing the size of myoma before surgery, and cannot be the ultimate treatment method.

For this reason, many studies are being conducted on the high-intensity focused ultrasound (HIFU) system, which is one of the non-invasive procedures that can compensate for the shortcomings of invasive procedures and drug therapy. The principle of HIFU technology is to focus the irradiated high-intensity ultrasound energy in a limited local area through a transducer and convert it into thermal energy in the focal area, and the temperature in the focal area rapidly rises to 55℃ or higher, resulting in thermal cauterization of the tissue. This technology that can treat the affected area without damage to the normal tissue since there is almost no temperature rise in the area outside the focus of HIFU.^11^

The options for the treatment site for focusing high-intensity ultrasound includes MR guided HIFU using MRI images and US guided HIFU using ultrasound images.

MRgHIFU is a system that connects the high-intensity ultrasound focusing device to the MRI system, selects the location of the tissue to be cauterized through the MR image, treats the selected tissue using HIFU, and observes the treatment process and treatment effect through the MRI image. Previous studies have confirmed good therapeutic effects.^5-7^ However, it also has the following limitations: 1) since the high-intensity focused ultrasound device is connected to the MR system, MRI for imaging cannot be performed while the treatment is in progress, 2) the need for a separate MRI room, 3) high cost of equipment and treatment, 4) treatment cannot be performed while patient is moving as real-time imaging treatment is not possible because it uses MR images, 5) treatment is time-consuming, and 6) the market is limited as the demand is mostly from tertiary hospitals.

ALPINION Medical Systems Co., Ltd. 15/45


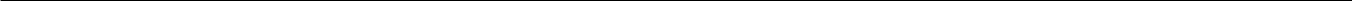

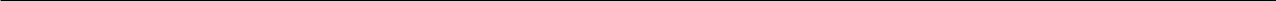


Protocol No. APM-03 Version 1.10/Date 20180103


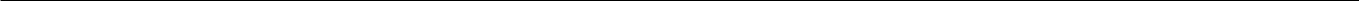


In contrast, USgHIFU is a system that selects the location of the tissue to be cauterized using the image acquired through the ultrasound imaging device, treats it using HIFU while observing the treatment process and treatment effect on the ultrasound image. Its advantages include: 1) it is cheaper than MRgHIFU and other treatment equipment, 2) cost of treatment is also relatively cheap, 3) treatment can be provided with real-time images, 4) shorter treatment duration, 5) a separate MRI room is not required, and 6) other patients can use the MRI during treatment.

The HIFU procedure has been proven to be effective in relieving the symptoms of uterine fibroids through several studies, and several studies have recently been conducted to show the effect of treating adenomyosis. An overseas manufacturer conducted an initial clinical trial with its MRgHIFU product to confirm the therapeutic efficacy of HIFU in Japan in 2008, published results of the study that HIFU treatment was helpful in alleviating the symptoms of adenomyosis patients^4^, and obtained CE marking for adenomyosis in 2010. In addition, several studies^1-3^ confirmed that HIFU was safe and effective as an alternative treatment for adenomyosis.

In the light of such background, ALPINION Medical Systems Co., Ltd. developed the US-guided HIFU ‘ALPIUS 900’, a product that retains the performance of high-intensity focused ultrasound surgery devices such as MR-guided HIFU while having small volume and excellent mobility, and has obtained domestic marketing approval by demonstrating the efficacy and safety for uterine fibroids (Manufacturing Approval No. 14-3227).This clinical trial was designed to demonstrate the efficacy and safety of the treatment for adenomyosis by expanding the indications approved for 'ALPIUS 900' (for MFDS product approval), which currently includes uterine fibroids.

1. **Purpose of Use of Investigational Medical Device**

■ Product Name: ALPIUS 900 (Manufacturing Approval No. 14-3227)

■ Product Category: High intensity focused Ultrasonic surgical unit (Grade 3, A35100.02), a device for the treatment of cancer using high-intensity focused ultrasound

■ Previously approved items: Non-invasive treatment of uterine fibroids of size between 3 cm and 12 cm using high-intensity focused ultrasound according to ultrasound image guidance

**8.1. Components of Investigational Medical Device**

<See Attachments> ALPIUS 900 User Manual

**8.2. Mechanism of Action and Purpose of Use**

ALPIUS 900 is used to cauterize adenomyosis by irradiating high-intensity focused ultrasound under ultrasound image guidance.

1. **Objective of Clinical Trial**

This clinical trial is conducted to confirm the efficacy and safety of the non-invasive adenomyosis ablation for “symptomatic adenomyosis” using 'ALPIUS 900 (US-guided HIFU System)', a high-intensity focused ultrasound surgical device by ALPINION Medical Systems Co., Ltd. approved for uterine fibroids.

ALPINION Medical Systems Co., Ltd. 16/45


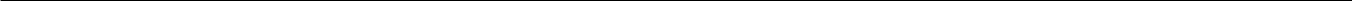

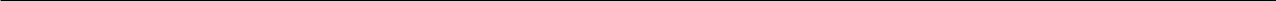


Protocol No. APM-03 Version 1.10/Date 20180103


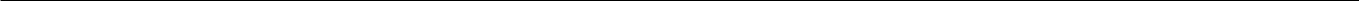


(For MFDS approval)

1. **Expected Study Period**

The study is expected to take approximately 28 months, including approximately 25 months of subject enrollment after obtaining clinical trial protocol approval from the Ministry of Food and Drug Safety (MFDS) and 3 months of follow-up. Even after the clinical trial is completed, it is expected to take approximately 3 months in addition for data processing, statistical analysis, clinical study report preparation and IRB approval.

1. **Method and Procedures of Clinical Trial**

**11.1. Study Design**

This clinical trial was designed to evaluate the safety and efficacy of 'ALPIUS 900', a high-intensity focused ultrasound surgical device guided by ultrasound images, for symptomatic patients with adenomyosis. As a prospective, multicenter, single-arm clinical trial, it will be conducted with a total of 80 patients at 2 domestic institutions.

**11.2. Target Disease and Indication**

Patients requiring cauterization of adenomyosis

**11.3. Inclusion and Exclusion Criteria**

**11.3.1. Inclusion Criteria**

Subjects can be enrolled in this clinical trial only if they meet all of the following inclusion criteria.

1. Adult female of at least 20 years of age
2. Pre-menopausal or peri-menopausal (FSH<40mIU/ml)
3. Adenomyosis is clinically diagnosed through MR or US imaging
4. Pain score on the Dysmenorrhea Score is at least 4 points
   - Dysmenorrhea Score

1 = Not at all (no symptoms)

2 = A little bit (slight symptoms)

3 = Somewhat (few symptoms)

4 = A great deal (considerable symptoms)

5 = A very great deal (a lot of symptoms)

1. Have not received any other treatment for adenomyosis within 3 months before HIFU procedure

*☞ In the case of hormone therapy, even within 3 months, subjects can be enrolled if five times the half-life of the drug has elapsed since the last administration.*

ALPINION Medical Systems Co., Ltd. 17/45


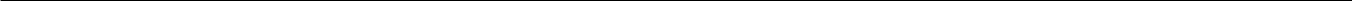

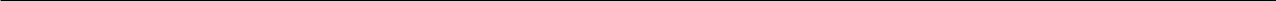


Protocol No. APM-03 Version 1.10/Date 20180103


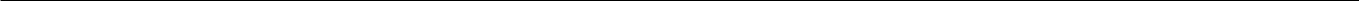


1. Those who voluntarily consent to the clinical trial and are willing to comply with the protocol
2. Those who agree to use a medically accepted method of contraception for the duration of the clinical trial
   - *Medically accepted method of contraception: Physical contraceptive devices other than contraceptives through hormonal control such as condoms or installation of intrauterine contraceptive devices*

**11.3.2. Exclusion Criteria**

Subjects cannot be enrolled in this clinical trial if they meet any of the following exclusion criteria.

1. Presence of other pelvic diseases such as other malignant tumors, endometriosis, ovarian tumors, or acute pelvic disease
2. Sarcoma is clinically suspected
3. Diffuse adenomyosis, in which adenomyosis has spread throughout the uterus, is clinically suspected
4. Positive result in pregnancy test or plans to get pregnant
5. Presence of a serious systemic disease
6. If hematocrit is less than 25%
7. There is an extensive abdominal scar in the area where the ultrasound light passes
   - *However, if the investigator decides that HIFU treatment can be performed by applying a scar patch even if there is an extensive abdominal scar in the area where the ultrasound light passes, this exclusion criterion will not exclude the subject from the clinical trial.*
8. There is a scar or surgical clip in the passageway through which the high-intensity focused ultrasound passes
9. If the subject cannot lie down in a comfortable position
10. Contraindications to MRI (including those with claustrophobia)
11. Contraindications to MRI contrast agents
12. Contraindications to ultrasound contrast agents
13. When GFR (Glomerular filtration rate) is 30ml/min or less
14. In case communication is difficult
15. Patients who have participated in other clinical trials within the last 1 month
16. Other subjects deemed ineligible to participate in this clinical trial according to the judgment of the investigator

- Record specific reasons in the case report form

**11.4. Calculation of Sample Size and Rationale**

According to the results of a study by Zhou et al. (2011)^3^ evaluating the efficacy of US-guided HIFU for the treatment of adenomyosis, 62 people out of a total of 69 subjects experienced relief of dysmenorrhea after treatment, showing a 90% improvement success rate. Based on these results, the reference success rate was assumed as 90% in this clinical trial to calculate the number of subjects required to satisfy the hypothesis that the success rate of the ‘ALPIUS device will be non-inferior when compared to the reference success rate’.

ALPINION Medical Systems Co., Ltd. 18/45


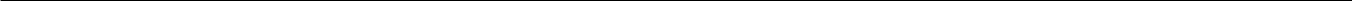

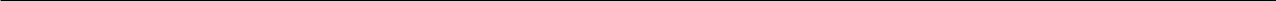


Protocol No. APM-03 Version 1.10/Date 20180103


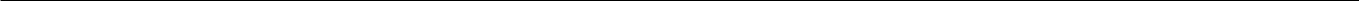


In addition, in the results of a recent study evaluating the efficacy of the US-guided HIFU procedure for adenomyosis, Zhang et al. (2014)^5^ evaluated the degree of relief of menorrhagia and dysmenorrhea and showed a success rate of 79.5% to 87.9% while Wang et al. (2009)^12^ suggested an improvement success rate of 66.7%; thus, we assumed that 77.7%, which is greater than the minimum value of 66.7%, would be the ‘minimum success rate that satisfies non-inferiority’ in this study (i.e. non-inferiority threshold = 77.7%-90% = -12.3%).

The statistical hypothesis of this study is as follows.

H_0_: P_A_ - P_0_ ≤ - δ vs H_1_: P_A_ - P_0 >_ - δ

- - P_A_: Expected success rate of the investigational medical device
  - P_0_: Reference success rate
  - δ (>0): Non-inferiority threshold

To calculate the number of samples for hypothesis testing, the number of subjects satisfying the non-inferiority hypothesis was calculated as follows through PASS13 (NCSS statistics software, Kaysville, UT) based on the following assumptions: significance level of 0.025, power of 80%, reference success rate and expected success rate of this device are set at 90%, non-inferiority limit of 12.3%, the difference between the actual success rate and the reference success rate as D=0.


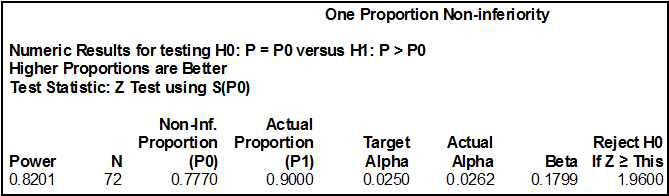


As a result, a total of 72 subjects were calculated, and 80 subjects were enrolled in consideration of the dropout rate of 10%.

<Reference for Calculation of Number of Subjects>

1. X Zhang et al. Effective ablation therapy of adenomyosis with ultrasound-guided high-intensity focused ultrasound. International Journal of Gynecology and Obstetrics. 2014;124:207-211.
2. M Zhou et al. Ultrasound-guided high-intensity focused ultrasound ablation for adenomyosis: the clinical experience of a single center. Fertility and Sterility. 2011;95(3):900-905.
3. W Wang et al. Safety and efficacy of high intensity focused ultrasound ablation therapy for adenomyosis. Academic Radiology. 2009;16(11):1416-1423.

ALPINION Medical Systems Co., Ltd. 19/45


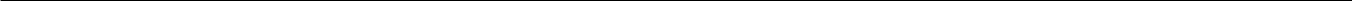

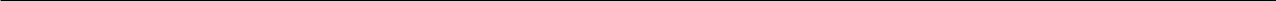


Protocol No. APM-03 Version 1.10/Date 20180103


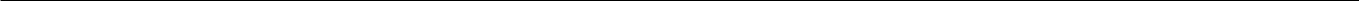


**11.5. Observation Items, Clinical Laboratory Tests and Observation Method**

<Clinical Trial Schedule Table>


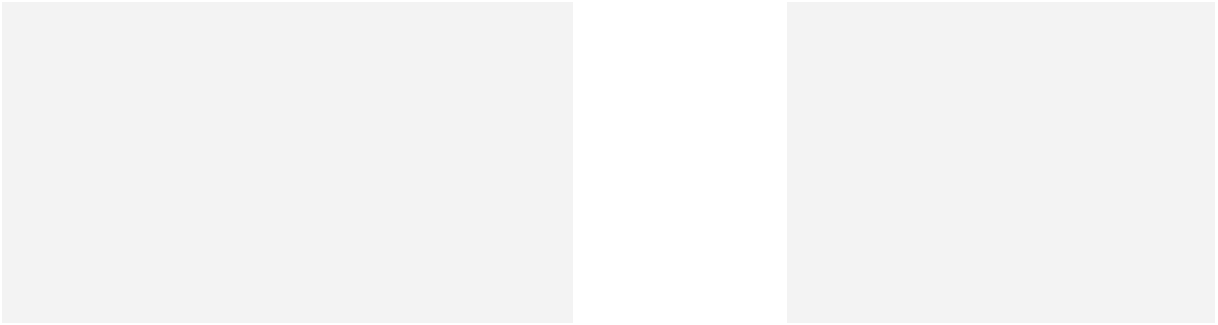


|  | Screening | Procedure day | Follow-up period | |
| --- | --- | --- | --- | --- |
| Visit day | Visit 1 | Visit 2* | Visit 3 | Visit 4 |
| Days passed | Day -30 to 0 | Day 0 | Month 1 | Month 3 |
| Visit window | - | - | ± 2 weeks | ± 2 weeks |
| Observation type | Clinic visit | Day surgery | Clinic visit | Clinic visit |
|  |  | Hospitalization |  |  |
|  |  |  |  |  |
| Obtain consent | √ |  |  |  |
| Inclusion/Exclusion criteria | √ |  |  |  |
| Demographic survey^l^ | √ |  |  |  |
| Vital signs^2^ | √ | √ |  |  |
| Physical examination^3^ | √ |  |  |  |
| Medical history survey^4^ | √ |  |  |  |
| ECG test^5^ | √ |  |  |  |
| Laboratory test^6^ | √ |  | √ |  |
| FSH | √ |  | √ |  |
| Pregnancy test^7^ | √ |  |  | √ |
| MRI^8^ | √ | √ | √ | √ |
| U/S^9^ | √ | √ | √ | √ |
| Dysmenorrhea Relief Score^10^ |  |  | √ | √ |
| Dysmenorrhea Score ^11^ | √ |  | √ | √ |
| Menorrhagia Score ^12^ | √ |  | √ | √ |
| SF36-v2^13^ | √ |  | √ | √ |
| UFS-QoL (including SSS)^14^ | √ |  | √ | √ |
| US-guided HIFU procedure |  | √ |  |  |
| Subject satisfaction with procedure^15^ |  | √ |  |  |
| Adverse event/Serious |  | √ | √ | √ |
| adverse event^16^ |  |  |  |  |
| Concomitant drugs^17^ | √ | √ | √ | √ |

1. Demographic survey

Basic information of subjects including date of birth, sex, height and body weight

1. Vital signs

Body temperature, blood pressure (systolic/diastolic), pulse, respiration rate

ALPINION Medical Systems Co., Ltd. 20/45


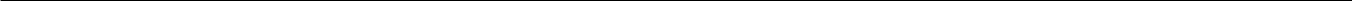

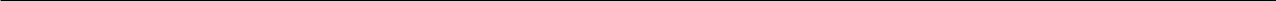


Protocol No. APM-03 Version 1.10/Date 20180103


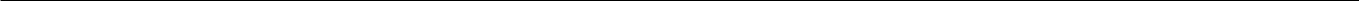


1. Physical examination

Appearance, skin, head/neck, chest/lung, heart, abdomen, urinary/genital, extremities, musculoskeletal system, nervous system, lymph nodes, other body organs

1. Medical history survey

Survey of medical history during the past 1 year

1. ECG test

Electrocardiography is performed as a screening test for the procedure. If an abnormality is found on the electrocardiogram, the subject cannot be enrolled in this clinical trial.

1. Laboratory test

Laboratory tests are performed on all subjects to evaluate their overall health, and the test items are as follows.

■ Hematology: Hemoglobin, Hematocrit, RBC count, WBC count with differential, platelet

count

■ Blood Chemistry: Alkaline phosphatase, BUN, Creatinine, SGPT(ALT), SGOT(AST), Albumin, Total protein, Total bilirubin, Uric acid, Glucose, Cholesterol, LDH, Na, K, Cl, Ca

1. Pregnancy test

For women of childbearing potential, except for subjects whose sterilization and menopause were confirmed through the questionnaire, pregnancy is confirmed using the urine HCG (however, at the Screening visit, the pregnancy test must be done within 3 days before the procedure).

1. MRI

At the Screening visit, T1- and T2-weighted images before and after contrast enhancement are taken on three orthogonal planes to check the size of the uterus, the location of the adenomyoma, the response to the contrast agent, and the beam passage. Immediately after the procedure and during the follow-up period, contrast-enhanced fat-saturated T1-weighted and T2-weighted images are acquired to check the treated volume against to the plan and to confirm the change in the size of the uterus (4 times in total).


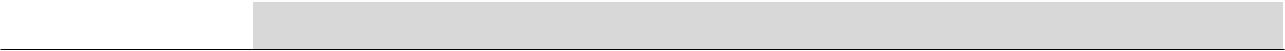


| Model Name | Product Name | Approval No. | Importer |
| --- | --- | --- | --- |
| Ingenia 3.0T | Superconducting magnet for whole body | Import Approval No. 11-1257 | Philips Korea Ltd. |
|  | Magnetic resonance computerized tomography device |  |  |
|  |  |  |  |

1. U/S

At the screening visit, an ultrasound examination is performed to confirm the size of the uterus and the location of the adenomyoma, and during treatment, the actual treatment is monitored through the image of the ultrasound transducer built into the ALPIUS 900.Immediately after the procedure, the condition of the uterus and adenomyoma after the procedure is checked through ultrasound examination. During the follow-up period, the size of the uterus is checked by ultrasound images at Months 1 and 3. (4 times in total) Contrast agents may be used for ultrasound examination, and detailed examination methods should follow the judgment of the investigator and the standard procedures of the institution.


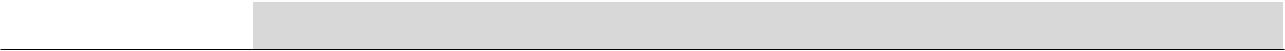


| Product Name | Product Name | Approval No. | Importer |
| --- | --- | --- | --- |
| LOGIQ E9 | Universal ultrasound imaging device | Import Approval No. 09-180 | GE Healthcare Korea Co., Ltd. |

ALPINION Medical Systems Co., Ltd. 21/45


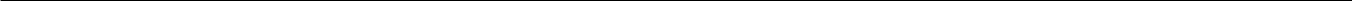

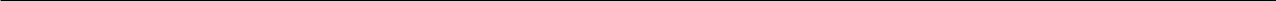


Protocol No. APM-03 Version 1.10/Date 20180103


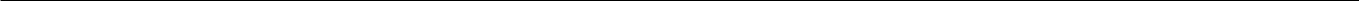


1. Dysmenorrhea Relief Score

Each subject directly evaluates the degree of dysmenorrhea relief on a 5-point scale using a questionnaire at 1 and 3 months after the procedure.

1. Dysmenorrhea Score

The symptoms of dysmenorrhea are assessed for each subject on a 5-point scale using a questionnaire before the procedure as well as 1 and 3 months after the procedure.

1. Menorrhagia Score

The symptoms of menorrhagia are assessed for each subject on a 5-point scale using a questionnaire before the procedure as well as 1 and 3 months after the procedure.

1. SF36-v2

This is a questionnaire completed directly by the subject to evaluate the overall quality of life, and consists of 36 questions in 8 domains including physical functioning, role limitation-physical, bodily pain, general health, vitality, social functioning, role limitation-emotional, mental health, and other changes in health condition, etc.

1. UFS-QoL (including SSS)

As a questionnaire completed directly by the subject to evaluate the quality of life related to the symptoms of uterine fibroids, it consists of an 8-item Symptom Severity Score and a 29-item Quality of Life questionnaire.

1. Subject satisfaction with procedure

The pain felt by the subject during the procedure and willingness to reuse depending on the satisfaction on the procedure is evaluated through a questionnaire on a 5-point scale.

1. Adverse event/Serious adverse event

Assess according to the criteria defined in Section 16 of the Protocol and record on the Adverse Event/Serious Adverse Event record form in the CRF.

1. Concomitant drugs

Drugs that may affect the results of the clinical study are prohibited, and all drugs already being taken due to underlying diseases are recorded at the time of Screening. Only the drugs added thereafter are recorded in the CRF.

- After the procedure on Visit 2, the subject can be discharged from the hospital on the day of the procedure or hospitalized for about one day according to the judgment of the investigator and the standard procedure of the hospital.

**11.5.1. Subject Number**

A management log will be created for all subjects who have signed the subject consent form and are enrolled for study participation. This management log is used to assign sequential subject numbers to subjects enrolled in the clinical trial, and a ‘Screening number’ and an ‘enrollment number’ will be assigned to subject numbers.

ALPINION Medical Systems Co., Ltd. 22/45


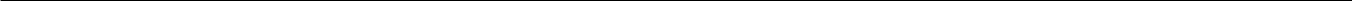

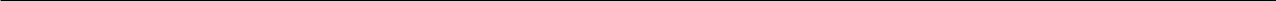


Protocol No. APM-03 Version 1.10/Date 20180103


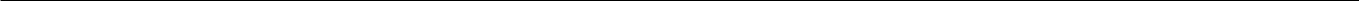


The Screening number is assigned as a subject identification number according to the method below, by including one-digit institution number, ‘S’ for screening and a two-digit number assigned in the order of subject such as 01, 02, 03, and so on.

- One-digit clinical trial site number: Seoul National University Hospital –1, Konyang University Hospital -2

- Screening: S

- Two-digit number in the order of enrollment:

Example) The subject identification number for the first screened subject at the Seoul National University Hospital: 1 S – 0 1

The enrollment number is assigned as a subject identification number according to the method below, by including one-digit institution number and a two-digit number assigned in the order of subject such as 01, 02, 03, and so on.

- One-digit clinical trial site number: Seoul National University Hospital –1, Konyang University Hospital -2 - Two-digit number in the order of enrollment

Example) The subject identification number for the first enrolled subject at the Seoul National University Hospital: 1 – 0 1

**11.6. Clinical Trial Method**

**11.6.1. Instructions for Use for the Investigational Medical Device**

The procedure for using the investigational medical device is as follows, and for details of each subparagraph, follow the manufacturer’s instruction manual (attached).

Preparations Before Use

§ Check power and ON/OFF

§ Check image transducer connection and disconnection

§ Check whether image transducer is enabled or disabled

Instructions for Use and Operation Method

① Prepare device

② Prepare subject

1. Pre-procedure process
2. Outpatient treatment
   - Subjects who have received CT, MRI, or ultrasound images from the hospital where adenomyosis was first diagnosed, diagnostic images from the hospital currently providing treatment, or other treatment history or findings, etc. are treated. When a subject is identified as eligible for treatment, an appointment for the procedure is scheduled.
3. Preparation of the subject before the procedure (according to the surgeon's prescription)
   - Fasting the day before procedure
   - Keep clean skin
   - Hair removal depending on skin condition (mechanical and chemical hair removal)
   - Administer anesthetic or sedation according to the prescription of the procedure

ALPINION Medical Systems Co., Ltd. 23/45


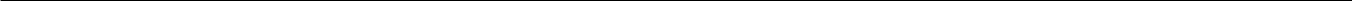

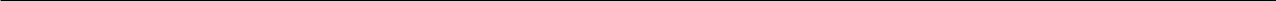


Protocol No. APM-03 Version 1.10/Date 20180103


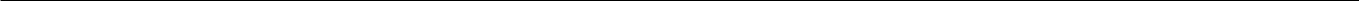


● Position : Supine or lateral

● Measurement of vital signs (blood pressure, pulse, respiration rate)

2. HIFU Procedure

1. After checking subject preparation for the procedure, the treatment area is scanned. The surgeon cauterizes the lesion tissue according to the pre-established procedure plan. The procedure usually causes tissue necrosis with a single procedure.
2. The surgeon checks the necrosis of the lesion in real time during the procedure through ultrasound and, if necessary, fixes the subject in place.

1. Post-procedure process
   1. Subjects who have completed treatment may return home on the day of treatment to engage in social activities or be hospitalized for about a day and return home the next day according to the opinion of the medical staff.
   2. Outpatient visit and follow-up tests: MRI is performed at 1 month and 3 months after the procedure to track the effect of the procedure and to measure the volume of the necrotic lesion tissue.
2. Precautions for Use

<See Attachments> ALPIUS 900 User Manual

**11.6.2. Clinical Trial Procedures**

1. **Screening <Visit 1>**

Patients diagnosed with adenomyosis through MR or US imaging are referred to this clinical trial by a gynecologist or interventional radiology department. They will be enrolled in this clinical trial if they satisfy all of the inclusion/exclusion criteria and sign the written consent form voluntarily after being given sufficient explanation on this study.

At the Screening visit, in addition to the inclusion/exclusion criteria, demographic survey, vital signs, physical examination, past medical history, blood tests, pregnancy tests, ECGs, MRIs, ultrasound tests, and questionnaires are used to evaluate the degree of menstrual pain and menorrhagia. The ability to communicate with the investigator and willingness to comply with the clinical trial will also be assessed during the Screening period.

Pre-procedure MRI

An intravenous catheter may be inserted prior to the procedure in order to obtain MRI images before and after using the contrast medium as well as the T1- and T2-weighted images for 3 orthogonal planes, and to administer drugs such as MR contrast agents and weak stabilizers.

Pre-procedure MRI is the final step in determining inclusion/exclusion. For post-treatment MR imaging, a contrast medium commonly used for gynecological imaging in hospitals will be used according to the product manual. (Follow the standard procedure of the hospital)

ALPINION Medical Systems Co., Ltd. 24/45


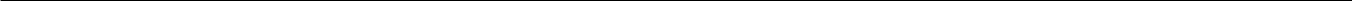

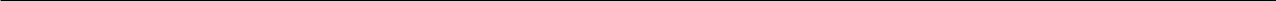


Protocol No. APM-03 Version 1.10/Date 20180103


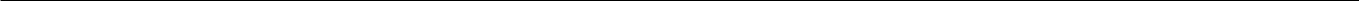


1. **Procedure day <Visit 2>**

- HIFU Treatment Protocol

The US-guided HIFU procedure is expected to take 2 to 4 hours, including recovery time (1 to 2 hours) after the procedure is completed. The duration for which the subject remains immobile should be limited to about 3 hours to reduce the risk of deep vein thrombosis (DVT).

- Preparation

Subjects will have hair removal (using hair removal cream) on the pelvic area within 3 days before the procedure, and do not use any cream after shaving. Although general anesthesia will not be performed, to reduce side effects from gas or food in the gastrointestinal tract, the subject should fast from midnight the day before the procedure (MN NPO). On the day of the procedure, ensure that there is an adequate amount of urine in the bladder; if necessary, fill in more urine or urinate to maintain the bladder volume suitable for treatment. Even if the pregnancy test was performed at Screening, if it does not fall within the period within 3 days before the procedure, the test should be repeated, and vital signs such as blood pressure, pulse rate, and respiration rate will also be recorded. The subject's body temperature will also be recorded for use as basal temperature information for treatment.

■ Identification and treatment of adenomyosis

The volume of the adenomyoma to be treated will be determined by the investigator and will be based on the present concomitant symptoms (dysmenorrhea, tenderness, excessive bleeding). Based on the contrast-enhanced MR images of the uterus before treatment, only adenomyomas with enhancement of the contrast agent will be considered for treatment in this study. Adenomyomas that are not enhanced by the contrast medium should not be treated.

*The following areas should not be treated.*

1. Ultrasound should not be irradiated within 1.5 cm of the uterine serosa.
2. Ultrasound should not be irradiated within 4 cm of the spine or other bone tissue.
3. Ultrasound can be irradiated to the endometrium surface.

■ Positioning the Subject and Treatment Plan

1. Ensure that the subject is in supine position on the treatment table.
2. Apply the ultrasonic gel sufficiently to the subject's skin so that the therapeutic ultrasonic transducer is in close contact.
3. After the ultrasonic transducer for treatment is lowered and brought into close contact with the subject's lower abdomen, scan image information is obtained from the area to be treated.
4. The acquired image can be expressed in XZ, YZ, XY plane images to search for a desired scan and treatment area.
5. The treatment area is marked by activating the program that draws the area to be treated (Target Draw).

ALPINION Medical Systems Co., Ltd. 25/45


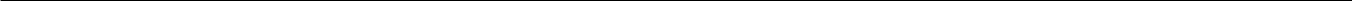

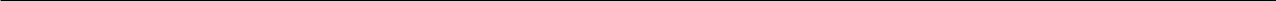


Protocol No. APM-03 Version 1.10/Date 20180103


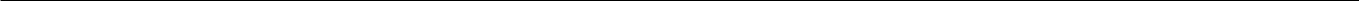


1. Based on the selected area, the following HIFU irradiation method can be configured in detail.
   - Focal Point Grid: The spacing (X, Y) between points and the Cartesian (lattice) and Cylinder (cylinder) type focal point placement can be configured.

- - Acoustic Power : Acoustic Power (Intensity)
  - PRF (Pulse Repetition Frequency) : Number of irradiation per second
  - Duty : The duration of the irradiation is configured in units of percentage in the time interval between irradiation.
  - Exposure Time : Duration of irradiation on one focal point
  - Point to Point : Travel time between points
  - Cooling Time : Travel time between slice images
  - Generate Points: A focal point is automatically created in a pattern of Cartesian (lattice) and Cylindrical (cylindrical) in the desired area.
  - Add Point: You can manually create focal points that do not have a uniform pattern in a desired area one by one.

1. Based on the treatment plan of the subject set in the preceding step, simulation is performed before actual treatment to check the validity of the treatment plan.
2. Pre-targeting is performed to test the accuracy of the HIFU irradiation before treating the subject. In pre-targeting, the user arbitrarily sets the location to be irradiated, and actually irradiates the HIFU weakly to check its accuracy through the irradiation location information.

- Treatment Procedure

The operator performs HIFU treatment according to a pre-established treatment plan, and observes the necrosis of the lesion and the progress of treatment through ultrasound images and software. If the position of the uterus is shifted due to urine filling in the bladder, adjust the treatment position and start again. Upon completion of the planned treatment of the entire site, a record of the entire treatment is kept in the subject record.

- After Treatment

When the treatment is completed according to the initially set treatment range, the surgeon acquires an image of the treatment area through ultrasound and MRI to check the planned volume and the range of the treatment volume, and observes for any adverse reactions including changes in the skin. Subjects may be discharged from the hospital on the day of treatment or the next day if there is no abnormality after observing the passage of 1 to 2 hours in the recovery room, and fill out a questionnaire about satisfaction with the procedure before discharge.

1. **Follow-up Period <Visit 3, 4>**

■ Visit 3 (1 month after treatment)

Visit the clinic and perform the following tests

■ Measurement of uterus size (MRI)

- Ultrasound test
- Blood test and FSH test
- Completion of questionnaire: Dysmenorrhea Relief and Dysmenorrhea/Menorrhagia Scores, Quality of Life (SF36-v2, UFS-QoL)

ALPINION Medical Systems Co., Ltd. 26/45


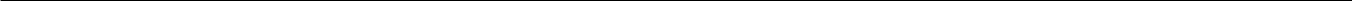

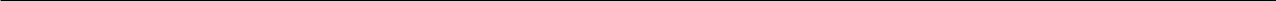


Protocol No. APM-03 Version 1.10/Date 20180103


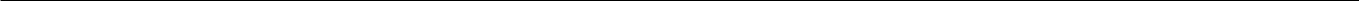


■ Adverse event/Serious adverse event check

- Concomitant drugs check

■ Visit 4 (3 months after treatment)

Visit the clinic and perform the following tests

■ Measurement of uterus size (MRI)

- Ultrasound test
- Pregnancy test
- Completion of questionnaire: Dysmenorrhea Relief and Dysmenorrhea/Menorrhagia Scores, Quality of Life (SF36-v2, UFS-QoL)
- Adverse event/Serious adverse event check
- Concomitant drugs check

**11.6.3. Concomitant Therapy**

Drugs that may affect the treatment of adenomyosis (see Contraindicated Drugs below) or alternative treatments are prohibited. All drugs already being taken due to underlying diseases are recorded at the time of Screening. Only the drugs added thereafter are recorded in the CRF. If any of the above contraindicated drugs or alternative treatments were received during the clinical trial period, they will be withdrawn from the study and the reason for withdrawal should be recorded.

However, even if it is a contraindicated drug, if a one-time administration is necessary regardless of this study, it can be used within the extent that does not affect the procedure result at the discretion of the investigator and the related information is recorded in the concomitant drugs section of the CRF.

Subjects are administered midazolam and fentanyl for sedation prior to the procedure. The dosage of midazolam and fentanyl is determined by the investigator in consideration of the age and clinical condition of the subject, but generally midazolam 3mg/3ml or 5mg/5ml, and fentanyl 100mcg/2ml are administered. At the discretion of the investigator, analgesics, sedatives and local anesthetics may be administered according to the standard procedure of the institution in consideration of the subject's condition.

**Contraindicated Drugs**

Estrogen (including combined equine estrogen), progesterone, steroids (including corticosteroids), oral contraceptives, etc.

**11.6.4. Study Budget**

Expenses paid by the subject before being referred to this clinical trial are applied retroactively and are not compensated, and expenses incurred after participating in the clinical trial (electrocardiogram, laboratory test, pregnancy test, MRI, U/S, US-HIFU procedure), etc. will be paid by the investigator. In addition, in relation to the time required for participation in this clinical trial, compensation will be made by direct bank transfer in the amount of KRW 100,000 for those residing in the area near the site every visit (Visit 3 and 4), and KRW 200,000 for those residing outside the area of the site. Payment details will be managed through the subject's compensation payment log. Compensation for subjects will not be conditional on their participation in the clinical trial to the end.

ALPINION Medical Systems Co., Ltd. 27/45


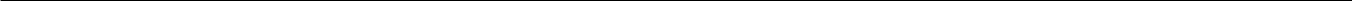

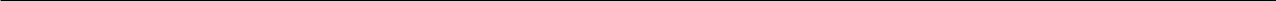


Protocol No. APM-03 Version 1.10/Date 20180103


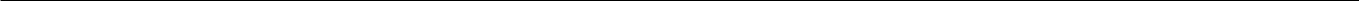


1. **Study Assessment**

**12.1. Efficacy Assessment**

**12.1.1. Primary Efficacy Endpoint**

■ Percentage Improvement in Dysmenorrhea Relief (%) - 3 months after treatment

**12.1.2. Secondary Efficacy Endpoint**

■ Percentage Improvement in Dysmenorrhea Relief (%) - 1 month after treatment

■ Dysmenorrhea Score

■ Menorrhagia Score

■ Measurement of Quality of Life

- SF36-v2
- UFS-QoL
- SSS
- Uterus size (cm^3^)
- Satisfaction with the procedure

**12.2. Assessment Criteria and Assessment Method**

<Primary Efficacy Assessment Criteria>

- Percentage Improvement in Dysmenorrhea Relief (%)
  - Definition of dysmenorrhea relief

If the degree of menstrual pain relief corresponds to Minor, Partial, or Complete on the Dysmenorrhea Relief Score

Dysmenorrhea Relief Score

- - Complete relief

② Partial relief

③ Minor relief

④ Ineffective

⑤ Exacerbated pain

- Definition of Percentage Improvement in Dysmenorrhea Relief

Proportion of subjects who met the definition of improvement in dysmenorrhea relief above at 3 months after the procedure

ALPINION Medical Systems Co., Ltd. 28/45


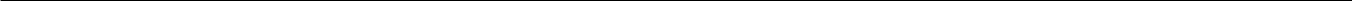

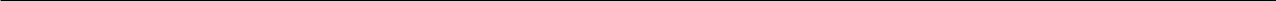


Protocol No. APM-03 Version 1.10/Date 20180103


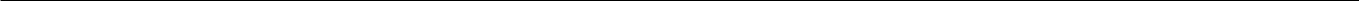


<Secondary Efficacy Endpoint>

■ Percentage Improvement in Dysmenorrhea Relief (%) - 1 month after treatment

Evaluate the Percentage Improvement in Dysmenorrhea Relief (%) 1 month after the procedure using the same method as that for the primary efficacy endpoint.

■ Dysmenorrhea Score

Subjects directly evaluate dysmenorrhea symptoms on a 5-point scale as shown below before procedure and at 1 and 3 months after the procedure.

Dysmenorrhea Score

1 = Not at all (no symptoms)

2 = A little bit (slight symptoms)

3 = Somewhat (few symptoms)

4 = A great deal (considerable symptoms)

5 = A very great deal (a lot of symptoms)

■ Menorrhagia Score

Subjects directly evaluate menorrhagia symptoms on a 5-point scale as shown below before procedure and at 1 and 3 months after the procedure.

Menorrhagia Score

1 = Not at all (no symptoms)

2 = A little bit (slight symptoms)

3 = Somewhat (few symptoms)

4 = A great deal (considerable symptoms)

5 = A very great deal (a lot of symptoms)

■ Measurement of Quality of Life

Using the questionnaire filled out by the subject, the changes at 1 month and 3 months after the procedure are compared and evaluated before and after the procedure.

- SF36-v2: This is a questionnaire to evaluate the overall quality of life, and consists of 36 questions in 8 domains including physical functioning, role limitation-physical, bodily pain, general health, vitality, social functioning, role limitation-emotional, mental health, and other changes in health condition, etc.
- UFS-QOL: As a questionnaire to evaluate the quality of life related to the symptoms of uterine fibroids, it consists of an 8-item Symptom Severity Score and a 29-item Quality of Life questionnaire.
- SSS (Symptom Severity Score): This is included in the UFS-QOL questionnaire but the severity of symptoms due to uterine fibroids will be evaluated separately.
- Uterus size (cm^3^)

Compare the overall uterus size immediately after the procedure, at 1 month and 3 months after the procedure.

ALPINION Medical Systems Co., Ltd. 29/45


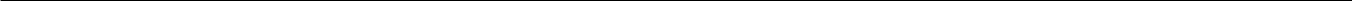

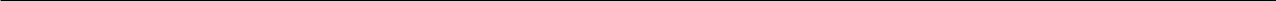


Protocol No. APM-03 Version 1.10/Date 20180103


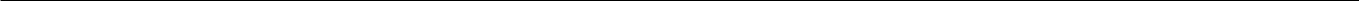


- Satisfaction with the procedure

The pain felt by the subject during the procedure and willingness to reuse depending on the satisfaction on the procedure is evaluated through a questionnaire on a 5-point scale.

**12.3. Safety Assessment**

**12.3.1. Safety Endpoints**

All adverse events that occurred to subjects during the clinical trial period

**12.3.2. Assessment Criteria and Assessment Method**

In this clinical trial, adverse events are all undesirable medical findings that cause symptoms not observed before the start of the clinical trial are classified as adverse events. In accordance with the definitions and criteria described in the section ‘15. Assessment Criteria, Assessment Method and Reporting of Safety Including Side Effects’, expected side effects are also classified as adverse events. The severity of adverse events is classified into mild, moderate, and severe, and the use of terms is based on MedDRA’s ‘Preferred term’ and ‘System organ class’.

1. **Data Collection and Statistical Analysis**

**13.1. General Considerations**

All data measured and recorded in this study will be summarized according to the definition of the appropriate analysis sets defined in 14.2. Summary statistics are based on mean ± standard deviation for continuous data and frequency (fraction) for categorical data, and more detailed summary statistics will be presented if necessary.

**13.2. Definition of Analysis Sets**

Efficacy analysis in this clinical trial defines FAS (Full Analysis Set) as the main analysis set, and conducts assessment based on it. As an auxiliary analysis, the same assessment is repeated for the PP analysis set (Per-Protocol Analysis Set). Safety assessment is conducted on all subjects who participated in the study after consent to participate in the study and who received treatment with the investigational medical device.

**13.2.1. Efficacy Analysis Set**

- FAS (Full Analysis Set): As the main analysis set for the assessment of efficacy in this study, it is defined as any subject who participates in the study after consenting to participation, receives the application of investigational medical device, and has at least one valid evaluable data.

ALPINION Medical Systems Co., Ltd. 30/45


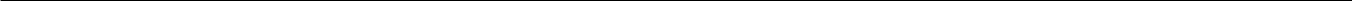


Protocol No. APM-03 Version 1.10/Date 20180103

- PP analysis set (Per Protocol Analysis Set): It is defined as all subjects who participated in the study after consenting to participate in the study, received the investigational medical device, and completed the clinical study according to the study plan without serious protocol violations until the end of the study. Those who received drugs or alternative treatments that affect efficacy during the clinical trial period are excluded from the PP analysis set.

1. *Major protocol violations to be excluded from the analysis set*
2. Those who received drugs or alternative treatments that affect efficacy during the clinical trial period

**13.2.2. Safety Analysis Set**

■ All subjects who participated in the study after consenting to participate in the study and received treatment with the investigational medical device are included.

**13.2.3. Handling Missing Data (missing values)**

If a missing value occurs in the efficacy endpoints, the variable is excluded from the analysis.

**13.3. Statistical Analysis Method**

**13.3.1. Analysis of General Items**

Data for continuous measurements will be summarized in tables showing mean, standard deviation, median, minimum, maximum and number of subjects. Data for categorical measures will be summarized in tables showing numbers and fractions.

All statistical analyzes will be performed using the SAS statistical program.

**13.3.2. Primary Efficacy Analysis**

■ Percentage Improvement in Dysmenorrhea Relief (%) - 3 months after treatment

The success rate of dysmenorrhea relief will be confirmed based on the following hypothesis.

H_0_: P_A_ - P_0_ ≤ - δ vs H_1_: P_A_ - P_0_> - δ

- - P_A_: Expected success rate of the investigational medical device
  - P_0_: Reference success rate (90%)
  - δ (>0): Non-inferiority threshold (12.3%)

The hypothesis can be expressed as H_0_: P_A_ ≤ 0.777 vs H_1_: P_A_  > 0.777

Therefore, if the lower limit of the 97.5% one-tailed confidence interval of the measured improvement rate (P_A_) exceeds 0.777, this shows efficacy of the clinical trial.

ALPINION Medical Systems Co., Ltd. 31/45

Protocol No. APM-03 Version 1.10/Date 20180103

**13.3.3. Secondary Efficacy Analysis**

The assessment of the secondary outcome variables considered in this study will be performed at 1 month and 3 months after the procedure, respectively. The ‘satisfaction with the procedure’ is assessed on the day of the procedure.

■ Percentage Improvement in Dysmenorrhea Relief (%) - 1 month after treatment

The same Improvement in Dysmenorrhea Relief as the primary endpoint is obtained at 1 month and a 95% confidence interval is presented.

■ Dysmenorrhea Score - Before procedure, at Months 1 and 3

The change in score at 1 and 3 months after the procedure compared to before the procedure is compared and evaluated using the Wilcoxon signed rank test.

■ Menorrhagia Score - Before procedure, at Months 1 and 3

The change in score at 1 and 3 months after the procedure compared to before the procedure is compared and evaluated using the Wilcoxon signed rank test.

■ Measurement of quality of life (SF36-v2, UFS-QOL, SSS)

Comparison of total and individual item scores for each questionnaire before and after surgery will be analyzed by performing paired t-test or Wilcoxon signed-rank test.

- Uterus size (cm^3^)

The size of the uterus at 1 month and 3 months after the procedure compared to before the procedure will be compared and evaluated using the paired t-test or Wilcoxon signed rank test.

- Satisfaction with the procedure

The pain and satisfaction scores felt by the subjects during the procedure are summarized as mean and standard deviation.

**13.3.4. Safety Analysis**

Safety assessment is performed based on all adverse reactions, vital signs, blood tests, etc. collected from the subject.

All the above safety variable data collected up to 3 months after the procedure are presented for each time point at which each safety endpoint was measured and for each subject, and summary statistics are presented. Adverse events collected from subjects will be coded using MedDRA. Adverse events, adverse medical device reactions, SAEs, deaths, adverse events that resulted in clinical trial discontinuation, and the number of subjects who exhibited each adverse reaction will be summarized by the system organ class (SOC), preferred term (PT) and maximum severity.

1. **Clinical Trial Discontinuation and Dropout Criteria**

If participation is withdrawn after consent to participate in the clinical trial, or if participation in the clinical trial is judged to affect the safety of the subject under the judgment of the principal investigator, participation in the clinical trial may be suspended or the subject may be withdrawn from participation.

ALPINION Medical Systems Co., Ltd. 32/45

Protocol No. APM-03 Version 1.10/Date 20180103

**14.1. Discontinuation Criteria**

① If the circumstances observed during the clinical trial make it unreasonable to proceed with the clinical trial, the clinical principal investigator may suspend the clinical trial, must immediately notify the Institutional Review Board of this fact and submit a statement of reason for early termination or suspension.

② The clinical trial may be stopped in the event of a life-threatening serious adverse reaction/adverse medical device or to treat the adverse reaction that has occurred.

**14.2. Dropout Criteria**

① When the subject or legal representative requests to stop participating in the clinical trial

② In case of concomitant use of surgery, drug, or medical device that may affect safety and efficacy assessment

③ In case a serious adverse reaction occurs and it is impossible to continue participation in the clinical trial

④ If treatment is not performed properly

⑤ If the subject does not comply with the instructions of the investigator or does not comply with the matters presented in the consent form, which affects the assessment of efficacy

⑥ In case the subject dies due to reasons not related to the clinical trial

⑦ If the clinical trial manager determines that there is a problem with the clinical trial

**14.3. Handling Discontinuations**

① If the clinical trial is suspended, the reason for the suspension and the data related to the clinical trial conducted before the suspension are recorded and stored, and a statement of reason is submitted.

② Appropriate measures and follow-up should be made when the clinical trial is discontinued.

③ All discontinued subjects will be included in the safety analysis and will be excluded from the efficacy assessment.

**14.4. Handling Dropouts**

① In case of dropping out of a clinical trial, record and keep the reasons for withdrawal and data related to the clinical trial conducted before dropping out, and submit a statement of reason to the Institutional Review Board.

② All data collected until the subject's dropout will be included in the safety analysis and will be excluded from the efficacy assessment.

1. **Assessment Criteria, Assessment Method and Reporting of Safety Including Side Effects**

ALPINION Medical Systems Co., Ltd. 33/45

Protocol No. APM-03 Version 1.10/Date 20180103

**15.1. Definition of Adverse Event**

- “Adverse Event (AE)” refers to any undesirable and unintended signs (signs, e.g., abnormalities in laboratory test values), symptoms, and diseases occurring in subjects during clinical trials, and it does not necessarily have to have a causal relationship with the investigational medical device.

- - “Adverse Device Effect (ADE)” refers to any harmful and unintended reaction caused by an investigational medical device, and a causal relationship with the investigational medical device cannot be excluded.
- “Unexpected Adverse Device Effect” refers to a difference in the aspect of an adverse medical device reaction or the degree of harm in light of available medical device-related information (For example, from that of Investigator’s Brochure or attachments of medical device).

**15.2. Definition of Serious Adverse Events/Adverse Medical Device Reactions**

Serious adverse event/Adverse medical device reaction refers to a case that falls under the following among the adverse reactions caused by medical devices used in clinical trials.

① Results in death or is life-threatening

② Requires hospitalization or prolongation of existing hospitalization

③ Results in persistent or significant disability or impairment

④ Results in congenital malformation or anomaly

**15.3. Assessment of Adverse Events**

**15.3.1. Severity Assessment**

If an adverse event occurs, it should be reported according to the following severity assessment criteria.

① Mild

Cases that do not interfere with the subject's normal daily life (function), cause minimal discomfort, and are easily tolerated by the subject

② Moderate

In case of causing discomfort that significantly interferes with the normal daily life (function) of the subject ③ Severe

When the subject's normal daily life (function) is impossible

**15.3.2. Assessment of Causality with Investigational Medical Device**

When an adverse event occurs, the investigator assesses whether it is related to the investigational medical device according to the following criteria, and the investigator's opinion is described.

ALPINION Medical Systems Co., Ltd. 34/45

Protocol No. APM-03 Version 1.10/Date 20180103

① Definite

② Probable

③ Possible

④ Possibly not

⑤ Definitely not

⑥ Unknown

**15.4. Adverse Events Assessment Criteria**

In this clinical trial, adverse events are all undesirable medical findings that cause symptoms not observed before the start of the clinical trial are classified as adverse events. Expected side effects are also classified as adverse events, and the severity of adverse reactions is classified into mild, moderate, and severe, and they are reported using the ‘Preferred term’ and ‘System organ class’ of MedDRA.

**15.5. Expected Adverse Events and Precautions**

**15.5.1. Expected Adverse Events**

- Pain
- Nausea
- Vomiting
- Abdominal tenderness
- Edema
- Abdominal cramping
- 1-3 degree burns
- Internal tissue thermal damage
- Leg and hip pain
- Vaginal bleeding more than before treatment
- Sciatic nerve injury
- Abdominal and pelvic organ damage (bladder, uterus, intestine, etc.)
- Pain that does not respond to drugs
- Urinary tract infection
- Urination disorders
- Fever due to infection
- Hypersensitivity reactions to the use of contrast agents (dizziness, nausea, vomiting, itching, urticaria, burning sensation, blood pressure drop, heartbeat abnormality, dyspnea, kidney disease, acute renal failure, etc.)
- congestive changes in the abdominal muscles on MR examination

(Limited to cases where there is a scar on the abdomen and the procedure is performed after applying a scar patch.)

**15.5.2. Precautions for Use**

ALPINION Medical Systems Co., Ltd. 35/45

Protocol No. APM-03 Version 1.10/Date 20180103

Subject Safety Information

① Correctly identify and enter subject data.

② Do not use the system until you are familiar with its operation.

③ To prevent overheating of the image transducer, the image should be fixed when not viewing the image.

④ Ensure that no acoustic output is emitted from the system when the image transducer is not in use. Otherwise, the image transducer may overheat.

⑤ When the system is not in use, the image transducer must be fixed or the sound output must be turned off.

⑥ Do not use the system with a defibrillator. This system does not contain any ECG components with defibrillator protection.

⑦ Make sure that the disinfectant does not come into contact with the subject. If the disinfectant comes into contact with the skin or mucous membrane of a subject, it may cause infection.

- Precautions for Applications
  - To avoid system damage and serious subject injury, do not use damaged or defective image transducers.

② Do not bend or pull the image transducer cable as it may damage the image transducer.

③ Use only approved coupling gels.Use of non-approved gels may damage the image transducer and void the warranty.

④ Do not drop the image transducer.Always store the image transducer in a safe place when not in use.

⑤ Use the image transducer with care, as using a damaged or defective image transducer may cause unexpected electric shock.

⑥ Ensure that no sound output is emitted from the system when the image transducer is not in use.

Otherwise, the image transducer may overheat.

⑦ When the system is not in use, the image transducer must be cooled or the sound output must be turned off.

⑧ Always inspect the image transducer used for sharp edges or rough surfaces to avoid serious subject injury.

⑨ Proper cleaning and sterilization of the image transducer is essential to prevent disease transmission. Infection control procedures should be followed.

⑩ Do not use an image transducer sheath which has expired.

⑪ Using a lubricated condom as an envelope may damage the image transducer.

⑫ Do not let the coupling gel get into the eye (or the subject's eye).

⑬ If the gel gets into your eyes, rinse well with clean water.

**15.6. Reporting Adverse Events**

**15.6.1. Adverse Event Training**

ALPINION Medical Systems Co., Ltd. 36/45

Protocol No. APM-03 Version 1.10/Date 20180103

The principal investigator educates sub-investigators and subjects or their representatives on any adverse events that may occur after surgery or use of investigational medical device. Training is provided to report all phenomena that appear after use through the reporting form specified within the time limit specified in the protocol.

**15.6.2. Recording Expected Adverse Events**

When an expected adverse event occurs, the investigator records the following in the CRF.

- Name of adverse event
- Date of onset and resolution
- Severity
- Causal relationship with the investigational medical device
- Treatment details and outcome

**15.6.3. Reporting Serious Adverse Events/Medical Device Reactions**

The investigator shall record all serious adverse events/adverse medical device reactions that occurred during the clinical trial period on a serious adverse event record sheet, and report it to the sponsor within 24 hours regardless of the relationship with the investigational medical device. In this case, in order to protect the ity of the subject's identity, the subject identification code is used in place of the subject's personal information.

In addition, events that suggest significant risks, contraindications, side effects, and precautions that the investigator considers serious or that may be related to the use of investigational medical device are also recorded as serious adverse events/adverse medical device reactions and reported immediately to the sponsor.

When reporting a death case, the investigator must submit additional information such as an autopsy report (applicable only when an autopsy has been performed) and a death certificate to the sponsor and the IRB.

The sponsor shall report serious and unexpected adverse events (including adverse medical device reactions, serious adverse events/adverse medical device reactions) to the Institutional Review Board and the Minister of Food and Drug Safety as soon as possible within the period specified in each of the following subparagraphs.

1. For death or life-threatening cases, the principal investigator must report additional information within 7 days of receiving the report or becoming aware this fact, and in this case, detailed information must be reported within 8 days from the date of the initial report.
2. Any other serious or unexpected adverse events should be reported within 15 days of the principal investigator receiving the report or becoming aware this fact.
3. When a sponsor wishes to report an adverse medical device reaction to the Minister of Food and Drug Safety in accordance with 1) above, it must be submitted in the adverse medical device reaction report according to Form of Attachment No. 36 of the KGCP.

In relation to reporting serious adverse events and adverse medical device reactions, additional safety information should be periodically reported until the relevant adverse reaction is resolved (disappearance of the relevant adverse reaction or lost to follow-up, etc.).

ALPINION Medical Systems Co., Ltd. 37/45

Protocol No. APM-03 Version 1.10/Date 20180103

1. **Measures for Subject Safety Protection**

**16.1. Korean Good Clinical Practice (KGCP) for Medical Device and Declaration of Helsinki**

The procedures stipulated in this protocol are designed to ensure that investigators comply with the basic spirit of the ICH-GCP and the Declaration of Helsinki in conducting, evaluating and recording the results of this clinical trial. This clinical trial will also be conducted in accordance with domestic regulations (Korean Good Clinical Practice, KGCP).

**16.2. Institutional Review Board (IRB)**

Before starting the clinical trial, the investigator must submit the clinical trial protocol, consent form, data and related documents related to subject recruitment (e.g., advertisement) to the Institutional Review Board for review and approval. If there are any changes in the protocol that require approval from the IRB and the MFDs, the changes in the clinical trial protocol will not be applied until the IRB reviews and approves the revised clinical trial protocol and the amended informed consent form (if applicable). Amendments to the clinical trial protocol to eliminate the immediate risk factors posed to subjects may be applied immediately, granted that they are notified to the MFDS and the IRB immediately with a request for approval. Changes in the protocol related to administrative procedures, such as change of monitor staff, change of investigator, change of emergency contact phone number, etc., can be applied immediately even before IRB approval.

The investigator must provide the clinical trial result report, latest information, and other information (e.g., safety update) to the Institutional Review Board in accordance with relevant regulations or hospital procedures.

**16.3. Subject ICF**

Written informed consent must be obtained from each subject (or their legal representative) before screening/baseline assessments for the clinical trial are conducted.The clinical trial investigator should explain all the details related to the trial in detail to the patients who have satisfied both the inclusion and exclusion criteria and their caregivers before starting the trial, and give them sufficient time to learn about all foreseeable outcomes. A copy of the signed consent form is kept by the subject and the original is kept by the investigator.

The clinical trial investigator must prepare and keep a list of all subjects who have consented to participate in the clinical trial and submit it to the clinical trial sponsor.

Subjects should be informed of the level of disclosure and the fact that their study-related data will be used by the sponsor in accordance with clinical trial regulations. In addition, subjects should be informed of the fact that the subject’s medical records may be reviewed by clinical trial monitor personnel or auditors, IRBs, or public health inspectors.

When the clinical trial protocol is revised, the informed consent form and the information sheet may be revised to reflect the changes in the protocol. If the informed consent form and the information sheet are revised, they must be reviewed and approved by the IRB, and the changes must be explained to the newly enrolled and currently participating subjects, and the revised consent form must be signed.

ALPINION Medical Systems Co., Ltd. 38/45

Protocol No. APM-03 Version 1.10/Date 20180103

**16.4. Indemnification Policy**

If the subject is injured due to the use of the medical device or clinical procedure that he/she would not have received if he/she had not participated in this clinical trial, he/she will receive all treatment according to the standard procedures of the hospital. In case of damage related to the clinical trial, the cost will be borne according to the indemnification policy and the clinical trial insurance policy.

**16.5. Measures for Subject Safety Protection**

When new information that may affect the continuation of this clinical trial is obtained, the information will be provided to the subject or his/her representative in a timely manner, and the investigator will discuss with the subject whether to continue participating in the clinical trial.

**16.6. Care and Treatment Criteria of Subjects After Clinical Trial**

If the subject drops out of the clinical trial due to an adverse event or if there are side effects and residual symptoms due to the adverse event even after the completion of the clinical trial, the subject will receive adequate treatment until recovery by taking appropriate medical measures according to the standard medical guidelines of the hospital. Subjects who have completed the clinical trial without adverse events will be subject to general clinical observation according to standard hospital procedures after the clinical trial.

**16.7. Clinical Trial Site**

The head of the clinical trial site shall have the clinical laboratory, facilities, and professional manpower necessary for the conduct of the relevant clinical trial, and shall ensure that the relevant clinical trial can be properly conducted, such as taking necessary measures in case of emergency.

**16.8. Investigators**

① Investigators refer to the principal investigator, sub-investigators, and clinical trial coordinators. Investigators must conduct clinical trials in accordance with the clinical trial protocol agreed with the sponsor and approved by the Institutional Review Board and the Minister of Food and Drug Safety.

② During or after the clinical trial, the investigator must take measures so that the subject can receive appropriate medical treatment for all adverse events that occurred in the clinical trial, including abnormalities in clinically meaningful laboratory tests. If medical treatment is required for a subject's concomitant disease, which the investigator has learned, the subject should be informed of this.

- According to the section ‘15.6.3. Reporting Serious Adverse Events/Medical Device Reactions’, the sub-investigator has the responsibility to recognize the adverse event and report it to the principal investigator, and the principal investigator is obliged to report the adverse event reported from the sub-investigator to the sponsor.

ALPINION Medical Systems Co., Ltd. 39/45

Protocol No. APM-03 Version 1.9/Date 20170829

④ The investigator accurately analyzes and understands the clinical trial plan, and actively responds to the subject's problems.

**16.9. Sponsor**

① A person who has responsibilities related to the planning, management, and finance of clinical research, usually refers to a medical device manufacturer (including importers) in the case of medical device clinical trials.

② Must ensure that clinical trial subjects, test methods, and the format and contents of CRFs are made in accordance with the procedures of the clinical trial protocol.

③ The sponsor’s inspection plan and procedure should be determined according to the importance of the clinical trial, the number of subjects, the type and complexity of the clinical trial, the degree of potential risk to the subjects, and problems in the clinical trial that have already been identified.

1. **Other Matters Necessary for Safe and Scientific Clinical Trial**

**17.1. ity**

**17.1.1. Data**

The investigator must keep the ity of all information related to the protocol provided by the sponsor or clinical trial monitoring staff. However, an exception is made when the IRB, the subject, or the public health authority requests disclosure in accordance with laws or related regulations.

**17.1.2. ity of Subjects**

The anonymity of subjects participating in the clinical trial must be guaranteed. Identification of the subject should be made with the subject's initials and the subject number specified in the CRF and other data submitted to the clinical trial monitoring staff. Inform the subject that all study data will be stored on a computer and will be treated in strict ity. The signed informed consent form will be kept by the principal investigator. The principal investigator should keep a list of subject numbers and subject names so that the records can be retrieved later. The informed consent form and the subject list are kept for 3 years.

Data for subject identification must be kept strictly by the investigator. However, there will be exceptions when it is necessary to be audited by the health authorities, clinical trial monitoring staff, sponsor, or designated representatives.

**17.2. Protocol Compliance and Protocol Amendment**

Neither the principal investigator nor the sponsor may change the contents of this study protocol during the trial without the consent of the other party.

ALPINION Medical Systems Co., Ltd. 40/45

Protocol No. APM-03 Version 1.9/Date 20170829

Any changes to the study protocol should be discussed with the sponsor, and the amended protocol should be prepared by the sponsor. The investigator should not apply the amended contents before obtaining review approval from the Institutional Review Board for such a protocol amendment, except in the case of immediate prevention of harm to the subject. Significant protocol violations should be recorded in the CRF.

If any modifications or changes to this protocol are applied prior to obtaining Institutional Review Board approval in order to prevent immediate harm to the subject, such modifications or changes should be submitted to the Institutional Review Board immediately (for later review and approval) and the Ministry of Food and Drug Safety (if required by related regulations).

Significant changes to the protocol include changes that affect the safety of subjects, changes in the scope of the study, changes in the scientific quality of the clinical trial, changes in experimental design, changes in endpoint(s), changes in the number of subjects, changes in the subject selection criteria. These changes must be recorded and provided as data to justify the changes.

The sponsor must prepare these changes in the revised clinical trial protocol, which can only be implemented with the joint approval of the sponsor and the investigator. The revised clinical trial protocol must be approved by the relevant IRB or relevant authorities before implementation.

For protocol amendment procedure, changes to the original approval version will be submitted to the approving authority in parallel with the IRB approval process. If the amended clinical trial protocol requires ICF amendment, it must be approved by the IRB.

An urgent deviation from the protocol to rule out an obvious and imminent risk of a particular subject participating in a clinical trial is considered critical for the safety and well-being of the subject and can only be implemented in the case of that particular subject.

If the protocol amendments are minor, it is sufficient for the investigator to notify the Institutional Review Board. However, if there is a substantial change in the trial design or an increased risk to the subject, 1) the ICF must be modified and submitted to the Institutional Review Board for review approval; 2) if this change affects the subjects, consent must be obtained again from the subjects who have already been recruited for the study; 3) consent must be obtained using the new consent form from the newly recruited subjects.

**17.3. Clinical Trial Monitoring**

Prior to the start of the clinical trial, an investigator meeting or clinical trial initiation meeting will be held with the sponsor, principal investigator, and the person in charge of the CRO for this clinical trial. In this meeting, detailed discussions will be held on clinical trial protocol, clinical trial procedure implementation, case record form completion, and testing method. Investigators who are unable to attend these meetings or gatherings, or who will participate in the clinical trial later, should receive appropriate training by the sponsor, the principal investigator, or a person delegated thereto.

The sponsor and the CRO should properly secure relevant guidelines and data at the study site, and designate a monitoring staff in charge of this clinical trial to conduct monitoring for data integrity before the start of the clinical trial and during the trial period.

ALPINION Medical Systems Co., Ltd. 41/45

Protocol No. APM-03 Version 1.9/Date 20170829

In order to ensure that the study is conducted in accordance with the KGCP and the study data can be recognized at the time of registration domestically and abroad, monitoring and auditing can be conducted by inquiring an external organization. The monitoring staff explains the monitoring plan to the investigator before starting the clinical trial, and during monitoring, verifies that the investigator is performing the clinical trial in accordance with the clinical trial protocol and related regulations. Clinical trial monitoring staff routinely contact investigators and are authorized to monitor various records of the clinical trial.

It is the responsibility of the clinical trial monitoring staff to monitor the CRF regularly throughout the clinical trial period to verify the completeness, consistency and accuracy of the recorded data and to prove that the recorded data is faithful to the clinical trial protocol. The findings should be appropriately discussed with the investigator. The investigator should properly inform the clinical trial monitoring staff of any findings discovered during the clinical trial process and cooperate in monitoring activities.

**17.4. Recording and Use of Study Results**

**17.4.1. Case Report Forms and Source Documents**

All information required in the clinical trial should be recorded on the relevant CRF. In addition, the investigator must sign the investigator signature line in each CRF to ensure the accuracy of the data.

The investigator should allow direct access to source data and source documents during monitoring, inspection, IRB review, and inspection related to the clinical trial. Each subject's original CRF will be checked by clinical trial monitoring staff against the source documents at the study site.

The principal investigator is responsible for maintaining and providing essential clinical trial documentation. Essential clinical trial documents are documents that enable individual or overall assessment of the performance of the clinical trial and the quality of data obtained therefrom. Essential clinical trial documents include source documents, which include hospital records, medical records, clinical laboratory test results, subject diaries, and various test papers and records kept in pharmacies, pathological laboratories, and medical/technical departments involved in the clinical trial.

Source documents, CRFs, and other clinical trial-related documents should be kept at the study site, and the original copies of the CRFs that have been monitored are retrieved by the principal investigator while the copy is kept at the study site. The investigator keeps all records related to the clinical trial until the MFDS completes the inspection. Once the inspection is completed, the investigator keeps it until the deadline set by the sponsor.

**17.4.2. Retention of Clinical Study Data**

All CRFs and provided data and management records should be kept for 3 years from the date of marketing approval of this product. No clinical research data may be destroyed or transferred to another location without the prior written consent of the principal investigator. If an investigator is excluded from the clinical trial due to relocation, retirement, or other reasons, the principal investigator should be informed and agreed to an appropriate solution.

ALPINION Medical Systems Co., Ltd. 42/45

Protocol No. APM-03 Version 1.9/Date 20170829

The investigator shall preserve investigational medical device usage records, copy of CRFs, and source data related to the clinical trial for a period of 3 years according to domestic regulations.

**17.4.3. Use of Study Results**

By signing this clinical trial protocol, the investigator agrees to use the results of this trial for the purposes of registration, presentation, and provision of information for medical device experts. The principal investigator has the right to review the presentation before publishing the results of this clinical trial in academic journals.

After completion of the clinical trial, the investigator and sponsor review all findings, analyses, and reports to develop an up-to-date strategy for publication. All publications should be prepared in cooperation between the investigator and the sponsor.

Information generated during the clinical trial is the property of the sponsor and should not be published without the sponsor's written permission.

**17.5. Agreement Between Sponsor and Head of Clinical Trial Site**

① The sponsor must conclude a clinical trial contract with the head of a clinical trial site in writing, and in the case of conducting a multi-center clinical trial, the sponsor can collectively sign a contract with the head of one clinical trial site.

② The clinical trial protocol should contain matters related to the finance of the clinical trial, such as the size and payment method of study budget, early termination and return of unused study budget in case of discontinuation of the study, matters concerning delegation and division of duties, and obligations of the sponsor and the head of the clinical trial site.

**17.6. CV of Principal Investigator**

① In order to properly conduct the clinical trial, the investigator shall have education, training, and experience necessary for conducting the clinical trial as stipulated in the standard operating guidelines of the study site.

② The investigator should know in detail how to properly use the investigational medical device described in the clinical trial protocol, the Investigator’s Brochure, and other medical device-related information provided by the sponsor.

③ The investigator must know and comply with the relevant laws in detail.

④ The principal investigator and the head of the clinical trial site shall follow the sponsor's monitoring and inspection.

⑤ If important clinical trial related tasks are delegated to the sub-investigator, the principal investigator must secure and maintain the list of sub-investigators.

**17.7. Use and Management of Investigational Medical Device**

① The investigational medical device shall be managed by a person designated by the head of the relevant clinical trial site. Investigational medical device should be handled and stored as described in the description, and the phrase “for clinical trial use” should be on it. Investigational medical device manager shall perform tasks such as acceptance, inventory management, and return of medical devices used in the clinical trial, and maintain related records.

**17.8. Supply and Handling of Investigational Medical Device**

ALPINION Medical Systems Co., Ltd. 43/45

Protocol No. APM-03 Version 1.9/Date 20170829

① The sponsor shall not supply investigational medical device to the manager, etc., before obtaining approval from the Institutional Review Board and the Minister of Food and Drug Safety.

② The sponsor must have documented procedures for how the manager, etc. handles and stores investigational medical device. These procedures include methods for proper and safe acceptance, handling, storage, and return of unused investigational medical device from subjects and the sponsor.

③ Investigational medical device shall be supplied in a timely manner, and records of supply to clinical trial sites, receipt by the clinical trial sites, returns from clinical trial sites, and disposal records shall be maintained.

④ The sponsor shall establish and document the recovery system of investigational medical device due to problems such as malfunctions, termination of clinical trials or expiration of the usage period, etc.

1. **References**
2. Xin Xhang et al. Effective ablation therapy of adenomyosis with ultrasound-guided high-intensity focused ultrasound. Int J Gynaecol Obstet. 2014 Mar;124(3):207-11.
3. Fan TY et al. Feasibility of MRI-guided high intensity focused ultrasound treatment for adenomyosis. Eur J Radiol. 2012 Nov;81(11):3624-30.
4. Zhou M et al. Ultrasound-guided high-intensity focused ultrasound ablation for adenomyosis: the clinical experience of a single center. Fertil Steril. 2011 Mar 1;95(3):900-5
5. Fukunishi H et al. Early results of magnetic resonance-guided focused ultrasound surgery of adenomyosis: analysis of 20 cases. J Minim Invasive Gynecol. 2008 Sep-Oct;15(5):571-9.
6. Zhang L, Chen WZ, Liu YJ, et al. Feasibility of magnetic resonance imaging guided high intensity focused ultrasound therapy for ablating uterine fibroids in patients with bowel lies anterior to uterus. European Journal of Radiology 2010;73:396–403.
7. Tempany CM, Stewart EA, McDannold N, Quade BJ, Jolesz FA, Hynynen K. MR imaging-guided focused ultrasound surgery of uterine leiomyomas: a feasibility study. Radiology 2003;226:897–905.
8. LeBlang SD, Hoctor K, Steinberg FL. Leiomyoma shrinkage after MRI-guided focused ultrasound treatment: report of 80 patients. American Journal of Roentgenology 2010;194:274

–80.

1. Buttram VC Jr, Reiter RC. Uterine leiomyomata: etiology, symptomatology, and management. Fertil Steril 1981;36:433-45.
2. Hong Jin Hwa et al. Uterine Artery Embolization for Leiomyoma. Obstetrics & Gynecology Scienc 47(2004) 481-486
3. Seung Yol Lee et al. Clinical Review on 93 Cases of Laparoscopic Myomectomy. Obstetrics & Gynecology Science 47(2004) 6:1107-1112
4. Eun Seop Song. Ultrasound imaging guided high intensity focused ultrasound (HIFU) may be a safe tool to ablate uterine myoma. Obstetrics & Gynecology Science 53(2009) 8:843-849.

ALPINION Medical Systems Co., Ltd. 44/45

Protocol No. APM-03 Version 1.9/Date 20170829

1. W Wang et al. Safety and efficacy of high intensity focused ultrasound ablation therapy for adnomyosis. Academic Rediology. 2009;16(11):1416-1423.

ALPINION Medical Systems Co., Ltd. 45/45
