# Supplementary material for: Optimized treatment parameter by computer simulation for high-intensity focused ultrasound treatment of uterine adenomyosis: Short-term and long-term results
Source: PLoS One. 2024 Mar 28;19(3):e0301193. doi: 10.1371/journal.pone.0301193 (PMC10977802; doi:10.1371/journal.pone.0301193)
Supplement: S3 File — (PDF) [file pone.0301193.s014.pdf]

자궁선근증 환자를 대상으로 ‘ALPIUS 900’ (US-guided HIFU System, 초음파 유도 하 고강도 집속형 초음파 수술기)의 안전성과 유효성을 평가하기 위한 전향적, 다기관, 단일군 확증 임상시험

계획서번호: APM-03
Version No.: 1.10
날짜: 2018년 01월 03일

서 명

임상시험 책임자

저는 본 임상시험용 의료기기에 대한 정보를 읽고 검토한 후 모두 이해하였으므로 아래 서명을 합니다. 또한 본 임상시험계획서를 읽고 검토하였으며, 이에 따라 임상시험을 진행하는데 동의합니다. 저는 본 임상시험을 ICH Good Clinical Practice (GCP) 기준과 적용되는 모든 관련규정에 따라 진행할 것이며, 헬싱키 선언과 임상연구윤리위원회(IRB)의 윤리기준에 따라 연구자로서의 주요 의무를 다하겠습니다.

____

임상시험기관명

______________________________ ______________________________

시험책임자 서명 날짜

< 임상시험계획서 요약 >

제목	자궁선근증 환자를 대상으로 'ALPIUS 900'(US-guided HIFU System, 초음파 유도 하 고강도 집속형 초음파 수술기)의 안전성과 유효성을 평가하기 위한 전향적, 다기관, 단일군 확증 임상시험
목적	본 임상시험은 자궁근종 적응증에 대해 제조품목허가 된 알피니언메디칼시스템(주)의 초음파 유도 하 고강도 집속형 초음파 수술기인 'ALPIUS 900(US-guided HIFU System)'의 "증상이 있는 자궁선근증(Adenomyosis)"에 대한 비침습적 자궁선근증 소작술(ablation)의 유효성과 안전성을 확인하기 위함이다. (식약처 허가용)
연구기관<br>/시험책임자	■ 서울대학교병원 / 이재영 교수(영상의학과)<br>■ 건양대학교병원 / 김철중 교수(산부인과)
임상시험용<br>의료기기를<br>관리하는 자	■ 서울대학교병원 / 박동혁 영상의학과 방사선사<br>■ 건양대학교병원 / 최다미 연구코디네이터(산부인과)
의뢰자	알피니언메디칼시스템(주), 대표이사: 최영춘<br>서울특별시 구로구 구로디지털로 26길 72, 베르디타워 1층, 6층
모니터	(주)사이넥스, 대표이사 김 영<br>(06223) 서울특별시 강남구 논현로 430 아세아타워 10층
대상질환<br>/피험자	자궁선근증 소작술이 필요한 환자
피험자 수	80명(10% 중도탈락률 고려)
임상 시험기기	'ALPIUS 900' (제허 14-3227호)<br>초음파 유도 하 고강도 집속형 초음파 수술기(US-guided HIFU)
시험설계	본 임상시험은 증상이 있는 자궁선근증 환자를 대상으로 초음파영상 유도 하 고강도 집속형 초음파 수술기인 'ALPIUS 900'의 안전성과 유효성을 평가하기 위해 계획되었으며, 전향적, 다기관, 단일군 임상시험으로 국내 2개 기관에서 총 80명을 대상으로 실시될 예정이다.
시험방법	1. 산부인과 외래를 방문하여 자궁선근증으로 임상적 진단을 받은 환자는 본 임상시험에 의뢰될 것이다.<br>☞ 자궁선근증을 최초로 진단받은 병원의 CT, MRI, 초음파 영상이나 현재 치료받고 있는 병원의 진단 영상물 또는 소견서 등을 받은 환자도 가능하다.<br>2. 자발적으로 동의서에 서명하고 선정/제외기준을 모두 만족하면 본 연구에 등록되고 피험자 번호를 배정 받게 된다.<br>3. 연구에 등록된 피험자는 시술 일정을 예약하고, 시술 당일 예정된 시술을 받는다. 시술 전 준비 사항 및 시술 과정은 기관의 표준 절차를 따른다.

	4. 시술 후 시술자는 계획된 용적과 치료 용적의 범위를 확인하기 위해 초음파와 MRI를 통해 치료 부위의 이미지를 획득하고 피부의 변화를 포함한 이상반응 여부를 관찰한다.<br>5. 회복실에서 1~2시간 경과를 관찰한 후 특별한 이상이 없으면 시술 당일 또는 그 다음 날 퇴원할 수 있다.<br>6. 시술 후 1개월 및 3개월 시점에 내원하여 유효성 및 안전성 평가를 받게 되며, 피험자의 신체에 특별한 이상이 없는 경우 임상시험을 종료한다.
임상시험 기간	식품의약품안전처로부터 임상시험계획승인을 득한 후 피험자등록기간 약 25개월 추적관찰 기간 3개월을 포함하여 약 28개월이 소요될 것으로 예상하며, 임상시험이 종료 된 후에도 데이터처리, 통계분석, 결과보고서 작성 및 IRB승인을 위해 약 3개월이 추가로 소요될 것으로 예상한다.
선정기준	다음의 선정기준을 모두 만족해야 본 임상시험에 등록될 수 있다.<br>1. 만 20세 이상 성인 여성<br>2. 폐경 이전 또는 폐경기 전후인 경우 (FSH<40mIU/ml)<br>3. MR 또는 US 영상을 통해 임상적으로 자궁선근증을 진단받은 경우<br>4. 월경통 지표 상 통증 점수가 4점 이상인 경우<br>* 월경통 지표(Dysmenorrhea Score)<br>1 = Not at all (증상이 없음)<br>2 = A little bit (증상이 조금 있음)<br>3 = Somewhat (증상이 약간 있음)<br>4 = A great deal (증상이 상당히 있음)<br>5 = A very great deal (증상이 아주 많이 있음)<br>5. HIFU시술 전 3개월 이내에 자궁선근증에 대한 다른 치료를 받지 않은 경우<br>*☞ 호르몬 치료제의 경우 3개월 이내라 하더라도 마지막 투여 후 해당 치료제 반감기의 5배의 기간이 경과하면 등록이 가능함.*<br>6. 임상시험에 자발적으로 동의하고 시험계획서를 준수할 의지가 있는 경우<br>7. 임신가능성이 있는 경우 의학적으로 허용된 피임법을 임상시험 기간 동안 사용하는 것에 동의하는 경우<br>** 의학적으로 허용된 피임법: 콘돔, 또는 자궁 내 피임장치의 설치 등 호르몬 조절을 통한 피임법을 제외한 물리적 피임 장치*
제외기준	다음의 제외기준에 하나라도 해당되는 경우 본 임상시험에 등록될 수 없다.<br>1. 다른 악성 종양, 자궁내막증, 난소 종양, 급성 골반 질환 등 기타 골반질환이 있는 경우<br>2. 임상적으로 육종(Sarcoma)이 의심되는 경우<br>3. 임상적으로 자궁에 전체적으로 자궁선근종이 퍼져있는 Diffuse adenomyosis로 판단되는 경우

	4. 임신반응 양성이거나 임신을 희망하는 경우<br>5. 심각한 전신 질환이 있는 경우<br>6. 헤마토크리트 25% 미만인 경우<br>7. 초음파 빛이 지나는 부위에 광범위한 복부 반흔(scar)이 있는 경우<br>☞ *단, 초음파 빛이 지나는 부위에 광범위한 복부 반흔이 있더라도 스카패치를 적용하여 HIFU 치료를 시행 할 수 있다고 연구자가 판단하는 경우 본 제외기준으로 임상시험에서 제외되지 않는다.*<br>8. 고강도 집속 초음파가 지나가는 통로에 반흔, 외과용 클립이 있는 경우<br>9. 편안한 자세로 누울 수 없는 경우<br>10. MRI 금기(폐소 공포증이 있는 경우 포함)<br>11. MRI 조영제 금기<br>12. 초음파 조영제 금기<br>13. GFR(Glomerular filtration rate, 사구체 여과율)이 30ml/min 이하인 경우<br>14. 의사소통이 곤란한 경우<br>15. 최근 1개월 이내에 타 임상시험에 참여한 경험이 있는 환자<br>16. 기타, 연구자의 판단에 따라 본 임상시험 참여가 적합하지 않다고 판단되는 경우<br>* 구체적 사유는 증례기록서에 기록
유효성<br>평가변수	<일차 유효성 평가변수><br>■ 월경통 개선율(%) - 시술 후 3개월<br><이차 유효성 평가변수><br>■ 월경통 개선율(%) - 시술 후 1개월<br>■ 월경통 지표(Dysmenorrhea Score)<br>■ 월경과다 지표(Menorrhagia Score)<br>■ 삶의 질 측정<br>- SF36v2<br>- UFS-QoL<br>- SSS<br>■ 자궁 크기(cm^3)<br>■ 시술 만족도
안전성<br>평가변수	임상시험 기간 중 피험자에게 발생한 모든 이상반응
유효성<br>평가기준	<일차 유효성 평가변수><br>■ 월경통 개선율(%)<br>• 월경통 개선의 정의<br>월경통 개선 평가지표 상 월경통 증상 완화의 정도가 Minor, Partial, Complete에 해당하는 경우<br><br><u>월경통 개선 평가지표</u>

	① Complete relief (완전한 완화)<br>② Partial relief (부분적 완화)<br>③ Minor relief (적은 완화)<br>④ Ineffective (효과 없음)<br>⑤ Exacerbated pain (통증 악화)<br><br>• 월경통 개선율의 정의<br>시술 후 3개월 시점에 위 월경통 개선 정의에 해당하는 피험자의 비율<br><br><이차 유효성 평가변수><br>■ 월경통 개선율(%) - 시술 후 1개월<br>시술 후 1개월 시점의 월경통 개선율(%)을 일차유효성 평가변수와 동일한 방법으로 평가한다.<br><br>■ 월경통 지표(Dysmenorrhea Score)<br>시술 전 및 시술 후 1, 3개월 시점에 월경통 증상을 아래와 같이 5 point scale로 피험자가 직접 평가한다.<br><br>Dysmenorrhea Score<br>1 = Not at all (증상이 없음)<br>2 = A little bit (증상이 조금 있음)<br>3 = Somewhat (증상이 약간 있음)<br>4 = A great deal (증상이 상당히 있음)<br>5 = A very great deal (증상이 아주 많이 있음)<br><br>■ 월경과다 지표(Menorrhagia Score)<br>시술 전 및 시술 후 1, 3개월 시점에 월경과다 증상을 아래와 같이 5 point scale로 피험자가 직접 평가한다.<br><br>Menorrhagia Score<br>1 = Not at all (증상이 없음)<br>2 = A little bit (증상이 조금 있음)<br>3 = Somewhat (증상이 약간 있음)<br>4 = A great deal (증상이 상당히 있음)<br>5 = A very great deal (증상이 아주 많이 있음)<br><br>■ 삶의 질 측정<br>피험자가 직접 작성하는 설문지를 통해 시술 전과 시술 후 1개월, 3개월 시점의 변화를 비교 평가한다.<br><br>▪ SF36-v2<br>전반적인 삶의 질을 평가하기 위한 설문으로 신체적 기능 영역 (physical functioning), 신체적 역할제한 영역 (role limitation-physical), 통증 영역 (bodily pain), 일반건강 영역 (general health), 활력 영역 (vitality), 사회적

	기능 영역 (social functioning), 감정적 역할제한 영역 (role limitation-emotional), 정신건강 영역 (mental health), 그 외 건강상태 변화 문항 등 총 8개 영역 36문항으로 구성되어 있다.<br>▪ UFS-QOL<br>자궁근종 증상과 관련한 삶의 질을 평가하기 위한 설문으로 8문항의 증상 심각도 점수(Symptom Severity Score)와 29문항의 삶의 질에 관한 설문으로 구성되어 있다.<br>▪ SSS(Symptom Severity Score)<br>UFS-QOL 설문에 포함된 내용이나 자궁근종으로 인한 증상의 중증도를 평가하는 것으로 별도로 평가하기로 한다.<br>■ 자궁 크기(cm^3)<br>시술 전에 대해 시술 직후, 시술 후 1개월 및 3개월의 전체 자궁 크기를 비교한다.<br>■ 시술 만족도<br>시술 중 피험자가 느낀 통증 및 시술 만족에 따른 재사용 여부를 5 point scale로 설문지를 통해 평가한다.
안전성 평가기준	본문 15. 항에 명시된 이상반응의 정의 및 평가기준에 따라 평가함
관찰항목	10.5. <임상시험진행일정표> 참고
예상되는 이상반응	• 통증<br>• 오심<br>• 구토<br>• 복부 팽만감(Abdominal tenderness)<br>• 부종<br>• 복부 경련(Abdominal cramping)<br>• 1~3도 화상<br>• 내부조직 열손상<br>• 다리와 엉덩이 통증<br>• 치료 전 이상의 질출혈<br>• 좌골신경손상<br>• 복부 및 골반 장기 손상(방광, 자궁, 장 등)<br>• 약물에 반응하지 않는 통증<br>• 비뇨기계 감염<br>• 배뇨장애<br>• 감염으로 인한 발열<br>• 조영제 사용에 대한 과민반응(어지러움, 오심, 구토, 가려움증, 두드러기, 작열감, 혈압강하, 심박동이상, 호흡곤란, 신장질환, 급성신부전 등)<br>• MR 검사 상 복부 근육 내 충혈성 변화<br>(복부 반흔이 있어 스카패치를 적용 후 시술한 경우에 한함.)

<임상시험진행일정표>

	스크리닝	시술일	추적관찰기간	
방문일	방문1	방문2*	방문3	방문4
경과일수	Day -30~0일	Day0	1개월	3개월
Visit window	-	-	± 2주	± 2주
관찰형태	내원	Day surgery 입원	내원	내원
동의서 취득	√			
선정/제외기준	√			
인구학적조사	√			
활력징후	√	√		
신체검진	√			
병력조사	√			
심전도검사	√			
실험실검사	√		√	
FSH	√		√	
임신검사	√			√
MRI	√	√	√	√
U/S	√	√	√	√
월경통 개선 지표			√	√
월경통 지표	√		√	√
월경과다 지표				
SF36-v2	√		√	√
UFS-QoL(SSS포함)	√		√	√
US-guided HIFU 시술		√		
피험자 시술 만족도		√		
이상반응/중대한 이상반응		√	√	√
병용약물	√	√	√	√

< 목 차 >

임상시험계획서 요약 ···· 3
용 어 ···· 12
1. 임상시험의 명칭 ···· 13
2. 임상시험 실시기관의 명칭 및 소재지 ···· 13
3. 임상시험의 책임자, 담당자 및 공동연구자의 성명 및 직명 ···· 13
4. 임상시험용 의료기기를 관리하는 자의 성명 및 직명 ···· 13
5. 임상시험 의뢰자의 성명 및 주소 ···· 14
6. 임상시험 수탁기관 ···· 14
7. 배경 ···· 15
7.1. 배경 ···· 15
8. 임상시험용 의료기기의 사용목적 ···· 16
8.1. 임상시험 기기의 구성 ···· 16
8.2. 작용원리 및 사용목적 ···· 16
9. 임상시험의 목적 ···· 16
10. 예상 연구기간 ···· 17
11. 임상시험방법 및 절차 ···· 17
11.1. 연구설계 ···· 17
11.2. 대상질환 및 적응증 ···· 17
11.3. 선정기준 및 제외기준 ···· 17
11.3.1. 선정기준 ···· 17
11.3.2. 제외기준 ···· 18
11.4. 표본수 수 산정 및 근거 ···· 18
11.5. 관찰항목, 임상검사항목 및 관찰검사방법 ···· 20
11.5.1. 피험자 번호 ···· 22
11.6. 임상시험방법 ···· 22
11.6.1. 임상시험용 의료기기 사용방법 ···· 22
11.6.2. 임상시험 절차 ···· 24
11.6.3. 병용요법 ···· 27
11.6.4. 연구비 ···· 27
12. 연구의 평가 ···· 28
12.1. 유효성 평가 ···· 28
12.1.1. 일차 유효성 평가변수 ···· 28
12.1.2. 이차 유효성 평가변수 ···· 28
12.2. 평가기준 및 평가방법 ···· 28

12.3. 안전성평가 ··· 30
12.3.1. 안전성 평가변수 ··· 30
12.3.2. 평가기준 및 평가방법 ··· 30
13. 자료의 수집 및 통계분석 ··· 30
13.1. 일반적 고려사항 ··· 30
13.2. 평가분석군의 정의 ··· 30
13.2.1. 유효성 평가분석군 ··· 30
13.2.2. 안전성 평가분석군 ··· 31
13.2.3. 누락자료(결측치)의 처리 ··· 31
13.3. 통계분석방법 ··· 31
13.3.1. 일반적 사항에 대한 분석 ··· 31
13.3.2. 일차 유효성 분석 ··· 31
13.3.3. 이차 유효성 분석 ··· 31
13.3.4. 안전성 분석 ··· 32
14. 임상시험 중지 및 탈락기준 ··· 32
14.1. 중지기준 ··· 33
14.2. 탈락기준 ··· 33
14.3. 중지의 처리 ··· 33
14.4. 탈락의 처리 ··· 33
15. 부작용을 포함한 안전성의 평가기준, 평가방법 및 보고방법 ··· 33
15.1. 이상반응의 정의 ··· 33
15.2. 중대한 이상반응/이상의료기기 반응의 정의 ··· 34
15.3. 이상반응의 평가 ··· 34
15.3.1. 중증도평가 ··· 34
15.3.2. 임상시험용 의료기기와의 인과관계 평가 ··· 34
15.4. 이상반응의 평가기준 ··· 35
15.5. 예상되는 이상반응 및 주의사항 ··· 35
15.5.1. 예상되는 이상반응 ··· 35
15.5.2. 사용 시 주의사항 ··· 35
15.6. 이상반응 보고방법 ··· 36
15.6.1. 이상반응교육 ··· 36
15.6.2. 예상되는 이상반응의 기록 ··· 36
15.6.3. 중대한 이상반응/이상의료기기반응 보고 ··· 37
16. 피험자 안전보호에 관한 대책 ··· 38
16.1. 의료기기 임상시험 관리기준(KGCP)과 헬싱키선언 ··· 38
16.2. 임상연구윤리위원회(IRB) ··· 38

16.3. 피험자 동의서 ··· 38
16.4. 피해자 보상에 관한 규약 ··· 39
16.5. 피험자의 안전보호에 관한 대책 ··· 39
16.6. 임상시험 후 피험자의 진료 및 치료기준 ··· 39
16.7. 임상시험실시기관 ··· 39
16.8. 시험자 ··· 39
16.9. 의뢰자 ··· 40
17. 기타 임상시험을 안전하고 과학적으로 실시하기 위하여 필요한 사항 ··· 40
17.1. 비밀보장 ··· 40
17.1.1. 데이터 ··· 40
17.1.2. 피험자의 익명성 ··· 40
17.2. 연구계획서의 준수 및 연구계획서 변경 ··· 40
17.3. 임상시험 모니터링 ··· 41
17.4. 시험결과의 기록 및 이용 ··· 42
17.4.1. 증례기록서와 근거문서 ··· 42
17.4.2. 임상연구자료의 보관 ··· 42
17.4.3. 시험결과의 이용 ··· 43
17.5. 의뢰자와 임상시험기관 장과의 계약서 ··· 43
17.6. 시험책임자의 이력사항 ··· 43
17.7. 임상시험용 의료기기의 사용 및 관리 ··· 43
17.8. 임상시험용 의료기기의 공급과 취급 ··· 43
18. 참고문헌 ··· 44

< 용 어 >

① Ablation: 소작
② Acoustic Power: 음향파워
③ ADE(AdverseDeviceEffect):이상의료기기반응
④ AE(Adverse Event): 이상반응
⑤ CRA(Clinical Research Associate): 임상시험모니터요원
⑥ DVT(Deep Vein Thrombosis): 심부정맥혈전증
⑦ FAS(Full Analysis Set): 모든 분석군
⑧ FSH(Follicle-Stimulating Hormone): 난포자극호르몬
⑨ HIFU(High Intensity Focused Ultrasound): 고강도집속초음파
⑩ IRB(Institutional Review Board): 임상연구윤리위원회
⑪ KGCP(Korea Good Clinical Practice): 임상시험관리기준
⑫ MRgHIFU(Magnetic Resonance Imaging-Guided High Intensity Focused Ultrasound): 자기공명영상 유도 하 고강도집속초음파
⑬ MRI(Magnetic Resonance Imaging): 자기공명영상
⑭ PP(Per-Protocol): 계획서순응분석군
⑮ SAE(SeriousAdverseEvent):중대한 이상반응
⑯ T1 weighted image: T1 강조영상
⑰ T2 weighted image: T2 강조영상
⑱ USgHIFU(Ultrasound-Guided High Intensity Focused Ultrasound): 초음파영상 유도 하 고강도집속초음파
⑳ NRS(Numeric Rating Scale): 통증검사

1. 임상시험의 명칭

자궁선근증 환자를 대상으로 'ALPIUS 900'(US-guided HIFU System, 초음파 유도 하 고강도 집속형 초음파 수술기)의 안전성과 유효성을 평가하기 위한 전향적, 다기관, 단일군 확증 임상시험

2. 임상시험 실시기관의 명칭 및 소재지

- 서울대학교병원
  (03080) 서울특별시 종로구 대학로 101(연건동 28), 대표전화: 02-2072-2114
- 건양대학교병원
  (35365) 대전광역시 서구 관저동로 158, 대표전화: 1577-3330

3. 임상시험의 책임자, 담당자 및 공동연구자의 성명 및 직명

- 서울대학교병원

시험책임자:	이재영 교수	영상의학과
공동연구자:	이마리아 교수	산부인과
공동연구자:	전명재 교수	산부인과
공동연구자:	김기동 교수	산부인과
공동연구자:	김훈 교수	산부인과
공동연구자:	이창순 교수	마취통증의학과
시험담당자:	윤혜신 간호사	영상의학과
연구간호사:	강수연 간호사	영상의학과
의료기기 담당자:	박동혁 방사선사	영상의학과

- 건양대학교병원

시험책임자:	김철중 교수	산부인과
공동연구자:	김태현 교수	산부인과
공동연구자:	이성기 교수	산부인과
공동연구자:	허성은 교수	산부인과
연구간호사:	최다미 간호사	산부인과

4. 임상시험용 의료기기를 관리하는 자의 성명 및 직명

- 서울대학교병원 / 박동혁 영상의학과 방사선사
- 건양대학교병원 / 최다미 연구코디네이터

5. 임상시험 의뢰자의 성명 및 주소

- 알피니언메디칼시스템(주), 대표이사: 최영춘
  서울특별시 구로구 구로디지털로 26길 72, 베르디타워 1층, 6층 (02-3282-0903)

6. 임상시험 수탁기관

(주)사이넥스, 대표이사 김 영
(06223) 서울특별시 강남구 논현로 430 아세아타워 10층
모니터 요원: 박가영 CRA (02-6202-3376)

7. 배경

7.1. 배경

자궁선근증(Adeomyosis)은 자궁내에서 발생하는 양성 질환으로 흔한 부인과 질환 중 하나이며, 인종에 따라 1~70%의 광범위한 유병율을 보이며, 보통 생식 연령대 여성의 20~30%에서 보고되고 있다.[1] 자궁선근증의 발생 원인은 정확히 밝혀진 바는 없으나, 자궁내막 조직이 자궁근층으로 침투하여 생기는 것으로 보이며,[2] 이로 인해 자궁의 크기가 임신 시 자궁이 커지는 것과 유사해지는 증상을 나타낸다. 자궁선근증의 대표적 증상은 월경과다와 심한 월경통을 들 수 있으며, 이 외에 골반 통증과 빈뇨 등의 증상이 발생할 수 있다[1]. 자궁선근증 환자들은 이와 같은 증상들이 매우 심하여, 자궁선근증의 치료 목표는 월경통 완화를 포함한 증상 조절에 있다.

자궁선근증은 증상의 정도에 따라 여러 방법으로 치료가 가능하나, 가장 확실한 방법은 자궁절제술(Hysterectomy)로 알려져 있다. 그러나, 자궁절제술(Hysterectomy)은 향후 임신을 원하는 여성에게는 적절하지 않고, 출혈, 수혈 및 수술 수 장기 유착과 전신마취에 따른 합병증 발생 위험 등의 단점이 있다. 또한, 자궁 보존이 가능한 자궁근종절제술(Myomectomy)을 통해 치료할 수 있으나 역시 수술로 인한 합병증과 임신이나 분만 중 자궁파열 등의 위험이 있을 수 있는 단점이 있다. 이 외에 환자의 생활 패턴, 비용, 침습 정도 등을 고려하여 자궁동맥 색전술(Uterine Artery Embolization), 호르몬치료 등을 적용할 수 있다. 자궁동맥 색전술은 자궁에 혈액을 공급하는 자궁동맥에 색전을 유도하여 혈액흐름을 차단하여 선근종세포를 사멸시키는 방법으로, 전신마취가 필요 없고 출혈의 가능성이 적어 합병증 발생이 드문 장점이 있으나, 색전 후 괴사 및 감염으로 자궁적출술이 필요하거나 폐경이 되는 등 부작용 발생의 위험이 있다. 또한, 호르몬치료법은 선근종의 크기와 관련된 에스트로겐 생성을 억제하여 선근종의 크기를 줄어들게 하는 생식샘자극호르몬을 사용하는 치료법으로 비침습적인 방법이나 단기간 동안 증상을 완화시키거나 수술 전 근종의 크기를 줄이기 위한 목적으로 사용되어 궁극적인 치료방법은 될 수 없다.

이러한 이유로 침습적인 시술과 약물요법 등의 단점을 보완할 수 있는 비침습적 시술의 하나인 고강도 집속 초음파(HIFU) 시스템에 대해 많은 연구가 진행 되고 있다. HIFU기술의 원리는 트랜스듀서를 통해 제한된 국소영역에 집속시켜 조사된 고강도초음파 에너지를 초점 영역에서 열에너지로 변환하고 초점 영역의 온도는 55℃ 이상으로 급격히 상승하면서 이로 인해 조직이 열 소작되는 것이다. 그러나 HIFU 초점 밖의 영역에서는 온도 상승이 거의 없어 정상 조직의 피해 없이 환부를 치료할 수 있는 기술이다.[11]

고강도 초음파를 집속시키기 위한 치료부위의 선택방법으로는 MRI영상을 사용하는 MR guided HIFU와 초음파영상을 사용하는 US guided HIFU로 나뉘어진다.

MRgHIFU는 MRI 시스템에 고강도 초음파 집속 장치를 연결하여 MR 영상을 통해 소작 할 조직의 위치를 선택하고 선택한 조직을 HIFU를 이용하여 치료하며 MRI 영상을 통해 치료과정 및 치료효과를 관찰하는 시스템이며 선행연구들에서 좋은 치료 효과를 얻은 것을 알 수 있었다.[5-7] 그러나 MR시스템에 고강도 집속 초음파 장치를 연결해서 사용하므로 치료가 진행되는 동안 영상용 MRI 진료를 시행할 수 없는 점과 MRI실이 따로 필요하다는 점, 그리고 기본적으로 장비와 치료비가 고가이며 MR 영상을 이용하므로, 실시간 영상 치료가 불가능하여 환자 상태 움직임에 따른 치료가 어렵고 치료시간이 많이 걸릴 뿐만 아니라 대형병원 중심으로 수요가 형성

되기 때문에 시장이 제한적이라는 한계점이 있다.

반면에 USgHIFU는 초음파 영상 장치를 통해 획득한 영상을 이용하여 소작조직의 위치를 선택하고 HIFU를 이용하여 치료하며, 초음파 영상을 통해 치료과정 및 치료효과를 관찰하는 시스템으로써 MRgHIFU 및 타 치료장비에 비해 저가이며 치료비 역시 상대적으로 저렴할 뿐만 아니라 실시간 영상을 통한 치료가 가능하고 치료시간이 훨씬 짧다는 장점과 MRI실 같은 특수한 공간이 필요 없을 뿐만 아니라 치료시간 동안 MRI는 다른 환자들이 사용 할 수 장점이 있다.

HIFU 시술은 여러 연구를 통해 자궁근종(Uterine Fibroid) 증상 완화에 효능이 있음이 증명된 바 있으며, 최근 자궁선근증(Adenomyosis) 치료 효과를 본 몇몇 연구가 진행되었다. 한 해외제조사의 MRgHIFU 제품은 2008년 일본에서 HIFU의 치료 효능을 확인하기 위한 초기 임상을 진행하여 HIFU 치료가 자궁선근증 환자의 증상 완화에 도움이 된다는 연구 결과를 발표하였고[4], 2010년에는 자궁선근증에 대한 CE marking을 득하였다. 이 외에도 여러 연구[1-3]에서 HIFU가 자궁선근증 치료를 위한 대체 치료로서 안전하고 유효함을 확인하였다.

위와 같은 배경 하에 알피니언메디칼시스템㈜은 MR-guided HIFU와 같은 고강도집속 초음파수술기의 성능은 유지하면서 적은부피를 차지하고 이동성이 우수한 US-guided HIFU 'ALPIUS 900'을 개발하였고, 자궁근종에 대한 유효성 및 안전성을 입증하여 국내 품목 허가를 득하였다(제허 14-3227호). 본 임상시험은 'ALPIUS 900'의 기 허가된 자궁근종에서 적응증을 확대하여 자궁선근증에서의 치료 유효성 및 안전성을 입증하고자 계획되었다(식약처 품목허가용).

8. 임상시험용 의료기기의 사용목적

- 제품명: ALPIUS 900(제허14-3227호)
- 품목분류: 고강도집속형초음파수술기, High intensity focused Ultrasonic surgical unit (3등급, A35100.02), 고강도로 집적된 초음파를 이용하여 암 등을 치료하는 기구
- 기 허가사항: 초음파영상 유도에 따라 고강도집속초음파를 이용하여 3cm 이상 12cm 이하의 자궁 근종을 비침습적으로 치료

8.1. 임상시험 기기의 구성

<별첨 참고> ALPIUS 900 User Manual

8.2. 작용원리 및 사용목적

ALPIUS 900은 초음파영상 유도 하에 고강도 집속 초음파를 조사하여 자궁선근종을 소작하는데 사용된다.

9. 임상시험의 목적

본 임상시험은 자궁근종 적응증에 대해 제조품목허가 된 알피니언메디칼시스템(주)의 초음파 유도 하 고강도 집속형 초음파 수술기인 'ALPIUS 900(US-guided HIFU System)'의 "증상이 있는

자궁선근증(Adenomyosis)"에 대한 비침습적 자궁선근증 소작술(ablation)의 유효성과 안전성을 확인하기 위함이다. (식약처 허가용)

10. 예상 연구기간

식품의약품안전처로부터 임상시험계획승인을 득한 후 피험자등록기간 약 25개월, 추적관찰기간 3개월을 포함하여 약 28개월이 소요될 것으로 예상하며, 임상시험이 종료 된 후에도 데이터처리, 통계분석, 결과보고서 작성 및 IRB승인을 위해 약 3개월이 추가로 소요될 것으로 예상한다.

11. 임상시험방법 및 절차

11.1. 연구설계

본 임상시험은 증상이 있는 자궁선근증 환자를 대상으로 초음파영상 유도 하 고강도 집속형 초음파 수술기인 'ALPIUS 900'의 안전성과 유효성을 평가하기 위해 계획되었으며, 전향적, 다기관, 단일군 임상시험으로 국내 2개 기관에서 총 80명을 대상으로 실시될 예정이다.

11.2. 대상질환 및 적응증

자궁선근증 소작술이 필요한 환자

11.3. 선정기준 및 제외기준

11.3.1. 선정기준

다음의 선정기준을 모두 만족해야 본 임상시험에 등록될 수 있다.

1) 만 20세 이상 성인 여성
2) 폐경 이전 또는 폐경기 전후인 경우 (FSH<40mIU/ml)
3) MR 또는 US 영상을 통해 임상적으로 자궁선근증을 진단받은 경우
4) 월경통 지표 상 통증 점수가 4점 이상인 경우
   <u>* 월경통 지표(Dysmenorrhea Score)</u>
   1 = Not at all (증상이 없음)
   2 = A little bit (증상이 조금 있음)
   3 = Somewhat (증상이 약간 있음)
   4 = A great deal (증상이 상당히 있음)
   5 = A very great deal (증상이 아주 많이 있음)
5) HIFU시술 전 3개월 이내에 자궁선근증에 대한 다른 치료를 받지 않은 경우
   ☞ 호르몬 치료제의 경우 3개월 이내라 하더라도 마지막 투여 후 해당 치료제 반감기의 5배의 기간이 경과하면 등록이 가능함.

6) 임상시험에 자발적으로 동의하고 시험계획서를 준수할 의지가 있는 경우
7) 임신가능성이 있는 경우 의학적으로 허용된 피임법을 임상시험 기간 동안 사용하는 것에 동의하는 자
   ** 의학적으로 허용된 피임법: 콘돔, 또는 자궁 내 피임장치의 설치 등 호르몬 조절을 통한 피임법을 제외한 물리적 피임 장치*

11.3.2. 제외기준

다음의 제외기준에 하나라도 해당되는 경우 본 임상시험에 등록될 수 없다.

1) 다른 악성 종양, 자궁내막증, 난소 종양, 급성 골반 질환 등 기타 골반질환이 있는 경우
2) 임상적으로 육종(Sarcoma)이 의심되는 경우
3) 임상적으로 자궁에 전체적으로 자궁선근종이 퍼져있는 Diffuse adenomyosis로 판단되는 경우
4) 임신반응 양성이거나 임신을 희망하는 경우
5) 심각한 전신 질환이 있는 경우
6) 헤마토크리트 25% 미만인 경우
7) 초음파 빛이 지나는 부위에 광범위한 복부 반흔(scar)이 있는 경우
   ☞ 단, 초음파 빛이 지나는 부위에 광범위한 복부 반흔이 있더라도 스카패치를 적용하여 HIFU 치료를 시행 할 수 있다고 연구자가 판단하는 경우 본 제외기준으로 임상시험에서 제외되지 않는다.
8) 고강도 집속 초음파가 지나가는 통로에 반흔, 외과용 클립이 있는 경우
9) 편안한 자세로 누울 수 없는 경우
10) MRI 금기(폐소 공포증이 있는 경우 포함)
11) MRI 조영제 금기
12) 초음파 조영제 금기
13) GFR(Glomerular filtration rate, 사구체 여과율)이 30ml/min 이하인 경우
14) 의사소통이 곤란한 경우
15) 최근 1개월 이내에 타 임상시험에 참여한 경험이 있는 환자
16) 기타, 연구자의 판단에 따라 본 임상시험 참여가 적합하지 않다고 판단되는 경우
    * 구체적 사유는 증례기록서에 기록

11.4. 표본수 산정 및 근거

자궁선근증 치료를 위한 US-guided HIFU의 유효성을 평가한 Zhou et al.(2011)[3]의 연구 결과에 의하면, 전체 69명 피험자 중에서 치료 후 62명의 월경통이 완화되어 약 90%의 개선 성공률을 보였다. 본 임상에서는 이 결과를 근거로 reference 성공률을 90%로 가정하고, 'ALPIUS 기기의 성공률이 reference 성공률과 비교하였을 때 비열등할 것이다'는 가설을 만족하기 위한 피험자 수를 산출하였다. 또한, 자궁선근증을 대상으로 시행된 US-guided HIFU 시술의 유효성을 평가

한 최근 연구결과인 Zhang et el.(2014)[5] 에서는 Menorrhagia 와 Dysmenorrhea 의 완화정도를 평가한 결과 79.5%~87.9%의 성공률을 보였고 Wang et al.(2009)[12]에서는 66.7%의 개선 성공률을 제시하였으므로, 본 연구에서는 이 중 최소값인 66.7%보다 큰 77.7%를 '비열등성을 만족하는 최소 성공률'로 가정하였다 (즉, 비열등성 한계값= 77.7%-90% = -12.3%).
본 연구의 통계적 가설은 다음과 같다.

$$H_0 : P_A - P_0 \leq -\delta \ vs \ H_1 : P_A - P_0 > -\delta$$

- P_A: 본 연구기기의 기대되는 성공률
- P_0: Reference 성공률
- δ (>0): 비열등성 한계값

위 가설검정을 위한 표본수 산출을 위해 유의수준 단측 0.025, 검정력 80%, reference 성공률과 본 연구기기의 기대 성공률은 90%, 비열등성 한계 12.3%로 설정하고, 실제 성공률과 reference 성공률의 차이값 D=0을 가정하였을 때, 비열등성 가설을 만족하는 피험자수는 PASS13 (NCSS statistics software, Kaysville, UT)을 통해 아래와 같이 산출하였다.

One Proportion Non-inferiority

Numeric Results for testing H0: P = P0 versus H1: P > P0
Higher Proportions are Better
Test Statistic: Z Test using S(P0)

Power	N	Non-Inf. Proportion (P0)	Actual Proportion (P1)	Target Alpha	Actual Alpha	Beta	Reject H0 If Z ≥ This
0.8201	72	0.7770	0.9000	0.0250	0.0262	0.1799	1.9600

그 결과, 총 72명의 피험자가 산출되었으며, 중도 탈락율 10%를 고려하여 80명의 피험자를 등록하고자 한다.

<피험자수 산출 참고문헌>

1. X Zhang et al. Effective ablation therapy of adenomyosis with ultrasound-guided high-intensity focused ultrasound. International Journal of Gynecology and Obstetrics. 2014;124:207-211.
2. M Zhou et al. Ultrasound-guided high-intensity focused ultrasound ablation for adenomyosis: the clinical experience of a single center. Fertility and Sterility. 2011;95(3):900-905.
3. W Wang et al. Safety and efficacy of high intensity focused ultrasound ablation therapy for adenomyosis. Academic Radiology. 2009;16(11):1416-1423.

11.5. 관찰항목, 임상검사항목 및 관찰검사방법

<임상시험진행일정표>

	스크리닝	시술일	추적관찰기간	
방문일	방문1	방문2*	방문3	방문4
경과일수	Day -30~0일	Day0	1개월	3개월
Visit window	-	-	± 2주	± 2주
관찰형태	내원	Day surgery 입원	내원	내원
동의서 취득	√			
선정/제외기준	√			
인구학적조사[1]	√			
활력징후[2]	√	√		
신체검진[3]	√			
병력조사[4]	√			
심전도검사[5]	√			
실험실검사[6]	√		√	
FSH	√		√	
임신검사[7]	√			√
MRI[8]	√	√	√	√
U/S[9]	√	√	√	√
월경통 개선 지표[10]			√	√
월경통 지표[11]	√		√	√
월경과다 지표[12]	√		√	√
SF36-v2[13]	√		√	√
UFS-QoL(SSS포함)[14]	√		√	√
US-guided HIFU 시술		√		
피험자 시술 만족도[15]		√		
이상반응/중대한 이상반응[16]		√	√	√
병용약물[17]	√	√	√	√

1. 인구학적 조사

피험자의 기초정보인 생년월일, 성별, 신장, 체중

2. 활력징후

체온, 혈압(수축기/이완기), 맥박, 호흡수

3. 신체검진

외관, 피부, 두/경부, 흉부/폐, 심장, 복부, 비뇨/생식계, 사지, 근골격계, 신경계, 림프절, 기타 신체기관

4. 병력조사

과거 1년 이내 병력 조사

5. 심전도검사

심전도는 시술을 위한 스크리닝 검사로 시행된다. 심전도 상 이상여부가 발견되는 경우 본 임상시험에 등록될 수 없다.

6 실험실 검사

실험실 검사는 전신적인 건강상태를 평가하기 위해 모든 피험자에게 실시하며, 검사항목은 다음과 같다.

- 혈액학적검사: Hemoglobin, Hematocrit, RBC count, WBC count with differential, platelet count
- 혈액화학적검사: Alkaline phosphatase, BUN, Creatinine, SGPT(ALT), SGOT(AST), Albumin, Total protein, Total bilirubin, Uric acid, Glucose, Cholesterol, LDH, Na, K, Cl, Ca

7 임신검사

문진을 통해 불임수술 및 폐경이 확인된 피험자를 제외한 가임기 여성의 경우 Urine HCG 검사를 통해 임신여부를 확인한다(단, 스크리닝 방문 시 임신검사는 시술 전 3일 이내에 이루어져야 한다.).

8 MRI

스크리닝 방문 시 자궁의 크기, 자궁선근종의 위치, 조영제에 대한 반응 및 빔 통과경로 등을 확인하기 위해 조영증강 전 후의 T1-과 T2-weighted 이미지를 3개 직교면에 대해 촬영한다. 시술 직후 및 추적관찰 기간에는 계획 대비 치료된 용적을 확인하고 자궁의 크기 변화를 확인하기 위해 조영증강 Fat-saturated T1-weighted와 T2-weighted 이미지를 획득한다.(총 4회)

모델명	품목명	허가번호	수입원
Ingenia 3.0T	초전도자석전신용<br>자기공명전산화단층장치	수허 11-1257 호	㈜필립스코리아

9 U/S

스크리닝 방문 시 자궁의 크기와 자궁선근종의 위치를 확인하기 위하여 초음파 검사를 시행하고, 치료 시에는 ALPIUS 900에 내장된 초음파 트랜스듀서의 영상을 통하여 실제 치료를 모니터링하면서 진행한다. 시술 직후에 초음파 검사를 통해 시술 후 자궁 및 자궁선근종의 상태를 확인하고, 추적관찰기간 중 1개월, 3개월 시점에 자궁의 크기 변화를 초음파 영상으로 확인한다.(총 4회) 초음파 검사 시 조영제가 사용될 수 있으며, 상세한 검사 방법은 연구자의 판단 및 기관의 표준절차에 따른다.

제품명	품목명	허가번호	수입원
LOGIQ E9	범용초음파영상진단장치	수허 09-180 호	지이헬스케어코리아(주)

[10] 월경통 개선지표
시술 후 1개월, 3개월 시점에 각 피험자 별로 월경통이 개선된 정도를 5 point scale로 설문지를 통해 직접 평가한다.

[11] 월경통 지표
시술 전과 시술 후 1개월, 3개월 시점에 각 피험자 별로 월경통 증상 정도를 5 point scale로 설문지를 통해 직접 평가한다.

[12] 월경과다 지표
시술 전과 시술 후 1개월, 3개월 시점에 각 피험자 별로 월경과다 증상 정도를 5 point scale로 설문지를 통해 직접 평가한다.

[13] SF36-v2
전반적인 삶의 질을 평가하는 설문지로, 신체적 기능 영역 (physical functioning), 신체적 역할제한 영역 (role limitation-physical), 통증 영역(bodily pain), 일반건강 영역 (general health), 활력 영역 (vitality), 사회적 기능 영역 (social functioning), 감정적 역할제한 영역 (role limitation-emotional), 정신건강 영역 (mental health), 그 외 건강상태 변화 문항으로 총 8개 영역 36문항으로 구성되어 있으며 피험자가 직접 작성한다.

[14] UFS-QoL(SSS포함)
자궁근종 증상과 관련한 삶의 질을 평가하기 위한 설문으로 8문항의 증상심각도 점수(Symptom Severity Score)와 29문항의 삶의 질에 관한 설문으로 구성되어 있으며 피험자가 직접 작성한다.

[15] 피험자 시술 만족도
시술 중 피험자가 느낀 통증 및 시술 만족에 따른 재사용 여부를 5 point scale로 설문지를 통해 평가한다.

[16] 이상반응/중대한 이상반응
프로토콜의 16항에 정의된 기준에 따라 평가하여 증례기록서의 이상반응/중대한 이상반응 기록지에 기록한다.

[17] 병용약물
임상연구 결과에 영향을 미칠 수 있는 약물은 금지되며, 기저질환으로 인해 이미 복용하고 있는 약물은 스크리닝 시에 모두 기록하고 이후에는 추가되는 약물에 대해서만 증례기록서에 기록한다.

* 방문2에 시술 후 연구자의 판단 및 병원의 표준 절차에 따라 피험자는 시술 당일 퇴원하거나 하루정도 입원 할 수 있다.

11.5.1. 피험자 번호

피험자 동의서에 서명하고 연구 참여에 등록된 모든 피험자에 대한 관리로그가 작성될 것이다. 이러한 관리로그는 임상시험에 등록되는 피험자에게 순차적인 피험자번호를 배정하는데 사용되며, 피험자 번호는 ‘스크리닝 번호’와 ‘등록번호’가 배정된다.

스크리닝 번호는 기관번호 한자리, 스크리닝의 'S'와 배정되는 01, 02, 03, … 피험자 순서 두 자리를 포함하여, 아래의 방법에 따라 피험자 식별코드를 부여한다.

- 임상시험실시기관 한 자리: 서울대학교병원 −1, 건양대학교병원 -2
- 스크리닝: S
- 등록된 순서에 따라 2 자리

예) 서울대학교병원에 첫 번째 스크리닝 된 대상의 식별코드 : 1 S – 0 1

등록번호는 기관번호 한 자리, 배정되는 01, 02, 03, … 피험자 순서 두 자리를 포함하여, 아래의 방법에 따라 피험자 식별코드를 부여한다.

- 임상시험실시기관 한 자리: 서울대학교병원 −1, 건양대학교병원 -2
- 등록된 순서에 따라 두 자리

예) 서울대학교병원에 첫 번째 등록된 피험자의 식별코드 : 1 – 0 1

11.6. 임상시험방법

11.6.1. 임상시험용 의료기기 사용방법

임상시험용 기기의 사용 절차는 다음과 같으며, 각 호의 자세한 사항은 제조원의 사용설명서(별첨)를 따른다.

■ 사용 전 준비사항
- 전원장치 확인 및 ON/OFF
- 이미지 트랜스듀서 연결 및 분리 확인
- 이미지 트랜스듀서 활성화 및 불활성화 확인

■ 사용방법 및 조작방법
① 기기 준비
② 피험자 준비

1. 시술 전 과정

1) 외래 진료
- 자궁선근증을 최초로 진단받은 병원의 CT, MRI, 초음파 영상이나 현재 치료받고 있는 병원의 진단 영상물 또는 기타 치료법을 치료받은 내역이나 소견서 등을 받은 피험자를 진료한다. 치료에 적합한 피험자로 확인이 되면 시술 일정을 예약한다.

2) 시술 전 피험자 준비 (시술의의 처방에 따름)
- 시술 전날 금식
- 피부상태 청결히 유지
- 피부상태에 따라 제모(기계적 화학적 제모)
- 시술의 처방에 따라 마취제(Anesthetic) 또는 진정제(Sedation) 투여

- 위치 : 앙와위 (Supine) 또는 측위 (Lateral)
- 활력징후 (혈압, 맥박, 호흡수) 측정

2. HIFU 시술과정

1) 피험자의 시술 준비상태를 확인한 후, 시술 부위를 스캔한다. 시술의는 사전에 수립된 시술계획에 따라 병변 조직을 괴사 시킨다. 시술은 통상적으로 한 번의 시술로 조직을 괴사 시킨다.

2) 시술의는 시술 중에 실시간으로 병변의 괴사 여부를 초음파를 통해 확인하고, 필요한 경우 피험자를 고정한다.

3. 시술 후 과정

1) 치료가 종료된 피험자는 의료진의 소견에 따라 치료당일 귀가하여 사회활동을 하거나 하루 정도 입원하여 다음날 귀가한다.

2) 외래방문 및 추적검사 : 시술 1개월, 3개월 후 시술효과 추적과 괴사된 병변 조직의 용적을 측정하기 위해서 MRI검사를 한다.

4. 사용 시 주의사항

<별첨 참고> ALPIUS 900 User Manual

11.6.2. 임상시험 절차

1) 스크리닝 <방문1>

MR이나 US 영상을 통해 자궁선근증 진단을 받은 환자는 부인과나 중재방사선과로부터 본 임상시험에 의뢰 되고, 본 연구와 관련된 충분한 설명을 들은 후 자발적으로 서면동의서에 서명하고 선정/제외기준을 모두 만족하면 본 임상시험에 등록될 것이다.

스크리닝 방문 시에는 선정/제외기준 이외에 인구학적 조사, 활력징후, 신체검진, 과거병력, 혈액검사 및 임신검사, 심전도, MRI, 초음파 검사 등과 설문지를 통해 월경 통증과 월경과다 정도를 평가하게 되며, 연구자와의 의사소통 능력 및 임상시험 준수 의지도 선별 기간 중에 평가될 것이다.

■ 시술 전 MRI 검사

시술 전에 조영제 사용 전과 후의 MRI 이미지 및 T1- 과 T2-weighted 이미지를 3개의 직각면(3 orhthogonal planes)에 대하여 획득하고, MR 조영제 및 약한 안정제와 같은 약물을 투여하기 위해 정맥 카테터가 삽입 될 수 있다.

시술 전 MRI는 선정/제외를 결정하는 마지막 단계이며, 치료 후 MR 영상을 위해 병원에서 일반적으로 부인과 진료 영상을 위해 사용되는 조영제가 제품의 설명서에 따라 사용될 것이다. (병원의 표준절차에 따름)

2) 시술일 <방문2>

■ HIFU 치료 프로토콜

US-guided HIFU 시술은 시술 종료 후 회복 시간(1~2시간)을 포함하여 2~4시간 정도가 소요될 것으로 예상한다. 피험자가 움직이지 않고 있는 시간은 심부정맥혈전증(DVT, Deep Vein Thrombosis)의 위험을 줄이기 위해 약 3시간 이내로 제한되어야 한다.

■ 준비

피험자는 시술 전 3일 이내에 골반부위에 제모(제모크림 사용)를 하게 되며 면도 후에는 어떠한 크림도 사용해서는 안 된다. 전신마취를 하지는 않으나 위장관 내 가스나 음식물로 인한 부작용을 줄이기 위해 시술 전날 자정부터 금식하도록 하고(MN NPO), 시술 당일에는 방광 내에 적절한 양의 소변이 있는지 확인하고 필요한 경우 소변을 더 채우거나 일부 배뇨하여 치료에 적절한 방광의 용적을 유지하도록 한다. 임신 검사는 스크리닝 시에 시행했다 하더라도 시술 전 3일 이내 기간에 해당하지 않으면 다시 검사하도록 하고, 혈압, 맥박수, 호흡수와 같은 활력징후 역시 기록될 것이다. 치료의 기초 온도 정보로 사용하기 위해 피험자의 체온도 역시 기록될 것이다.

■ 자궁선근종 확인 및 치료

치료할 자궁선근종의 용적 비율은 연구자가 결정할 것이고, 현재 수반하는 증상(월경통, 압통, 과다출혈) 등을 기초로 한다. 치료 전 자궁의 조영제 사용 MR 영상을 기초로, 조영제가 증강(enhancing) 되는 자궁선근종만이 이 연구에서 치료 대상으로 고려될 것이다. 조영제가 증강 되지 않는 자궁선근종은 치료되어서는 안 된다.

다음의 경계부위는 치료되지 않아야 한다.

1) 자궁장막(Uterine serosa) 1.5cm 이내에 초음파를 조사해서는 안 된다.
2) 척추나 다른 골조직의 4cm 이내에는 초음파를 조사해서는 안 된다.
3) 자궁내막표면 까지는 초음파를 조사할 수 있을 것이다.

■ 피험자 위치 및 치료 계획

1) 피험자를 치료 테이블에 반듯이 눕힌다.
2) 치료용 초음파의 탐촉자가 밀착이 되도록 초음파용 젤을 피험자의 피부에 충분히 도포한다.
3) 치료용 초음파의 탐촉자를 하강시켜 피험자의 하복부와 밀착 시킨 후 치료할 부위에 대한 스캔이미지 정보를 얻는다.
4) 획득한 이미지는 XZ, YZ, XY 평면의 이미지를 표현하여 원하는 스캔 및 치료 부의를 탐색할 수 있다.
5) 치료할 부위를 그리는 프로그램(Target Draw)을 활성화하여 치료영역을 표시한다.

6) 선정한 영역을 기준으로 다음과 같은 HIFU의 조사방법을 상세하게 설정할 수 있다.
   - Focal Point Grid: 점들 간 간격(X, Y)를 설정 및 Cartesian(격자형), Cylinder(실린더)형 포컬 포인트 배치를 설정할 수 있다.
   - Acoustic Power : 음향 전력(강도)
   - PRF(Pulse Repetition Frequency) : 초당 조사 횟수
   - Duty : 조사 간 시간 간격에서 조사 유지 시간을 퍼센트 단위로 설정
   - Exposure Time : 한 점에 조사하는 시간
   - Point to Point : 포인트 간 이동 시간
   - Cooling Time : 슬라이스 이미지 간 이동 시간
   - Generate Points: 원하는 영역에 Cartesian(격자형), Cylindrical(실린더형)의 패턴으로 포컬 포인트가 자동 생성된다.
   - Add Point: 원하는 영역에 일정한 패턴을 가지지 않은 포컬 포인트를 수동으로 하나씩 생성할 수 있다.
7) 선행 단계에서 설정된 피험자의 치료계획을 바탕으로 실제 치료하기 전에 시뮬레이션을 수행하여 치료계획의 유효함을 검사한다.
8) 피험자를 치료하기 전에 HIFU 조사의 정확성을 실험하기 위해 Pre-Targeting을 실행한다. Pre-Targeting은 사용자가 조사할 위치를 임의로 설정하고 실제로 HIFU를 약하게 조사하여 조사된 위치 정보를 통해 그 정확성을 확인한다.

■ 치료절차

시술자는 사전에 수립된 시술계획에 따라 HIFU치료를 하고, 초음파 이미지와 소프트웨어를 통해 병변의 괴사여부와 치료 경과를 관찰하게 된다. 방광 내에 소변이 차올라 자궁의 위치가 변화되었을 때는 치료 위치를 조정하고 다시 시작한다. 계획한 전체 부위의 치료가 끝나면 피험자기록에 전체 치료 기록을 보관한다.

■ 치료 이후

초기에 설정한 치료 범위에 따라 치료가 완료되면 시술자는 계획된 용적과 치료 용적의 범위를 확인하기 위해 초음파와 MRI를 통해 치료 부위의 이미지를 획득하고 피부의 변화를 포함한 이상반응 여부를 관찰 한다. 피험자는 회복실에서 1~2시간 경과를 관찰 한 후 특별한 이상이 없으면 치료당일 또는 그 다음날 퇴원할 수 있으며, 퇴원 전 시술만족도에 대한 설문지를 작성한다.

3) 추적관찰기간 <방문3, 4>

■ 방문 3(치료 후 1개월 시점)

내원하여 다음과 같은 검사를 시행 한다
- 자궁 크기 측정(MRI)
- 초음파 검사
- 혈액검사 및 FSH검사
- 설문지 작성: 월경통 개선 및 월경통/월경과다 지표, 삶의 질(SF36-v2, UFS-QoL)

- 이상반응/중대한 이상반응 확인
- 병용약물 확인

■ 방문 4(치료 후 3개월 시점)

내원하여 다음과 같은 검사를 진행 한다.

- 자궁 크기 측정(MRI)
- 초음파 검사
- 임신검사
- 설문지 작성: 월경통 개선 및 월경통/월경과다 지표, 삶의 질(SF36-v2, UFS-QoL)
- 이상반응/중대한 이상반응 확인
- 병용약물 확인

11.6.3. 병용요법

자궁선근증 치료에 영향을 미칠 수 있는 약물(아래 병용 금지 약물 참고)이나 대체치료는 금지되며, 기저질환으로 인해 이미 복용하고 있는 약물은 스크리닝 시에 모두 기록하고 이후에 추가 되는 약물에 대해서만 증례기록서에 기록한다. 임상시험기간 중 위와 같은 병용금기 약물이나 대체치료를 받았다면 이들은 연구에서 탈락될 것이고 탈락사유를 기록한다.
다만, 병용금기 약물이라 하더라도 본 연구와 관련 없이 일회성으로 투여가 필요할 경우, 연구자 판단 하에 시술 결과에 영향을 미치지 않는 범위 내에서 사용이 가능하며, 관련 내용은 증례기록서 병용약물 란에 기록한다.

피험자들은 시술 전 진정(sedation)을 위해 미다졸람과 펜타닐을 투여받는다. 미다졸람과 펜타닐의 투여용량은 연구자가 피험자의 연령 및 임상상태를 고려하여 판단하나, 일반적으로 미다졸람 3mg/3ml 또는 5mg/5ml, 펜타닐 100mcg/2ml 의 용량으로 투여된다. 연구자의 판단에 따라 피험자의 상태를 고려하여 진통제, 진정제 및 국소 마취제를 기관의 표준절차에 따라 투여할 수 있다.

<u>병용 금지 약물</u>

Estrogen(including combined equine estrogen), Progesterone, Steroid(부신피질 호르몬 제제 포함), 경구용 피임제 등

11.6.4. 연구비

본 임상시험에 의뢰되기 전 피험자가 지불한 비용은 소급 적용되어 보상되지 않으며, 임상참여 이후 발생하는 비용(심전도검사, 실험실검사, 임신검사, MRI, U/S, US-HIFU시술) 등은 연구자가 부담하게 될 것이다. 또한, 본 임상시험 참여로 인해 소요되는 시간과 관련하여 매 방문(방문3과 4)시 사례비는 해당 기관 거점 지역의 경우 10만원, 해당 기관 거점 외 지역에 거주하는 경우 20만원을 계좌이체를 통해 지급되어 보상 될 것이며, 지급에 대한 내역은 피험자 사례비 지급로그를 통해 관리될 것이다. 연구피험자에 대한 보상은 임상시험에 끝까지 참여할 것을 조건으로 하지 않을 것이다.

12. 연구의 평가

12.1. 유효성 평가

12.1.1. 일차 유효성 평가변수

■ 월경통 개선율(%) - 시술 후 3개월

12.1.2. 이차 유효성 평가변수

■ 월경통 개선율(%) - 시술 후 1개월
■ 월경통 지표(Dysmenorrhea Score)
■ 월경과다 지표(Menorrhagia Score)
■ 삶의 질 측정
- SF36-v2
- UFS-QoL
- SSS
■ 자궁 크기(cm^3)
■ 시술 만족도

12.2. 평가기준 및 평가방법

<일차 유효성 평가기준>

■ 월경통 개선율(%)

- 월경통 개선의 정의

월경통 개선 평가지표 상 월경통 증상 완화의 정도가 Minor, Partial, Complete에 해당하는 경우

<u>월경통 개선 평가지표</u>
① Complete relief (완전한 완화)
② Partial relief (부분적 완화)
③ Minor relief (적은 완화)
④ Ineffective (효과 없음)
⑤ Exacerbated pain (통증 악화)

- 월경통 개선율의 정의

시술 후 3개월 시점에서 위 월경통 개선 정의에 해당하는 피험자의 비율

<이차 유효성 평가변수>

■ 월경통 개선율(%) - 시술 후 1개월

시술 후 1개월 시점의 월경통 개선율(%)을 일차유효성 평가변수와 동일한 방법으로 평가한다.

■ 월경통 지표(Dysmenorrhea Score)

시술 전 및 시술 후 1, 3개월 시점에 월경통 증상을 아래와 같이 5 point scale로 피험자가 직접 평가한다.

<u>Dysmenorrhea Score</u>
1 = Not at all (증상이 없음)
2 = A little bit (증상이 조금 있음)
3 = Somewhat (증상이 약간 있음)
4 = A great deal (증상이 상당히 있음)
5 = A very great deal (증상이 아주 많이 있음)

■ 월경과다 지표(Menorrhagia Score)

시술 전 및 시술 후 1, 3개월 시점에 월경과다 증상을 아래와 같이 5 point scale로 피험자가 직접 평가한다.

<u>Menorrhagia Score</u>
1 = Not at all (증상이 없음)
2 = A little bit (증상이 조금 있음)
3 = Somewhat (증상이 약간 있음)
4 = A great deal (증상이 상당히 있음)
5 = A very great deal (증상이 아주 많이 있음)

■ 삶의 질 측정

피험자가 직접 작성하는 설문지를 통해 시술 전과 시술 후 1개월, 3개월 시점의 변화를 비교 평가

- <u>SF36-v2:</u> 전반적인 삶의 질을 평가하기 위한 설문으로 신체적 기능 영역 (physical functioning), 신체적 역할제한 영역 (role limitation-physical), 통증 영역(bodily pain), 일반건강 영역 (general health), 활력 영역 (vitality), 사회적 기능 영역 (social functioning), 감정적 역할제한 영역 (role limitation-emotional), 정신건강 영역 (mental health), 그 외 건강상태 변화 문항 등 총 8개 영역 36문항으로 구성되어 있다
- <u>UFS-QOL:</u> 자궁근종 증상과 관련한 삶의 질을 평가하기 위한 설문으로 8문항의 증상 심각도 점수(Symptom Severity Score)와 29문항의 삶의 질에 관한 설문으로 구성되어 있다.
- SSS(Symptom Severity Score): UFS-QOL 설문에 포함된 내용이나 자궁근종으로 인한 증상의 중증도를 평가하는 것으로 별도로 평가하기로 한다.

■ 자궁 크기(cm^3)

시술 전에 대해 시술 직후, 시술 후 1개월 및 3개월의 전체 자궁 크기를 비교한다.

■ 시술 만족도

시술 중 피험자가 느낀 통증 및 시술 만족에 따른 재사용 여부를 5 point scale로 설문지를 통해 평가한다.

12.3. 안전성평가

12.3.1. 안전성 평가변수

임상시험기간 중 피험자에게 발생한 모든 이상반응

12.3.2. 평가기준 및 평가방법

본 임상시험에서 이상반응은 임상시험 시작 전에 관찰되지 않은 증상이 발생하는 모든 바람직하지 않은 의학적 소견을 이상반응으로 분류한다. '15. 부작용을 포함한 안전성의 평가기준, 평가방법 및 보고방법 ' 항에서 서술된 정의 및 기준에 따라 예측되는 부작용도 이상반응으로 분류하며, 이상반응의 정도를 경증, 중등증, 중증으로 분류하고 용어의 사용은 MedDRA의 'Preferred term' 및 'System organ class'를 기준으로 한다.

13. 자료의 수집 및 통계분석

13.1. 일반적 고려사항

본 연구에서 측정되어 기록된 모든 자료는 14.2.에서 정의되는 적절한 분석군의 정의에 따라 모든 자료가 요약 정리될 것이다. 요약 통계량은 연속형 자료의 경우 평균±표준편차로, 범주형 자료의 경우 빈도(분율)을 기본 원칙으로 하고 필요시에 보다 상세한 요약통계량을 제시할 것이다.

13.2. 평가분석군의 정의

본 임상시험에서 유효성 분석은 FAS(Full Analysis Set)를 주 분석군으로 정의하고 이를 토대로 평가를 시행 하며, 보조적 분석으로써 PP 분석군(Per-Protocol Analysis Set)에 대해서 동일한 평가를 반복해서 시행한다. 안전성 평가는 연구참여 동의 후 연구에 참여하여 임상시험용기기로 시술을 받은 모든 피험자에 대해서 실시한다.

13.2.1. 유효성 평가분석군

■ FAS 분석군(Full Analysis Set): 본 연구의 유효성 평가의 주 분석군으로, 연구 참여 동의 후 연구에 참여하여 임상시험용 기기를 시술 받고 일회 이상의 유효한 평가 자료가 있는

모든 피험자로 정의 한다.

- PP 분석군(Per Protocol Analysis Set): 연구 참여 동의 후 연구에 참여하여 임상시험용 기기를 시술받고 연구의 종료 시까지 중대한 연구계획서 위반 사항 없이 연구계획에 따라 임상연구를 완료한 피험자 전체로 정의한다. 임상시험 기간 중 유효성에 영향을 미치는 약물이나 대체치료를 받은 경우는 PP 분석군에서 제외한다.

PP 분석군에서 제외될 주요 연구계획서 위반 사항

1) 임상시험 기간 중 유효성에 영향을 미치는 약물이나 대체치료를 받은 경우

13.2.2. 안전성 평가분석군

- 연구참여 동의 후 연구에 참여하여 임상시험용기기로 시술을 받은 모든 피험자가 해당된다.

13.2.3. 누락자료(결측치)의 처리

유효성 평가변수에 있어서 결측치가 발생할 경우는 그 변수는 분석에서 제외한다.

13.3. 통계분석방법

13.3.1. 일반적 사항에 대한 분석

연속적인 측정치에 대한 자료는 평균, 표준편차, 중간값, 최소, 최대 및 피험자 수 등을 보여주는 표를 통해 요약 될 것이다. 범주형 측정치에 대한 자료는 숫자와 분율을 보여주는 표를 통해 요약될 것이다.

모든 통계분석은 SAS 통계 프로그램을 사용하여 시행 될 것이다

13.3.2. 일차 유효성 분석

- 월경통 개선율(%) - 시술 후 3개월

월경통 개선 성공률은 아래의 가설을 토대로 확인 될 것이다.

$$H_0: P_A - P_0 \le -\delta \ vs \ H_1: P_A - P_0 > -\delta$$

- P_A: 본 연구기기의 기대되는 성공률
- P_0: Reference 성공률 (90%)
- δ (>0): 비열등성 한계값 (12.3%)

위 가설은 $H_0: P_A \le 0.777 \ vs \ H_1: P_A > 0.777$ 로 표현가능하다. 따라서, 측정된 개선율(P_A)의 97.5% 단측 신뢰구간의 하한치가 0.777를 초과하면 본 임상의 유효성을 보여주는 것이다.

13.3.3. 이차 유효성 분석

본 연구에서 고려하는 이차결과변수에 대한 평가는 시술 후 1개월, 3개월 시점에 각각 이루어 질 것이다. '시술 만족도'는 시술일에 평가된다.

- 월경통 개선율(%) - 시술 후 1개월
  일차 평가변수와 동일한 월경통 개선율을 1개월 시점에서 구하고 95% 신뢰구간을 제시한다.
- 월경통 지표(Dysmenorrhea Score) - 시술 전, 후 1, 3 개월
  시술 전 대비 시술 후 1, 3개월 각 시점에서의 점수의 변화를 Wilcoxon signed rank test를 사용하여 비교 평가한다.
- 월경과다 지표(Menorrhagia Score) - 시술 전, 후 1, 3 개월
  시술 전 대비 시술 후 1, 3개월 각 시점에서의 점수의 변화를 Wilcoxon signed rank test를 사용하여 비교 평가한다.
- 삶의 질 측정(SF36-v2, UFS-QOL, SSS)
  각 설문지별 전체 및 개별항목 점수의 시술 전과 후의 비교는 Paired t-test 또는 Wilcoxon signed-rank test를 수행하여 분석할 것이다.
- 자궁 크기(cm^3)
  시술 전 대비 시술 후 1개월 및 3개월 각 시점에서의 자궁의 크기를 Paired t-test 또는 Wilcoxon signed rank test를 사용하여 비교 평가 한다.
- 시술 만족도
  시술 중 피험자가 느낀 통증 및 만족도 점수를 평균 및 표준편차로 요약 제시한다.

13.3.4. 안전성 분석

피험자로부터 수집된 모든 이상반응, 활력징후, 혈액검사 등에 근거하여 안전성 평가를 수행한다.

시술 후 3개월까지 수집된 모든 상기 안전성 변수 자료를 각 안전성 평가변수가 측정된 시점별, 각 피험자 별로 제시하고, 요약통계량을 제시한다. 피험자로부터 수집된 이상반응들은 MedDRA에 의해 코딩될 것이며, 이상반응, 이상의료기기반응, SAE, 사망, 임상시험 중단을 초래한 이상반응, 각 이상반응을 나타낸 피험자 수는 신체기관계(SOC, System Organ Class), 권장용어(PT, Preferred Term) 및 최대 중증도 별로 요약한다.

14. 임상시험 중지 및 탈락기준

임상시험 참여 동의 후에 참여 철회를 하는 경우나 시험책임자의 판단 하에 임상시험에의 참여가 피험자의 안전에 영향을 미친다고 판단되는 경우 임상시험의 참여를 중지하거나, 참여에서 탈락

시킬 수 있다.

14.1. 중지기준

① 임상시험 진행 중 관찰되는 상황이 임상시험을 계속 진행하는 것에 무리가 있다고 판단되는 경우에는 임상시험책임자가 임상시험을 중지할 수 있으며, 이 사실을 임상연구윤리위원회에 즉시 알리고 조기종료 및 일시중지에 대한 상세한 사유서를 제출해야 한다.
② 생명을 위협하는 중대한 이상반응/이상 의료기기반응이 발생하는 경우 또는 발생된 이상반응 처치를 위해서도 임상시험을 중지할 수 있다.

14.2. 탈락기준

① 피험자 또는 법정 대리권자가 임상시험 참여 중단을 요청하는 경우
② 안전성, 유효성 평가에 영향을 줄 수 있는 수술, 약물, 또는 의료기기를 병행하여 사용한 경우
③ 중대한 이상반응이 발생하여 임상시험의 지속참여가 불가능한 경우
④ 치료방법을 제대로 수행하지 않은 경우
⑤ 피험자가 시험자의 지시에 불응하거나 또는 동의서에 제시된 사항을 준수하지 않아 그 유효율의 평가에 영향을 미치는 경우
⑥ 임상시험과 관련이 없는 사유로 피험자가 사망하는 경우
⑦ 기타 임상시험 담당자가 임상시험 진행에 문제가 있다고 판단하는 경우

14.3. 중지의 처리

① 임상시험이 중지된 경우 중지사유 및 중지 전까지 진행된 임상시험 관련 자료를 기록, 보관하고 사유서를 제출한다.
② 임상시험이 중지된 경우 적절한 조치와 추적관찰이 이루어질 수 있도록 하여야 한다.
③ 중지 처리된 피험자는 안전성 분석에는 모두 포함될 것이며 유효성 평가에서는 제외된다.

14.4. 탈락의 처리

① 임상시험에서 중도탈락한 경우 탈락사유 및 탈락 전까지 진행된 임상시험 관련 자료를 기록, 보관하고 사유서를 임상연구윤리위원회에 제출한다.
② 피험자가 중도탈락하기까지 수집된 자료는 안전성 분석에는 모두 포함될 것이며 유효성 평가에서는 제외된다.

15. 부작용을 포함한 안전성의 평가기준, 평가방법 및 보고방법

15.1. 이상반응의 정의

① "이상반응(Adverse Event, AE)"이라 함은 임상시험 중 피험자에서 발생한, 바람직하지 않고 의도되지 않은 증후(sign, 예; 실험실적 검사치의 이상), 증상(symptom), 질병을 말하며, 해당임상시험용 의료기기와 반드시 인과관계를 가져야 하는 것은 아니다.
② “이상의료기기반응(Adverse Device Effect, ADE)”이라 함은 임상시험용 의료기기로 인하여 발생한, 모든 유해하고 의도되지 않은 반응으로서, 임상시험용 의료기기와의 인과관계를 배제 할 수 없는 경우를 말한다.
③ “예상하지 못한 이상의료기기반응(Unexpected Adverse Device Effect)”이라 함은 이용 가능한 의료기기 관련 정보(예를 들어 임상시험자자료집 또는 의료기기의 첨부문서)에 비추어 이상의료기기반응의 양상이나 위해의 정도에서 차이가 나는 것을 말한다.

15.2. 중대한 이상반응/이상의료기기 반응의 정의

중대한 이상반응(Serious Adverse Event)/이상 의료기기반응이라 함은 임상시험에 사용되는 의료기기로 인하여 발생되는 이상반응 중에서 다음 사항에 해당하는 경우를 말한다.

① 사망을 초래하거나 생명을 위협하는 경우
② 입원 또는 입원기간의 연장이 필요한 경우
③ 지속적 또는 의미 있는 불구나 기능저하를 초래하는 경우
④ 선천적 기형 또는 이상을 초래하는 경우

15.3. 이상반응의 평가

15.3.1. 중증도평가

이상반응이 발생하면 다음의 중증도(Severity) 평가기준에 의해 보고하여야 한다.

① 경증(mild)
피험자의 정상적인 일상생활(기능)을 저해하지 않고 최소한의 불편을 야기하며 피험자가 쉽게 견딜 수 있는 경우
② 중등증(moderate)
피험자의 정상적인 일상생활(기능)을 유의하게 저해하는 불편을 야기하는 경우
③ 중증(severe)
피험자의 정상적인 일상생활(기능)을 불가능하게 하는 경우

15.3.2. 임상시험용 의료기기와의 인과관계 평가

이상반응 발현 시 임상시험용 의료기기와의 관련성 여부는 시험자가 다음 기준에 의하여 평가하며, 시험자의 의견을 기술한다.

① 관련성이 명백함(definite)
② 관련성이 많음(probable)
③ 관련성이 의심됨(possible)
④ 관련성이 적음(possibly not)
⑤ 관련성이 없음(definitely not)
⑥ 평가불가능(unknown)

15.4. 이상반응의 평가기준

본 임상시험에서 이상반응은 임상시험 시작 전에 관찰되지 않은 증상이 발생하는 모든 바람직하지 않은 의학적 소견을 이상반응으로 분류한다. 예측되는 부작용도 이상반응으로 분류하며 이상반응의 정도를 경증, 중등증, 중증으로 분류하고 용어의 사용은 MedDRA의 'Preferred term' 및 'System organ class'를 이용하여 보고한다.

15.5. 예상되는 이상반응 및 주의사항

15.5.1. 예상되는 이상반응

- 통증
- 오심
- 구토
- 복부 팽만감(Abdominal tenderness)
- 부종
- 복부 경련(Abdominal cramping)
- 1~3도 화상
- 내부조직 열손상
- 다리와 엉덩이 통증
- 치료 전 이상의 질출혈
- 좌골신경손상
- 복부 및 골반 장기 손상(방광, 자궁, 장 등)
- 약물에 반응하지 않는 통증
- 비뇨기계 감염
- 배뇨장애
- 감염으로 인한 발열
- 조영제 사용에 대한 과민반응(어지러움, 오심, 구토, 가려움증, 두드러기, 작열감, 혈압강하, 심박동이상, 호흡곤란, 신장질환, 급성신부전 등)
- MR 검사 상 복부 근육 내 충혈성 변화
(복부 반흔이 있어 스카패치를 적용 후 시술한 경우에 한함.)

15.5.2. 사용 시 주의사항

■ 피험자 안전 정보

① 피험자 데이터를 올바로 식별하여 입력한다.
② 시스템 작동이 익숙해질 때까지 시스템을 사용하지 않는다.
③ 이미지 트랜스듀서의 과열을 방지하기 위하여 이미지를 보지 않을 때는 이미지를 고정시켜야 한다.
④ 이미지 트랜스듀서를 사용하지 않을 때는 시스템에서 음향 출력이 방출되지 않도록 한다. 그렇지 않으면 이미지 트랜스듀서가 과열될 수 있다.
⑤ 시스템을 사용하지 않을 때는 이미지 트랜스듀서를 고정시키거나 음향 출력을 꺼야 한다.
⑥ 시스템을 제세동기와 함께 사용하지 않는다. 이 시스템에는 제세동기 보호가 적용된 ECG 부품이 포함되어 있지 않다.
⑦ 살균제가 피험자에게 닿지 않도록 한다. 살균제가 피험자의 피부나 점막에 닿으면 감염을 일으킬 수 있다.

■ 적용상의 주의

① 시스템 손상 및 심각한 피험자 부상을 방지하기 위해 손상되거나 결함이 있는 이미지 트랜스듀서를 사용하지 않는다.
② 이미지 트랜스듀서가 손상될 수 있으므로 이미지 트랜스듀서 케이블을 구부리거나 잡아당기지 않는다.
③ 승인된 커플링 젤만 사용한다. 승인되지 않은 젤을 사용하면 이미지 트랜스듀서가 손상되고 보증이 무효화될 수 있다.
④ 이미지 트랜스듀서를 떨어뜨리지 않는다. 이미지 트랜스듀서를 사용하지 않을 때는 항상 안전한 장소에 보관한다.
⑤ 손상되거나 결함이 있는 이미지 트랜스듀서를 사용하면 예상치 못한 감전이 발생할 수 있으므로 이미지 트랜스듀서를 주의하여 사용한다.
⑥ 이미지 트랜스듀서를 사용하지 않을 때 시스템에서 음향 출력이 방출되지 않도록 한다. 그렇지 않으면 이미지 트랜스듀서가 과열될 수 있다.
⑦ 시스템을 사용하지 않을 때는 이미지 트랜스듀서를 냉각시키거나 음향 출력을 꺼야 한다.
⑧ 심각한 피험자 부상을 방지하기 위해 사용하는 이미지 트랜스듀서에서 날카로운 모서리나 거친 표면이 있는지 항상 검사한다.
⑨ 이미지 트랜스듀서를 적합하게 청소 및 살균 처리해야 질병의 전염이 방지된다. 감염통제 절차를 따라야 한다.
⑩ 사용 기간이 만료된 이미지 트랜스듀서 외피를 사용하지 않는다.
⑪ 윤활유가 도포된 콘돔을 외피로 사용하면 이미지 트랜스듀서가 손상될 수 있다.
⑫ 커플링 젤이 눈(또는 피험자의 눈)에 들어가지 않도록 한다.
⑬ 눈에 젤이 들어가면 깨끗한 물로 잘 씻어낸다.

15.6. 이상반응 보고방법

15.6.1. 이상반응교육

시험책임자는 시험담당자 및 피험자 또는 대리인에게 수술 또는 임상시험용 의료기기 사용 후 나타날 수 있는 모든 이상반응에 대하여 교육을 실시하고 사용 후 나타나는 모든 현상에 대하여 계획서에 명시 된 기한 내에 명시된 보고양식을 통해 보고하도록 교육을 실시한다.

15.6.2. 예상되는 이상반응의 기록

예상되는 이상반응 발생 시에 시험자는 다음과 같은 사항을 증례기록서에 기록한다.

- 이상반응 명칭
- 시작일 및 종료일
- 중증도
- 시험기기와의 인과관계
- 치료 내용 및 결과

15.6.3. 중대한 이상반응/이상의료기기반응 보고

시험자는 임상시험 기간 중 발생한 모든 중대한 이상반응/이상 의료기기 반응을 중대한 이상반응 기록지에 기록하고, 임상시험용 의료기기 사용의 관련성 여부와 상관없이 24시간 내에 의뢰자에 보고하여야 한다. 이 경우 피험자의 신상에 관한 비밀을 보호하기 위해 피험자의 신상 정보를 대신한 피험자 식별코드를 사용한다.

이외에도 시험자가 중대하다고 간주하거나 임상시험용 의료기기 사용과 연관 지을 수 있는 유의한 위험, 금기, 부작용, 주의사항을 시사하는 사건 등도 중대한 이상반응/이상의료기기반응으로 기록하고 의뢰자에 즉시 보고한다.

사망사례를 보고하는 경우 시험자는 의뢰자와 IRB에 부검소견서(부검을 실시한 경우만 해당한다)와 사망진단서 등의 추가적인 정보를 제출하여야 한다.

의뢰자는 임상연구윤리위원회 및 식품의약품안전청장에게 중대하고 예상하지 못한 이상반응(이상 의료기기반응, 중대한 이상반응/이상 의료기기반응을 포함)을 다음 각 호에서 정한 기간 내에 가능한 신속히 보고하여야 한다.

1) 사망을 초래하거나 생명을 위협하는 경우에는 시험책임자가 이 사실을 보고받거나 알게 된 날로부터 7일 이내, 이 경우 상세한 정보를 최초보고일로부터 8일 이내에 추가로 보고하여야 한다.
2) 그 밖의 중대하거나 예상하지 못한 모든 이상반응이 나타난 경우에는 시험책임자가 이 사실을 보고 받거나 알게 된 날로부터 15일 이내에 보고한다.
3) 의뢰자가 식품의약품안전처장에게 위 1)에 따라 이상의료기기반응을 보고하고자 하는 경우에는 의료기기 임상시험 관리기준(KGCP)의 별지 제 36호 서식에 따른 이상의료기기반응 보고서에 제출한다.

중대한 이상반응 및 이상의료기기반응 보고와 관련하여 추가적인 안전성 정보를 주기적으로 해당 이상반응이 종결(해당 이상반응의 소실 또는 추적조사의 불가 등)될 때까지 보고하여야

한다.

16. 피험자 안전보호에 관한 대책

16.1. 의료기기 임상시험 관리기준(KGCP)과 헬싱키선언

본 계획서에 규정된 절차는 시험 임상시험 연구자가 이 시험을 실시, 평가하고 결과를 기록하는 데 있어서 ICH-GCP 및 헬싱키선언의 기본정신을 준수하도록 작성되었다. 본 임상시험은 또한 국내법규(의료기기 임상시험 관리기준, KGCP)에 따라 시행될 것이다.

16.2. 임상연구윤리위원회(IRB)

임상시험을 시작하기 전에 연구자는 임상시험계획서, 피험자 동의서, 피험자 모집과 관련된 자료(예, 광고) 및 관련 서류를 임상연구윤리위원회에 제출하여 검토와 승인을 받아야 한다.
IRB와 식품의약품안전청의 승인이 필요한 계획서상의 변경사항이 생기면, IRB로부터 개정된 임상시험 계획서 및 개정된 피험자 동의서(해당되는 경우)의 검토 및 승인을 받기 전에는 임상시험계획서상의 변경 사항을 적용하지 않을 것이다. 피험자에게 발생한 즉각적 위험 요소를 제거하기 위한 임상시험 계획서의 개정은 식약청과 IRB에 가능한 한 빨리 공지하고 승인을 요청하는 것을 조건으로 즉시 적용될 수 있다. 모니터요원의 변경, 시험담당자의 변경, 응급 연락 전화번호의 변경 등과 같은 행정 절차와 관련된 계획서 변경은 IRB의 승인 이전이라도 바로 적용될 수 있다.
시험자는 관련규정이나 병원 내 절차에 따라 임상시험 결과보고서, 최신정보, 그리고 기타정보(예, safety update)를 임상연구윤리위원회에 제공해야 한다.

16.3. 피험자 동의서

임상시험을 위한 스크리닝/기저치 평가가 시행되기 전에 각 피험자(또는 피험자의 법정 대리인)으로부터 반드시 서면 동의서를 수령하여야 한다. 임상시험연구자는 시험을 시작하기 전 선정기준 및 제외기준을 모두 만족시킨 환자들을 대상으로 환자 및 그들의 보호자에게 시험에 관련된 모든 사항을 상세히 설명하고 모든 예측 가능한 결과에 대해 알 수 있는 충분한 시간을 주어야 한다. 서명된 동의서 사본은 피험자가 보관하고 원본은 연구자가 보관한다.
임상시험연구자는 임상 시험 참여에 동의한 모든 피험자들의 목록을 작성하여 보관하고 임상시험 의뢰자에게 제출해야 한다.
임상시험 관련 규정에 따라 피험자의 시험관련 자료가 의뢰자에 의해 사용될 것이라는 사실과 공개의 수준을 피험자에게 알려야 한다. 또한, 임상시험 모니터 요원 또는 감사자, IRB, 보건당국 실사자에 의해 피험자의 의학적 기록이 검토될 수 있다는 사실을 피험자에게 알려야 한다.
임상시험계획서가 개정될 경우, 계획서의 변경사항을 반영하기 위해 피험자 동의서와 피험자 동의 설명서가 개정될 수 있다. 피험자 동의서와 동의설명서가 개정되는 경우 IRB의 검토와 승인

을 받아야 하며, 새로 등록되는 피험자 및 현재 참여하고 있는 피험자에게 변경된 내용을 설명하고 개정된 동의서 양식에 서명을 받아야 한다.

16.4. 피해자 보상에 관한 규약

피험자가 본 임상시험에 참여하지 않았다면 받지 않았을 의료기기 사용 또는 임상적 절차로 인해 손상을 입은 경우에 병원의 표준절차에 따라 모든 처치를 받게 될 것이며, 임상시험과 관련된 손상일 경우 피해자보상규약 및 가입한 임상시험보험 약관에 따라 비용을 부담하게 될 것이다.

16.5. 피험자의 안전보호에 관한 대책

본 임상시험 지속 여부에 영향을 줄 수 있는 새로운 정보가 얻어지면 적시에 본인 또는 대리인에게 정보를 제공할 것이며, 연구자는 임상시험 참여 지속 여부를 피험자와 논의할 것이다.

16.6. 임상시험 후 피험자의 진료 및 치료기준

피험자가 이상반응으로 인해 임상시험에서 중도탈락하거나 임상시험 완료 후에도 부작용 및 이상반응으로 인한 잔여증상이 있는 경우, 발생된 이상반응에 대하여 병원의 표준 의료지침에 따라 적절한 의학적 조치를 수행하여 회복될 때 까지 충분한 치료를 받게 될 것이며, 이상반응 없이 임상시험이 종료된 피험자들은 임상 시험 후에는 병원의 표준절차에 따라 일반적인 임상관찰을 받게 될 것이다.

16.7. 임상시험실시기관

임상시험실시기관의 장은 해당 임상시험의 실시에 필요한 임상시험실, 설비와 전문 인력을 갖추어야 하고, 긴급 시 필요한 조치를 취할 수 있도록 하는 등 해당 임상시험을 적절하게 실시할 수 있도록 하여야 한다.

16.8. 시험자

① 시험자라 함은 시험책임자, 시험담당자, 임상시험조정자를 말한다. 시험자는 의뢰자와 합의되고 임상연구윤리위원회 및 식품의약품안전처장의 승인을 득한 임상시험계획서를 준수하여 임상시험을 실시하여야 한다.

② 임상시험 중 또는 임상시험 이후에도, 시험자는 임상적으로 의미 있는 실험실적 검사치의 이상을 포함하여 임상시험에서 발생한 모든 이상반응에 대해 피험자가 적절한 의학적 처치를 받을 수 있도록 조치하여야 하고, 시험자가 알게 된 피험자의 병발질환에 대해 의학적 처치가 필요한 경우 이를 피험자에게 알려주어야 한다.

③ '15.6.3. 중대한 이상반응/이상 의료기기반응 보고' 항에 따라, 시험담당자는 이상반응의 인지 및 시험책임자에 대한 보고 의무를 가지며, 시험책임자는 시험담당자로부터 보고받은 이상반응을 의뢰자에 보고할 의무를 가진다.

④ 시험자는 임상시험계획을 정확히 분석 및 숙지하고, 대상 피험자의 문제점을 적극적으로 대응한다.

16.9. 의뢰자

① 임상연구의 계획•관리•재정 등에 관련된 책임을 갖고 있는 자로, 통상 의료기기 임상시험의 경우 의료기기 제조업자(수입자를 포함한다)를 말한다.
② 임상시험대상, 시험방법, 증례보고서의 서식과 내용 등이 임상시험계획서의 절차에 따라 이루어지도록 해야 한다.
③ 의뢰자의 점검 계획과 절차는 임상시험의 중요도, 피험자 수, 임상시험의 종류와 복잡성, 피험자에게 미칠 수 있는 잠재적인 위험의 정도 및 이미 확인된 임상시험 실시상의 문제점 등에 따라 결정되어야 한다.

17. 기타 임상시험을 안전하고 과학적으로 실시하기 위하여 필요한 사항

17.1. 비밀보장

17.1.1. 데이터

연구자는 의뢰자나 임상시험 모니터요원이 제공한 연구계획서와 관련한 모든 정보들에 대하여 비밀을 준수하여야 한다. 단, 법률이나 관련규정에 따라 IRB나 피험자, 또는 보건당국이 공개를 요구하는 경우는 예외로 한다.

17.1.2. 피험자의 익명성

임상시험에 참여한 피험자의 익명성이 반드시 보장되어야 한다. 피험자의 식별은 피험자의 이니셜과 증례기록서에 지정된 피험자 번호와 임상시험 모니터 요원에게 제출된 다른 자료로 이루어져야 한다. 피험자에게 모든 시험 자료가 컴퓨터에 저장되고 엄격히 비밀사항으로 다루어진다는 것을 알려준다. 서명을 받은 피험자동의서는 시험 책임자가 보관한다. 임상시험 책임자는 피험자번호 및 피험자 명이 기록된 리스트를 갖추어 놓아 나중에 기록을 찾을 수 있도록 한다. 피험자 동의서와 피험자 리스트는 3년간 보관한다.
피험자 식별을 위한 자료들은 연구자에 의해 엄격히 비밀이 보장되어야 한다. 단, 보건당국이나 임상시험 모니터요원, 의뢰자 또는 지정된 대리인의 감사를 받기 위해 필요한 경우에는 예외로 한다.

17.2. 연구계획서의 준수 및 연구계획서 변경

임상시험 책임자나 의뢰자 어느 쪽도 상대방의 동의 없이는 시험 도중 본 연구계획서의 내용

을 변경 할 수 없다. 연구계획서의 모든 변경은 의뢰자와 논의되어야 하며, 변경 계획서는 의뢰자가 작성한다. 시험자는 피험자에게 위해가 발생되는 것을 즉각적으로 막기 위한 경우를 제외하고는 이러한 연구계획서 변경에 대하여 임상연구윤리위원회로부터 검토 승인을 얻기 전에는 변경된 내용을 적용해서는 안 된다. 중대한 시험계획서 위반에 대해서는 증례기록서에 기록해야 한다.

만일 즉각적으로 피험자에게 위해가 발생하지 못하게 하기 위하여 임상연구윤리위원회의 승인을 얻기 전에 이러한 시험계획서의 변형이나 변경을 적용하게 되는 경우, 가능한 빨리 이러한 변형이나 변경에 대하여 임상연구윤리위원회(추후 검토 승인을 위하여), 식품의약품안전처(관련규정에서 요구하는 경우)에 제출하여야 한다.

임상시험계획서의 중요한 변경은 피험자의 안전에 영향을 미치는 변경이나 연구범위의 변경, 임상시험의 과학적 질의 변경, 실험적 디자인의 변경, 평가변수(들)의 변경, 피험자 수의 변경, 피험자 선정기준의 변경을 포함한다. 이러한 변경들은 반드시 기록되어야 하고 변경이 타당함을 입증하기 위한 자료로 제공될 것이다.

의뢰자는 이러한 변경들을 반드시 개정된 임상시험계획서로 준비하여야 하며, 의뢰자와 연구자의 공동 승인에 의해서만 시행될 수 있다. 개정된 임상시험계획서는 시행 이전에 해당 IRB나 관련 당국의 승인을 받아야 한다.

임상시험계획서 변경 절차는 IRB 승인의 진행과 병행하여 허가 당국에 최초 승인본에 대한 변경본이 제출될 것이다. 변경된 임상시험계획서가 동의서의 변경을 필요로 하는 경우 반드시 IRB의 승인을 얻어야 한다.

임상시험에 참여한 특정 피험자의 명백하고 급박한 위험을 배제하기 위한 임상시험계획서로부터의 긴급한 일탈은 피험자의 안전과 안녕을 위해 중대한 것으로 간주되며 오직 해당 특정 피험자의 경우에만 시행될 수 있다

만일 시험계획서 변경 내용이 사소한 것이면 시험자는 임상연구윤리위원회에 통보하는 것으로 충분하다. 그러나 시험디자인을 본질적으로 변경하거나 피험자에게 위험 가능성이 증가되는 경우라면, 1)피험자 동의서를 수정하여 임상연구윤리위원회에 제출하여 검토 승인을 받아야 하고 2)이러한 변경으로 피험자들에게 영향을 주게 된다면 이미 시험에 모집 된 피험자들로부터 새로 변경된 동의서에 다시 동의를 얻어야 하며 3)새로 모집하는 피험자들로부터는 새로운 동의서를 사용하여 동의서를 얻어야 한다.

17.3. 임상시험 모니터링

임상시험 시작 전에 본 임상시험을 위해 의뢰자, 시험책임자, 임상시험수탁기관의 담당자 등이 연구자회의나 임상시험개시모임을 개최할 것이다. 이 회의에서는 임상시험계획서, 임상시험절차의 수행, 증례기록서의 작성, 검사 방법 등에 관한 자세한 논의가 이루어질 것이다. 이러한 회의나 모임에 참석할 수 없는 연구자나 나중에 임상시험에 참여하게 되는 연구자는 의뢰자나 시험책임자, 또는 그에게 위임을 받은 자에 의해 적절한 교육을 받아야 한다.

의뢰자 및 임상시험수탁기관은 시험기관에 적절히 관련지침 및 자료를 확보하고 본 임상시험을 담당할 모니터요원을 지정하여 임상시험 시작 전과 시험기간 동안 자료의 완결성을 위한

모니터링을 하도록 한다. 시험이 KGCP에 따라 실시되고 시험자료가 국내,외에서 등록 시 인정될 수 있도록 하기 위해서 외부 기관에 의뢰하여 모니터링 및 점검(audit)을 실시할 수 있다. 모니터요원은 임상시험을 시작하기 전에 연구자에게 모니터링 계획에 대해 설명하고, 모니터링 시에는 연구자가 임상시험계획서와 관련 규정에 따라 임상시험을 수행하는 지 확인을 한다. 임상시험 모니터요원은 일상적으로 연구자를 접촉하며, 임상시험의 다양한 기록들을 모니터링 하는 것이 승인된다.

기록된 데이터의 완결성과 일관성 및 정확성을 입증하고 기록된 데이터가 임상시험 계획서에 충실하였음을 입증하기 위해 임상시험 기간 내내 정기적으로 증례기록서를 모니터링 하는 것은 임상시험 모니터요원의 책임이다. 발견된 사항에 대해서는 연구자와 적절히 논의하도록 한다. 연구자는 임상시험 과정에서 발견한 어떤 사항에 대해서도 임상시험 모니터요원에게 적절하게 알리고 모니터링 활동에 협력하여야 한다.

17.4. 시험결과의 기록 및 이용

17.4.1. 증례기록서와 근거문서

임상시험에서 요구되는 모든 정보들은 해당 증례기록서 페이지에 기재되어야 한다. 또한, 연구자는 데이터의 정확성을 보증하기 위해 각각의 증례기록서에 있는 연구자 서명란에 반드시 서명하여야 한다.

연구자는 임상시험과 관련한 모니터링, 점검, IRB 검토, 실사 시 근거자료와 근거문서의 직접 열람을 허용해야 한다. 각 피험자의 원본 증례기록서는 임상시험 모니터요원에 의해 임상시험 기관에서 근거문서와 비교하여 확인될 것이다.

시험책임자는 임상시험 기본문서를 유지하고 제공할 의무가 있다. 임상시험기본문서란 임상시험의 수행과 그로부터 얻어진 자료의 질에 대하여 개별적 또는 전체적으로 평가가 가능하도록 해주는 문서를 말한다. 임상시험기본문서는 근거문서를 포함하는데, 근거문서는 임상시험에 관련되는 약국, 병리검사실 및 의학적/기술적 담당부서에게 보관하고 있는 병원기록, 의무기록, 임상실험실검사 결과, 피험자일지, 각종 검사지와 기록지를 포함한다.

근거문서, 증례기록서 및 기타 임상시험관련 문서는 시험기관에 보관되어 있어야 하며, 모니터링이 완료된 증례기록서는 시험책임자가 원본을 회수하고, 시험기관에 사본을 보관한다. 연구자는 임상시험과 관련한 모든 기록을 식품의약품안전청의 실태조사가 완료될 때까지 보관하고, 실태조사가 완료된 후에는 의뢰자가 정한 기한까지 보관한다.

17.4.2. 임상연구자료의 보관

모든 증례기록서와 제공되는 자료 및 관리 기록들은 본 제품의 품목허가일로부터 3년간 보관하여야 한다. 어떤 임상연구 자료도 시험책임자의 사전 서면 동의 없이 파기되거나 다른 장소로 옮겨져서는 안 된다. 연구자가 근무지 이전이나 은퇴 및 그 밖의 다른 사유로 인해 해당 임상시험에서 배제되는 경우 시험책임자에게 알리고 적절한 해결 방안에 동의하여야 한다.

시험자는 임상시험과 관련한 임상의료기기 사용기록, 증례기록서 사본 및 근거자료 등을 국내 규정에 따라 정한 기간 3년 동안 보존해야 한다.

17.4.3. 시험결과의 이용

본 임상시험계획서에 서명함으로써 시험담당자는 본 시험의 결과를 등록, 발표 및 의료기기 전문가들을 위한 정보제공 등의 목적으로 사용하는데 동의한 것이다. 본 임상시험의 결과를 학술잡지 또는 학회지에 발표하기 전에 임상시험책임자는 발표내용을 검토할 권리가 있다.
임상시험 종료 후 연구자와 의뢰자는 출판에 대한 최신 전략을 세우기 위해 모든 결과물과 분석자료 및 보고서를 재검토한다. 모든 출판은 연구자와 의뢰자 간의 협조에 의해 준비되어야 한다.
임상시험 중 발생한 정보들은 의뢰자의 소유이며, 의뢰자의 서면 승인 없이 출판되어서는 안 된다.

17.5. 의뢰자와 임상시험기관 장과의 계약서

① 의뢰자는 임상시험기관의 장과 문서로써 임상시험계약을 체결하여야 하며, 다기관임상시험을 실시하려는 경우에는 하나의 임상시험기관의 장과 총괄하여 계약할 수 있다.
② 임상시험 계획서에는 연구비의 규모 및 지급 방법, 조기종료 및 시험 중단 시 미사용 연구비의 반납 등 임상시험의 재정에 관한 사항, 업무의 위임 및 분장에 관한 사항 및 의뢰자와 임상시험기관의 장의 의무 사항을 포함하여야 한다.

17.6. 시험책임자의 이력사항

① 시험자는 임상시험의 적정한 실시를 위하여 임상시험기관 표준작업지침서에서 정하는 바에 따른 임상시험 실시에 필요한 교육·훈련 및 경험을 갖추어야 한다.
② 시험자는 임상시험계획서, 임상시험자 자료집, 그 밖의 의뢰자가 제공한 의료기기 관련 정보에 적힌 임상시험용 의료기기의 적절한 사용방법을 자세히 알아야 한다.
③ 시험자는 관계 법령을 자세히 알고 준수하여야 한다.
④ 시험책임자 및 임상시험기관의 장은 의뢰자의 모니터링 및 점검에 따라야 한다.
⑤ 중요한 임상시험 관련 업무를 시험담당자에게 위임한 경우 시험책임자는 시험담당자의 명단을 확보·유지 하여야 한다.

17.7. 임상시험용 의료기기의 사용 및 관리

① 임상시험용 의료기기는 해당 임상시험실시기관의 장이 지정한 자가 관리한다. 임상시험용 의료기기는 기재사항에 기술되어있는 대로 취급, 저장 하며 "임상시험용"이라는 문구가 있어야 한다. 임상시험용 의료기기 관리자는 임상시험에 사용되는 의료기기에 대해 인수, 재고관리, 반납 등의 업무를 수행하고 관련 기록을 유지하여야 한다.

17.8. 임상시험용 의료기기의 공급과 취급

① 의뢰자는 임상시험계획서에 대한 임상연구윤리위원회와 처장의 승인을 얻기 이전에는 임상시험용 의료기기를 관리자 등에게 공급해서는 아니 된다.
② 의뢰자는 관리자등이 임상시험용 의료기기를 취급하고 보관하는 방법에 대해 문서화된 절차를 가지고 있어야 하며, 이 절차에는 적절하고 안전한 인수, 취급, 보관, 미사용 임상시험용 의료기기의 피험자로부터의 반납 및 의뢰자에 대한 반납 등에 대한 방법이 포함된다.
③ 임상시험용 의료기기를 적시에 공급하여야 하며, 임상시험기관으로의 공급, 임상시험기관의 인수, 임상시험기관으로부터의 반납 및 폐기에 관한 기록을 유지하여야 한다.
④ 의뢰자는 임상시험용 의료기기에 고장 등 문제가 발생하거나 임상시험의 종료 또는 사용기간의 만료 등에 의한 임상시험용 의료기기의 회수체계를 확립하고 이를 문서화하여야 한다.

18. 참고문헌

1. Xin Xhang et al. Effective ablation therapy of adenomyosis with ultrasound-guided high-intensity focused ultrasound. Int J Gynaecol Obstet. 2014 Mar;124(3):207-11.
2. Fan TY et al. Feasibility of MRI-guided high intensity focused ultrasound treatment for adenomyosis. Eur J Radiol. 2012 Nov;81(11):3624-30.
3. Zhou M et al. Ultrasound-guided high-intensity focused ultrasound ablation for adenomyosis: the clinical experience of a single center. Fertil Steril. 2011 Mar 1;95(3):900-5
4. Fukunishi H et al. Early results of magnetic resonance-guided focused ultrasound surgery of adenomyosis: analysis of 20 cases. J Minim Invasive Gynecol. 2008 Sep-Oct;15(5):571-9.
5. Zhang L, Chen WZ, Liu YJ, et al. Feasibility of magnetic resonance imaging guided high intensity focused ultrasound therapy for ablating uterine fibroids in patients with bowel lies anterior to uterus. European Journal of Radiology 2010;73:396–403.
6. Tempany CM, Stewart EA, McDannold N, Quade BJ, Jolesz FA, Hynynen K. MR imaging-guided focused ultrasound surgery of uterine leiomyomas: a feasibility study. Radiology 2003;226:897–905.
7. LeBlang SD, Hoctor K, Steinberg FL. Leiomyoma shrinkage after MRI-guided focused ultrasound treatment: report of 80 patients. American Journal of Roentgenology 2010;194:274–80.
8. Buttram VC Jr, Reiter RC. Uterine leiomyomata: etiology, symptomatology, and management. Fertil Steril 1981;36:433-45.
9. Hong Jin Hwa et al. Uterine Artery Embolization for Leiomyoma. Obstetrics & Gynecology Scienc 47(2004) 481-486
10. Seung Yol Lee et al. Clinical Review on 93 Cases of Laparoscopic Myomectomy. Obstetrics & Gynecology Science 47(2004) 6:1107-1112
11. Eun Seop Song. Ultrasound imaging guided high intensity focused ultrasound (HIFU) may be a safe tool to ablate uterine myoma. Obstetrics & Gynecology Science 53(2009) 8:843-849.

12. W Wang et al. Safety and efficacy of high intensity focused ultrasound ablation therapy for adnomyosis. Academic Rediology. 2009;16(11):1416-1423.
